# Contrasting aerosol–cloud feedbacks across a warming Arctic

**DOI:** 10.1038/s41561-026-02114-x

**Published:** 2026-09-28

**Authors:** Samuel De Xun Chua, Herbert G. Hartl, Matthew Boyer, Jakob Boyd Pernov, Radovan Krejci, Peter Tunved, Paul Zieger, Julia Kojoj, Andreas Massling, Henrik Skov, Lynn M. Russell, Sourita Saha, Tak Chan, Eija Asmi, Ville Vakkari, John Backman, Antti-Pekka Hyvärinen, Christina J. Williamson, Ilona Jaakkola, Mikko Sipilä, Nina Sarnela, Veli-Matti Kerminen, Martha Arbayani Zaidan, Tuukka Petäjä, Katrianne Lehtipalo

**Affiliations:** 1 Institute for Atmospheric and Earth System Research, Faculty of Science, University of Helsinki, Helsinki, Finland; 2 Department of Environmental Science, iClimate, Arctic Research Cluster, Aarhus University, Roskilde, Denmark; 3 Extreme Environments Research Laboratory, Ecole Polytechnique Fédérale de Lausanne, Sion, Switzerland; 4 School of Earth and Atmospheric Sciences, Queensland University of Technology, Brisbane, Queensland Australia; 5 Department of Environmental Science, Stockholm University, Stockholm, Sweden; 6 Bolin Centre for Climate Research, Stockholm University, Stockholm, Sweden; 7 Scripps Institution of Oceanography, University of California San Diego, La Jolla, CA USA; 8 Environment and Climate Change Canada, Toronto, Ontario Canada; 9 Atmospheric Composition Research, Finnish Meteorological Institute, Helsinki, Finland; 10 Atmospheric Chemistry Research Group, Chemical Resource Beneficiation, North-West University, Potchefstroom, South Africa; 11 Department of Computer Science, Faculty of Science, University of Helsinki, Helsinki, Finland

**Keywords:** Climate change, Atmospheric science, Cryospheric science

## Abstract

In the rapidly warming Arctic, the feedback between aerosols and clouds is a major source of uncertainty in projections of future climate. One source of this uncertainty is the role of new particle formation, which produces aerosol particles that can subsequently grow into cloud condensation nuclei, thereby influencing Arctic cloud formation. Here harmonized field aerosol observations across the Arctic spanning 2003–2024, alongside satellite-derived data, were analysed to quantify trends in new particle formation and estimate their climatic implications across the Arctic region. Three distinct aerosol regimes, with contrasting contributions of new particle formation to cloud condensation nuclei populations, were identified: the High Arctic, the Maritime Arctic and the Continental Arctic. In these three regimes, temperature-sensitivity estimates based on the intermediate future warming scenario suggest that by 2090–2100, cloud condensation nuclei concentrations associated with new particle formation activity could increase by 3–16% in the High Arctic but decline by 15–31% in the Maritime Arctic. These contrasting estimates indicate that aerosol–cloud feedbacks will evolve differently across the mosaic of Arctic aerosol regimes.

## Abstract (web summary)

Arctic observations over 2003–2024 identify three aerosol regimes with contrasting responses to warming and cloud condensation nuclei trends. Model projections suggest that the aerosol–cloud feedbacks will evolve differently across the Arctic by 2100.

## Main

Nowhere are the implications of climate change more apparent than in the rapidly warming Arctic^1,2^—broadly defined as north of 65° N. Changes in ecosystems, sea-ice cover, glacial extent and atmospheric circulation here have global implications^3–6^. However, a major challenge in predicting these changes lies in our poor understanding of Arctic clouds. Depending on their altitude, structure and season, clouds could either warm or cool the Arctic environment, potentially exacerbating or mitigating the warming trend there^7–9^.

Aerosol–cloud interactions, including the role of aerosol particles as cloud condensation nuclei (CCN) for liquid clouds and as ice-nucleating particles (INP) for ice and mixed-phase clouds, are an important but insufficiently represented part of the Arctic climate system^9,10^. Thus, this Article focuses on CCN, as new particle formation (NPF) is an important local source of CCN in the Arctic. The research consensus is that higher CCN concentrations lead to more and smaller cloud droplets that increase cloud reflectivity and cloud lifetime^11,12^. In the clean polar atmosphere, CCN concentrations can be so low at times that they become the limiting factor for cloud formation, so even minor changes in the aerosol budget could have disproportionately large climatic impacts^12^.

One of the uncertainties in projecting future CCN concentrations is how the contribution from new particle formation processes, that is, particles that form in the atmosphere from gas-phase precursor vapours, may change. On one hand, NPF intensity could increase due to changing meteorology^13^, retreating sea ice^14,15^ or increasing emissions of chemical precursor vapours (for example, iodine- or sulfur-containing compounds and oxidants)^15–17^, thereby increasing the local aerosol concentrations and leading to more potential CCN. On the other hand, the potential increase in CCN number concentrations may be overstated, as higher aerosol concentrations from either local or transported sources would also enhance the condensation sink^18–20^. An increased condensation sink, defined as the rate at which vapour molecules are scavenged by pre-existing aerosol particles, would limit the formation and subsequent growth of newly formed particles to CCN-relevant sizes^21,22^. Moreover, various NPF-related physico-chemica characteristics, such as precursor volatility or reaction rates, have nonlinear temperature dependences^23,24^, thus adding an layer of complexity in a warming environment.

**Fig. 1:**
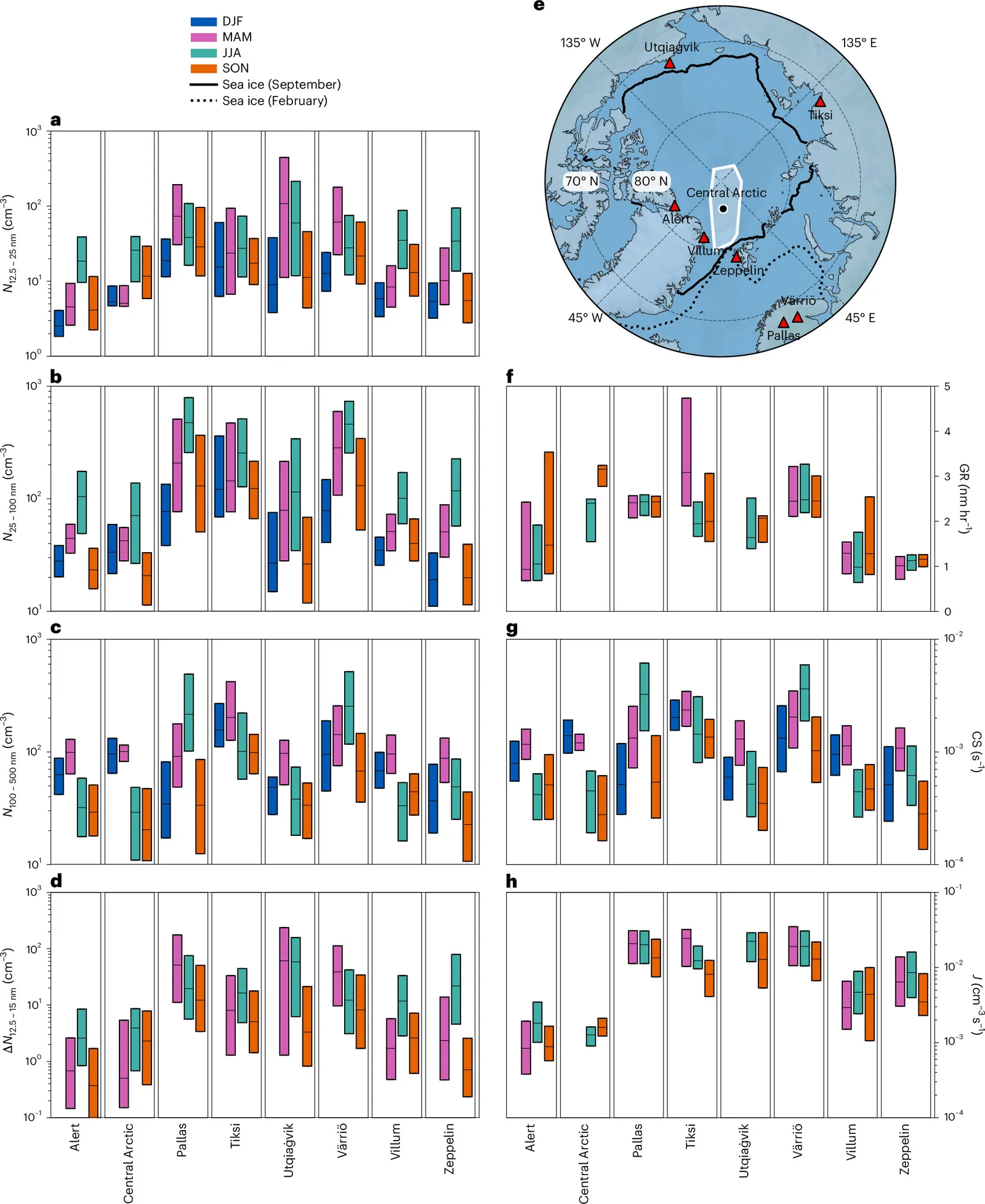
Measured aerosol parameters across eight Arctic locations. The aerosol parameters were measured at Alert, Central Arctic, Pallas, Tiksi, Utqiaġvik, Värriö, Villum and Zeppelin with more information about the sites in Supplementary Table 4. The boxplots show the median and interquartile range with December–February (DJF) in blue, March–May (MAM) in violet, June–August (JJA) in green and September–November (SON) in orange. **a**, Nucleation mode particles concentration: ${N}_{12.5-25{\mathrm{nm}}}$. **b**, Aitken-mode particles concentration: ${N}_{25-100\mathrm{nm}}$. **c**, Accumulation-mode particles concentration: ${N}_{100-500\mathrm{nm}}$. **d**, Diurnal variation in 12.5–15 nm particle concentration: ${\Delta\,N}_{12.5-15\mathrm{nm}}$. **f**, Growth rate at 15–20 nm: ${\rm{GR}}$. **g**, Condensation sink: ${\rm{CS}}$. **h**, Formation rate at 15 nm: J. **e**, Map of study area with the various Arctic sites marked by red triangles, a ‘Central Arctic’ polygon that shows the extent of the cruise campaigns and maximum (February) and minimum (September) sea-ice extent from 1981 to 2010 medians^75^. Basemap data in **e** from Natural Earth (https://www.naturalearthdata.com).

At the vanguard of addressing these uncertainties are the long-term observations speckled across the Arctic (Fig. 1). Whereas their records have been invaluable for documenting phenomena such as Arctic haze or NPF^25–33^, inter-site comparisons have previously been limited either in time or space. Building upon those past efforts, we harmonized the datasets for various aerosol microphysical properties from different Arctic locations (Fig. 1) by applying recently developed methodologies consistently to the measurement records. Altogether, a combined dataset spanning 2003–2024 across eight different Arctic locations, albeit with varying data coverage periods between sites, was compiled to provide new insights into the spatio-temporal variability of aerosol populations and NPF activity across the Arctic.

## Latitudinal and seasonal gradients in Arctic aerosols

Our harmonized dataset of particle number size distribution, constrained to 12–500 nm, revealed two spatio-temporal patterns: a north–south latitudinal contrast between the higher-latitude locations (Alert, Central Arctic, Villum and Zeppelin) and the lower-latitude continental sites (Tiksi, Pallas, Värriö, Utqiaġvik) and a seasonal transition from winter in December to summer in August reflecting a shift of aerosol origins from transported particles to those locally sourced.

As Aitken- and accumulation-mode particles are within the size range suitable for activation into CCN, their number concentrations are highly relevant to CCN contribution. From December to May, the Arctic atmosphere was dominated by long-range transport of accumulation-mode aerosols from lower latitudes, mainly of anthropogenic origins, that contribute to Arctic haze^28,34–36^. Due to the higher exposure to anthropogenic influences, both Aitken- and accumulation-modes particle concentrations were generally higher in the southern sites than at the northern locations (Fig. 1 and Supplementary Table 1). During a common 2010–2019 window, significant declines (*p* < 0.1) in accumulation-mode concentrations during December–February were observed at least at Alert, Pallas, Värriö, and Zeppelin (Supplementary Table 2). One possible reason could be the reduction in anthropogenic emissions across Eurasia and North America transported to the Arctic, either at source or due to changing atmospheric patterns^13,37–39^.

During June–August, and, to a smaller extent, March–May, local processes become an important contributor to Aitken-mode particles and, correspondingly, CCN^13,16,28,40,41^. During this sunlit period, the median concentration of Aitken-mode particles in the southern sites ranged ~79–474 cm^−3^, whereas that in the northern locations was lower at ~42–116 cm^−3^. As a proxy for NPF intensity, ${\Delta\,N}_{12.5-15\mathrm{nm}}$, defined as the difference between the daily peak particle number concentration in the 12.5–15 nm size bin from that during the daily background hours, was larger at the southern sites (8.1–61 cm^−3^) than at the northern locations (0.5–22 cm^−3^). This latitudinal variability was also observed with the NPF-related quantities of formation rates of 15 nm particles (${J}_{15}$) and growth rates of particles from 15 to 20 nm (${\mathrm{GR}}_{15-20}$). During spring and summer, ${J}_{15}$ was ~12–26 × 10^−3^ cm^−3^ s^−1^ at the southern sites, which was almost an order of magnitude higher than the rates of ~0.8–8.5 ×10^−3^ cm^−3^ s^−1^ at the northern locations. Likewise, ${\mathrm{GR}}_{15-20}$ was higher at the southern sites ( ~ 1.6–3.1 nm h^−1^), compared with ~1.0–1.1 nm h^−1^ at Villum and Zeppelin. In summary, NPF activity—in terms of formation and growth rates—was higher at the southern continental sites than at the northern locations.

Given the importance of the contribution of NPF to CCN in the Arctic, the ongoing question has been to quantify past and future trends in NPF. From the common 2010–2019 window, Arctic-wide declines in condensation sink values were observed across all seasons (Supplementary Fig. 1 and Supplementary Table 2). In theory, this reduction of condensation sink, associated with lower concentrations of larger particles, could enhance NPF activity due to reduced competition between nucleation and growth processes^42–44^. However, significant positive trends (*p* < 0.1) in ${\Delta\,N}_{12.5-15\mathrm{nm}}$ were observed only at a few sites: Alert (26 [interquartile range (IQR): 20–32] % yr^−1^), Villum (19 [IQR: 16–22] % yr^−1^) and Zeppelin (12 [IQR: 7.5–17] % yr^−1^) during March–May and Alert (16 [IQR: 12–20] % yr^−1^), Villum (14 [IQR: 11–17] % yr^−1^) and Pallas (12 [IQR: 8.1–16] % yr^−1^) during June–August. Furthermore, besides the increase in ${J}_{15}$ at Pallas observed during June–August (5.3 [IQR: 2.4–8.2] % yr^−1^, *p* < 0.1), there were no significant changes in either ${J}_{15}$ or ${{GR}}_{15-20}$ values. Thus, it appeared that the changes in NPF parameters during 2010–2019 had not been as uniform across sites and seasons.

## Mapping the three types of aerosol regimes

**Fig. 2:**
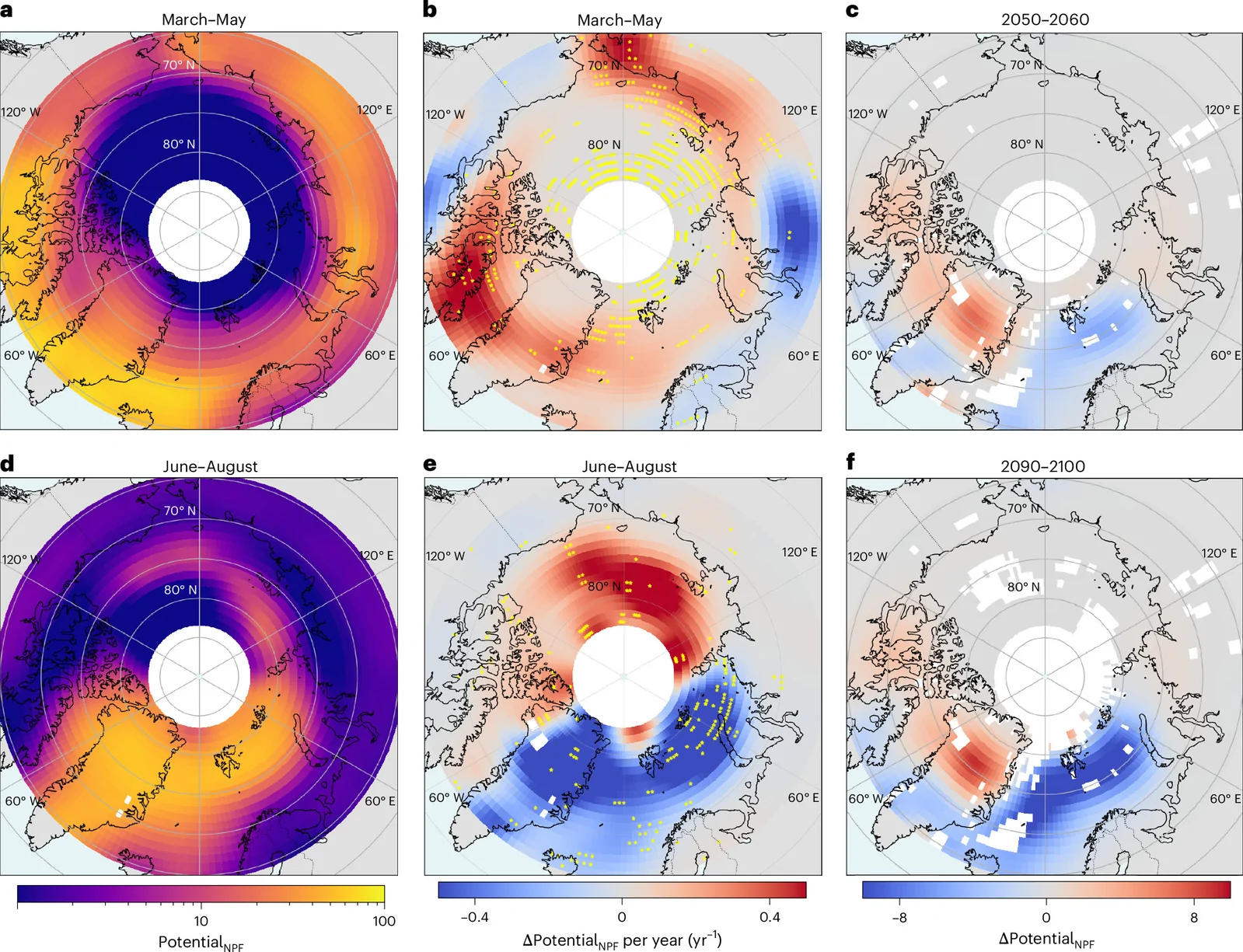
NPF potential, historical trends and future estimates across the Arctic. **a**,**d**, Medians of Potential_NPF_ averaged over 2006–2021 as estimated from satellite-based proxies during March–May (**a**) and June–August (**d**). Potential_NPF_ ranged (0,100) with 0 and 100 representing infinitely low and high potential of NPF events occurring, respectively. **b**,**e**, Yearly change in Potential_NPF_ over 2006–2021 during months of March–May (**b**) and June–August (**e**) expressed as percentage change per year. Pixels where trend is statistically significant (*p* < 0.1) are marked by yellow dots. **c**,**f**, The temperature-sensitivity estimates of change in Potential_NPF_ by 2050–2060 (**c**) and 2090–2100 (**f**) relative to the 2006–2021 baseline period, considering only the projected temperature change under the Shared Socioeconomic Pathway (SSP)2–4.5 scenario. White pixels indicate areas where data was unavailable. Basemap data from Natural Earth (https://www.naturalearthdata.com).

Despite this laboriously collected dataset, the uneven spatio-temporal data coverage had limited interpretations to only broad latitudinal or seasonal patterns, representing the challenge in building the pan-Arctic narrative^28,29^. To address these limitations, we developed a novel metric, Potential_NPF_, and used it to derive a 15-year (2006–2021) dataset of monthly maps spanning the Arctic from 65° N to 83° N (Fig. 2 and Supplementary Fig. 2). Briefly, Potential_NPF_ estimated the potential for NPF events—objectively quantified by an increase in ${\Delta N}_{12.5-15{\mathrm{nm}}}$ values. Potential_NPF_ was derived from monthly satellite-based environmental variables, including ultraviolet radiation (${\rm{UV}}$), water vapour content ($[{\rm{H}}_2{\rm{O}}]$), atmospheric aerosol loading ($\sigma_{\mathrm{ext}}$), vegetation indices ($[{\rm{NDVI}}]$) and sea-ice concentration ($[{\rm{sea}}\,{\rm{ice}}]$), together with surface air temperature (*T*) obtained from a reanalysis dataset. These predictors were selected because they represented proxies of environmental conditions affecting NPF processes, including photochemistry (${\rm{UV}}$), meteorological conditions ($[{\rm{H}}_2{\rm{O}}]$, *T*), aerosol sinks (${\sigma }_{\mathrm{ext}}$) and precursor vapour availability ($[{\rm{NDVI}}]$, $[{\rm{sea}}\,{\rm{ice}}]$). The resulting metric was scaled such that values of 0 and 100 corresponded to conditions of infinitely low and infinitely high NPF potential, respectively. Additional details and justification are provided in Supplementary Text 2.

The Potential_NPF_ maps corroborated the spatio-temporal gradients observed at the monitoring sites (Fig. 2a,d). From October to February, Potential_NPF_ remained ≤10, but following the polar sunrise in March, Potential_NPF_ increased to maxima of up to ~80 from March to August, although the timing of these peaks varied across regions. (Supplementary Fig. 2). Specifically, southern areas—approximately south of 70° N—had earlier peaks during March–May, whereas northern areas and the open oceans—such as the Barents Sea and Greenland Sea—showed a later peak in June–August, in agreement with the observed climatology (Supplementary Table 1 and refs. ^28,29^). During 2006–2021, March–May trends indicated increases in Potential_NPF_ of 0–0.5 yr^−^^1^ across most of the oceanic and coastal Arctic (Fig. 2b); this springtime enhancement of NPF activity was supported by observed increases at the high latitude sites of Alert, Villum and Zeppelin too. However, June–August trends in Potential_NPF_ were spatially less uniform; increases were observed mostly in the eastern Arctic ( ~ 0.5 yr^−1^), whereas decreases (~ −0.5 yr^−1^) were observed mainly in the Barents Sea (Fig. 2e).

**Fig. 3:**
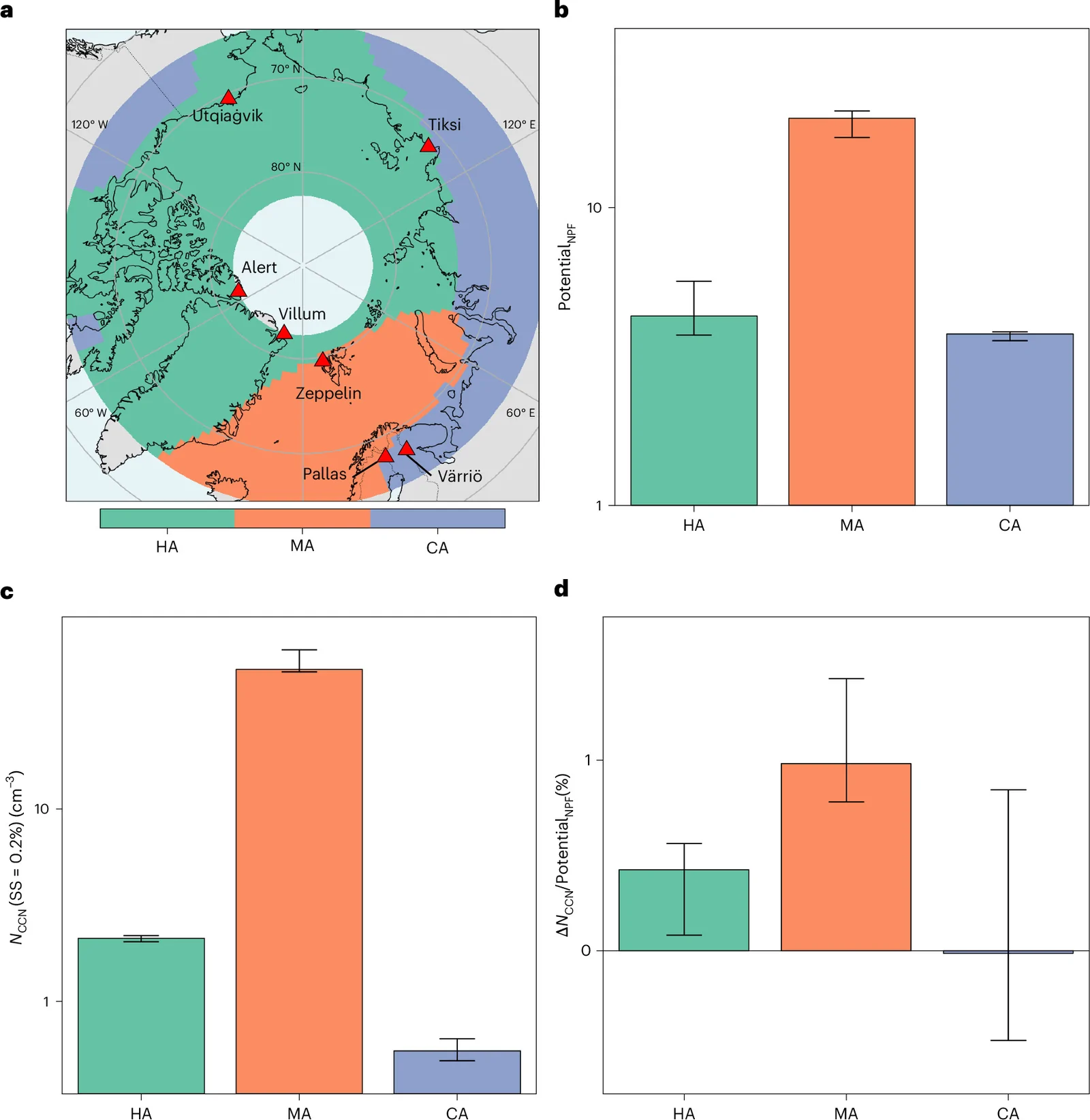
NPF potential and CCN trends of the various aerosol regimes. **a**, Map illustrating the spatial distribution of the three aerosol regimes: HA in green, MA in orange and CA in blue. Red triangles indicate locations of long-term Arctic monitoring sites. **b**–**d**, The bar plots represent area-weighted median values of selected variables from 2006 to 2021 during March–August. **b**, NPF potential (Potential_NPF_) as estimated from satellite-based proxies. **c**, CCN number concentration at supersaturation (SS) = 0.2% (*N*_CCN_) from clean continental and marine aerosols as obtained from Choudhury and Tesche^45^. Number concentration is obtained by vertically aggregating values from the ground level to boundary layer height that were obtained from ref. ^76^. **d**, The percentage change in *N*_CCN_ per unit increase in Potential_NPF_. The error lines in **b**–**d** represent the 95% bootstrap confidence interval around the area-weighted median as estimated from 10,000 bootstrap iterations. Basemap data in **a** from Natural Earth (https://www.naturalearthdata.com).

The Potential_NPF_ maps were overlaid atop a separately derived pan-Arctic dataset of CCN number concentration (*N*_CCN_)^45^ (Supplementary Fig. 3) and divided into aerosol regimes using a spatial clustering approach (Fig. 3a). Because only CCN from clean continental and marine aerosols were considered, the *N*_CCN_ values presented here could be considered representative of those from natural background conditions (more information of CCN retrievals in Supplementary Text 3). By accounting for the spatio-temporal patterns of both Potential_NPF_ and *N*_CCN_ simultaneously during the clustering process, different NPF–*N*_CCN_ dynamics could be identified. Three Arctic aerosol regimes were demarcated: the High Arctic (HA), Maritime Arctic (MA) and Continental Arctic (CA).

To understand the extent to which NPF affected CCN variability within each regime, the sign and magnitude of the *N*_CCN_–Potential_NPF_ slope provided useful indicators (Supplementary Fig. 4). A positive slope suggested that NPF activity and *N*_CCN_ were correlated to the same local processes or even linked via a direct NPF-to-CCN pathway, whereas a zero or negative slope indicated a greater influence of aerosol transport or sources uncorrelated to NPF. On this basis, despite differences in the magnitude of Potential_NPF_ levels, the High Arctic and Maritime Arctic both exhibited positive slopes (Fig. 3d), in contrast to the almost zero slope of the Continental Arctic.

**Fig. 4:**
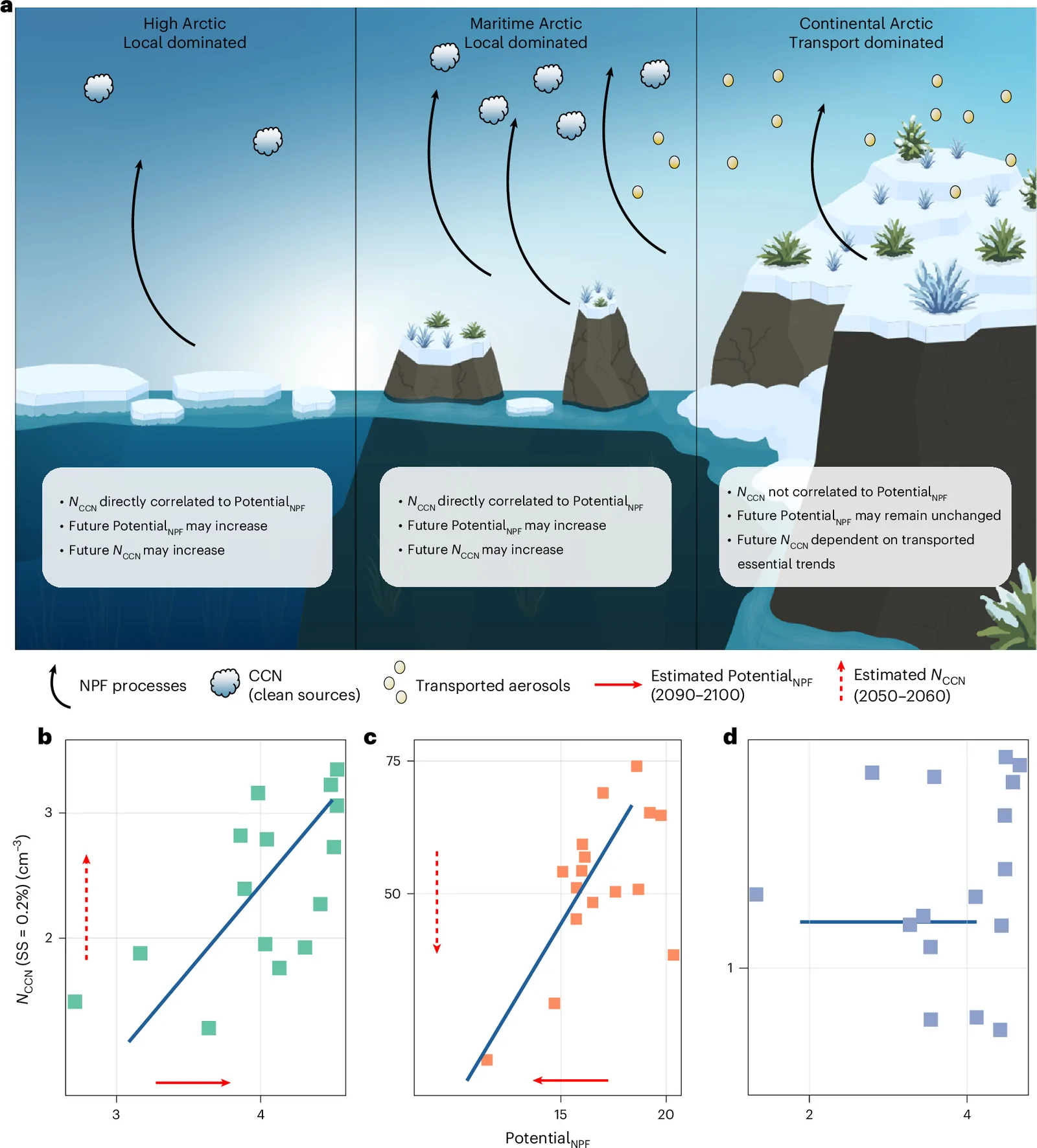
Contrasting aerosol–cloud feedbacks across the three Arctic aerosol regimes and their estimated changes. **a**, Conceptual figure of the NPF–CCN relationships in the three Arctic regimes: High Arctic, Maritime Arctic and Continental Arctic, and their future estimates. **b**–**d**, Scatter plots of *N*_CCN_ against Potential_NPF_ in the High Arctic (**b**), Maritime Arctic (**c**), Continental Arctic (**d**). Each point represents a yearly area-weighted median of March–August *N*_CCN_ and Potential_NPF_ values. To guide the eye towards the trend of the weighted scatters, blue lines were drawn in **b**–**d**. Red solid arrows indicate the estimated temperature-sensitivity trend of Potential_NPF_ by 2090–2100 following the temperature projections under the SSP2–4.5 scenario. Red dashed arrows indicate the speculated temperature-sensitivity trend of *N*_CCN_ by 2090–2100 under the SSP2–4.5 scenario.

In the High Arctic regime, with boundaries broadly following the summer sea-ice extent and snow-covered land areas, such as Greenland and the Canadian Archipelago, the average March–August Potential_NPF_ was low at 4.3 (95% confidence interval (CI): 3.7–5.6), consistent with the pristine ice- and snow-covered environment where local natural emissions and in situ NPF play a dominant role^27,42,46–48^ (Fig. 4a). Furthermore, the positive *N*_CCN_–Potential_NPF_ slope indicated that NPF activity and CCN concentrations were influenced by common local processes, corroborating numerous studies showing that local NPF contributed directly to the regional CCN budget^16,27,36,42,49^. Moving southwards into the Maritime Arctic, an area representing the open waters of the Barents and Norwegian seas, the average March–August Potential_NPF_ reached 20 (95% CI: 17–21), the highest of the three regimes. The positive *N*_CCN_–Potential_NPF_ slope similarly indicated that local NPF contributed to CCN production in this region, consistent with studies that showed surface processes such as pelagic biological activity or sea-ice formation/melt provide a range of precursor species that promote NPF activity^17,46,49,50^ (Fig. 4a).

In contrast, the transport-dominated Continental Arctic, spanning a broad belt from Finland to the Lena Basin and parts of the Canadian Arctic, had a Potential_NPF_ of 3.7 (95% CI: 3.6–3.8), comparable to that of the High Arctic but with a near-zero *N*_CCN_–Potential_NPF_ slope. Although terrestrial emissions are key sources of precursors for NPF^51,52^, the effectively flat *N*_CCN_–Potential_NPF_ relationship suggested that *N*_CCN_ concentrations there were probably sustained by transported accumulation-mode aerosols from lower latitudes, together with local anthropogenic emissions^28,29,53^ (Fig. 4a). Additionally, these accumulation-mode aerosols transported from lower latitudes, or originating from local anthropogenic emissions, would have increased the condensation sink, further suppressing Potential_NPF_.

## NPF potential and CCN under a warming Arctic

Given the strong correlations between air temperature and other environmental variables such as water vapour content or sea-ice concentration (Supplementary Fig. 5), changes in air temperature could thus act as a physical marker of Arctic warming. On the basis of the empirically derived temperature dependence of Potential_NPF_, the temperature-sensitivity estimate of the change in Potential_NPF_ was computed using the ensemble projected temperatures from the SSP2–4.5 intermediate emissions scenario. The changes in Potential_NPF_, following temperature changes by the mid-century (2050–2060; Fig. 2c) or end of century (2090–2100; Fig. 2f), showed spatial variation relative to the 2006–2021 baseline period. For reference, following temperature changes by 2090–2100, Potential_NPF_ could rise by 0.9 (95% CI: 0.7–1.9) in the High Arctic but decline by 6.6 (95% CI: 6.5–8.3) in the Maritime Arctic (Supplementary Table 3). The overall change in Potential_NPF_ in the Continental Arctic would be almost zero.

In the High Arctic, where temperatures remain below 0 °C for much of the year, the positive correlation between Potential_NPF_ and temperature (Supplementary Fig. 5) could be caused by enhanced precursor emissions through processes such as sea-ice melt at the marginal ice zone^49,54,55^, thereby raising Potential_NPF_. The observed increases (*p* < 0.1) in $\Delta{N}_{12.5-15\mathrm{nm}}$ at Alert and Villum during March–May and June–August in 2010–2019 suggested that the increase in Potential_NPF_ was already ongoing. Conversely, the declines in Potential_NPF_ in the Maritime Arctic could be brought about by a combination of factors such as changing meteorological patterns or shifting oceanographic processes^13,56,57^. On a process level, the reduction in Potential_NPF_ with higher temperatures could be due to increased volatility of precursor compounds^23^ or higher water vapour content, that, depending on the nucleation and growth mechanism, would decrease growth and/or formation rates^58,59^.

Of wider climatic importance is the response of *N*_CCN_ to these perturbations in the NPF dynamics. In the transport-dominated regimes of the Continental Arctic, future CCN levels would depend strongly on changes in long-range aerosol transport, which in turn would be influenced by factors such as wildfire occurrences and industrial emissions trajectories^53,60,61^. Whereas declining anthropogenic emissions in Europe and North America could reduce transported sulfate and organic aerosols, increases in boreal wildfire emissions^60,62^ and enhanced transport efficiency from mid-latitudes could offset or even reverse these declines^13,38,63,64^.

In the High Arctic, where CCN is strongly linked to local NPF, extrapolation of the *N*_CCN_–Potential_NPF_ slope observed during 2006–2021 indicated that *N*_CCN_ could increase by up to 11% (95% CI: 3–16%) by 2090–2100 with the expected temperature change under the SSP2–4.5 scenario (Fig. 4b). This High Arctic estimation is supported by several studies that had linked enhanced secondary aerosol production with sea-ice decline and phytoplankton blooms^14,15,49^. Given the low background *N*_CCN_ counts and the CCN-limited environment of this region, the estimated ~11% increase could substantially increase cloud droplet numbers, with implications for cloud properties^9,12,65^.

In contrast, the estimated change in *N*_CCN_ in the Maritime Arctic, using a similar extrapolation of the current *N*_CCN_–Potential_NPF_ relationship, showed as much as a 30% (95% CI: 15–31%) decline by 2090–2100 under SSP2–4.5, again emphasizing the contrast in this bellwether region (Fig. 4c). Unlike the other regimes, the Maritime Arctic lacked permanent monitoring stations; thus, observations here derived primarily from short-duration research cruises with limited temporal and spatial coverage, challenging our ability to accurately constrain Potential_NPF_ or *N*_CCN_ estimates. For instance, changing environmental conditions on land and at sea could favour or inhibit certain NPF pathways^16,66^, whereas shifts in anthropogenic emissions could alter the total precursor vapour and sink budget, thereby enhancing or suppressing NPF processes in the region^36,67^. Direct temperature-sensitivity estimates of *N*_CCN_ in the transport-dominated Continental Arctic regime were not attempted as future *N*_CCN_ would probably depend more on anthropogenic emissions than on local NPF potentials.

## Challenges in understanding Arctic aerosol processes

The distinct regional patterns identified in this study clarify why earlier studies, often focused on a single site, had produced seemingly different conclusions because no single site could be considered representative of the Arctic as a whole. Nevertheless, although this study is still not the final word in the Pan-Arctic narrative of aerosol–cloud interactions, we identified and, where possible, quantified some gaps regarding the importance of NPF for Arctic CCN. Our new methodologies produced a consistent set of NPF parameters from different sites and pan-Arctic maps of NPF potential from satellite proxies. This empirical understanding of the contribution of NPF to CCN in the Arctic can be used to evaluate model representations of aerosol processes and transport in the Arctic. In quantifying the relative importance of aerosol processes and transport across the Arctic, these results may help to identify structural model deficiencies associated with parameterization schemes and neglected processes in the region^68^.

Several challenges have limited the strength of our conclusions. The relatively coarse spatio-temporal resolution of the satellite retrievals prevented the Potential_NPF_ maps from capturing short-lived or local NPF events that occur in Arctic conditions^27,31^. Even for field measurements, the challenges of observing recently formed particles in the 1–12-nm range or obtaining direct gaseous precursor measurements hinder both process-level studies and differentiating between aerosol formation and early particle growth. Indeed, in the current Potential_NPF_ product, precursor emissions were not yet sufficiently parameterized; this limitation reflects the ongoing challenge in codifying the shifting balance between iodine-, sulfur- and VOC (volatile organic compounds)-driven NPF mechanisms in the Arctic^16,27,69,70^ and the roles of anthropogenic precursors such as sulfur species or VOCs^51,71–73^. Adding to the complexity, the current temperature dependence of Potential_NPF_ might also evolve as NPF-related emissions sources and the seasonality of their release change^14,16,74^, that is a reality already in the rapidly changing marginal ice zone of the High Arctic.

Therefore, addressing these knowledge gaps would require a network of long-term observations nested within larger-scale outputs (from satellites or models). In this study, by highlighting the broad characteristics of the High Arctic, Maritime Arctic or Continental Arctic, we emphasize the fundamental spatial heterogeneities among aerosol regimes across the Arctic and caution that simple narratives—such as ‘warming will increase NPF and CCN across the Arctic’—risk being misleading. Ergo, only by recognizing the Arctic as a mosaic of distinct and at times contrasting relationships between NPF and CCN can a more nuanced framework for projecting aerosol–cloud–climate feedbacks be articulated.

## Methods

### Observational datasets

Aerosol particle number size distributions were consolidated from various long-term observation sites (Alert, Pallas, Tiksi, Utqiaġvik, Villum, Värriö and Zeppelin) and research expeditions (Arctic Ocean 2018, MOSAIC and ARTofMELT) with their specific locations and data coverage shown in Fig. 1 and Supplementary Table 4. Measurements were collected using Differential Mobility Particle Sizers or Scanning Mobility Particle Sizers to obtain size-differentiated aerosol particle number concentrations. Further details of the instrument models used at each site are provided in Supplementary Table 4. The aerosol number size distributions were constrained to 12–500 nm to ensure comparability across observations. Site-specific data inspection and quality-control procedures were performed with further details on the specific procedures implemented at the individual observations documented in the corresponding references^25,26,28,29,31,32,48,72,77–80^. The final harmonized dataset comprised of a total of 21,705 valid data days across eight different Arctic locations spanning 2003–2024, although data coverage periods varied (Supplementary Table 4). Data from the research expeditions in the Arctic Ocean (Arctic Ocean 2018, MOSAIC, ARTofMELT) were grouped and classified as ‘Central Arctic’. From the particle number size distributions, number concentrations of nucleation mode particles (12.5–25 nm), Aitken-mode particles (25–100 nm) and accumulation-mode particles (100–500 nm) were computed.

### Quantifying NPF events and their relevant parameters

NPF-relevant parameters, as part of the data harmonization process, were computed using algorithmic objective methods^81–83^ to ensure inter-site comparability. The magnitude of daily fluctuations of particle number concentration within the 12.5–15 nm diameter range ($\Delta{N}_{12.5-15\mathrm{nm}}$) was computed and used as a proxy of atmospheric NPF, following approaches similar to those used in other observational atmospheric aerosol studies^61,84–86^. In greater detail, $\Delta{N}_{12.5-15\mathrm{nm}}$ is the difference in number concentration of particles in the 12.5–15-nm size bin between the daily peak and the median during the background hours, defined as the four consecutive hours with the lowest median ${N}_{12.5-15}$ in each month. In this manner, $\Delta{N}_{12.5-15\mathrm{nm}}$ captures the amplitude of the diurnal NPF signal while minimizing contamination from background variability. To enable inter-site comparison, *Z*-normalization was applied to $\log \Delta {N}_{12.5-15}$ values following equation (1a), with the resulting *Z*_score_ denoted as ${n}_{z}$. For each observational site, density histograms of ${n}_{z}$ were fitted with Kernel Density Estimator (KDE) curves, with five curves sufficiently reproducing the distribution at each site. Among these modes, only the aggregated normalized daily surface plots of particle number size distribution (${\rm{d}}N/\mathrm{dlog}{D}_{{\rm{p}}}$) for Mode 5, the mode with the highest central values, consistently exhibited the characteristic ‘NPF banana’ pattern associated with particle growth events (Supplementary Fig. 6). Accordingly, Mode 5 was visually identified as the ‘NPF mode’. This visual categorization was performed primarily to compare against historical approaches in which NPF event days were traditionally identified through manual classification^81,87^.

1a $${n}_{z}=\frac{x-{\mu }_{s}}{{\sigma }_{s}}$$

where $x=\log \left({\Delta N}_{12.5-15}\right)$, ${\mu }_{{\rm{s}}}$ is the station-specific mean, ${\sigma }_{{\rm{s}}}$ is the station-specific standard deviation

1b $${\mathrm{Potential}}_{\mathrm{NPF}}\left(x\right)={\int }_{-\infty }^{x}\frac{1}{\sigma \sqrt{2{\rm\pi }}}{e}^{\frac{-1}{2}{\left(\frac{t-\mu }{\sigma }\right)}^{2}}{\rm{d}}t$$

where $$x={n}_{{\rm{z}}}$$, μ=1.5, σ=0.56

For an objective approach to generalize this concept beyond the existing sites, a normal distribution was constructed and decomposed similarly using five KDE curves. The idea is that at any site with a sufficiently large number of measurements would have a distribution of ${n}_{{\rm{z}}}$ resembling a normal distribution, with its Mode 5 representing the ‘NPF mode’ as per the observation sites. Consequently, by evaluating the cumulative distribution function of this generalized Mode 5 at any ${n}_{{\rm{z}}}$ value (with the mathematically computed mean (*µ*) at 1.5 and standard deviation (*σ*) at 0.56 as in equation (1b)), a continuous range (0, 100) was obtained, which we define as Potential_NPF_. In other words, Potential_NPF_ is a metric in which values approaching 0 and 100 indicate extremely low and high potential for NPF activity, respectively. On the basis of the relationship observed at Värriö, monthly Potential_NPF_ values can be approximated as the percentage of days within that month during which NPF events are likely to occur (Supplementary Fig. 7c).

Thereafter, NPF-relevant parameters were calculated as follows. The condensation sink (CS), or the rate at which vapour molecules are removed from the atmosphere by condensation onto existing aerosol particles^88^, was calculated per equation (2):

2 $$\mathrm{CS}=2{\rm\pi }D\sum {\beta }_{{\rm{m}},{d}_{{\rm{p}}}}{d}_{{\rm{p}}}{N}_{{d}_{{\rm{p}}}}$$

where *D* is the diffusion coefficient of the condensing vapour, ${\beta }_{m,{d}_{p}}$ is the Fuchs–Sutugin transition regime correction factor, ${d}_{{\rm{p}}}$ is the particle diameter and ${N}_{{d}_{p}}$ is the number concentration of particles in the size bin with diameter ${d}_{{\rm{p}}}$.

Particle growth rates in the 15–20-nm size range (${\rm{GR}}$) and the formation rates of 15 nm particles (${J}_{15}$) were further computed from NPF event days. First, the daily surface plots of ${\rm{d}}N/\mathrm{dlog}\Delta{d}_{{\rm{p}}}$ values were normalized by scaling minimum and maximum values along each time bin from 0 to 1 so that growth events, even from ‘quiet’ NPF days^82^, can be visualized (Fig. 9 in Aliaga et al.^81^). Next, the recently developed maximum correlation method^83^ was applied to estimate ${\rm{GR}}$.

The particle formation rate ${J}_{{d}_{\rm{p}}}$ at a given diameter ${d}_{{\rm{p}}}$ was calculated as per equation (3):

3 $${J}_{{d}_{{\rm{p}}}}=\frac{{\rm{d}}{N}_{{d}_{{\rm{p}}}}}{{\rm{d}}t}+{\mathrm{CoagS}}_{{d}_{{\rm{p}}}}\times {N}_{{d}_{{\rm{p}}}}+\frac{\mathrm{GR}}{\Delta{d}_{{\rm{p}}}}\times {N}_{{d}_{{\rm{p}}}}$$

Equation (3) includes three components: the time derivative of the particle number concentration for the size range from ${d}_{p}$ to ${d}_{{\rm{p}}}+\Delta{d}_{{\rm{p}}}$, the coagulation sink ${\mathrm{CoagS}}_{{d}_{{\rm{p}}}}$ and the growth term that accounts for particles growing out of the size range. Correspondingly, the formation rates of 15 nm particles (${J}_{15}$) were calculated from the change in particle number concentration in the 15–20 nm range. More details regarding the data harmonization and NPF calculations are provided in Supplementary Text 1.

### Satellite-derived Potential_NPF_ maps

A satellite-based parametrization was developed to relate NPF potential, Potential_NPF_, to various satellite-derived environmental proxies. The dependent variable was ${n}_{{\rm{z}}}$ while six proxy predictors were included, namely, UV radiation (${\rm{UV}}$), surface air temperature (T), water vapour content ($[{\rm{H}}_2{\rm{O}}]$), atmospheric aerosol load (${\sigma }_{\mathrm{ext}}$), terrestrial precursor vapours ([NDVI]) using NDVI (Normalized Difference Vegetation Index) as a proxy and marine precursor vapours ($[{\rm{sea}}\,{\rm{ice}}]$) with sea-ice concentration used as a proxy, as expressed in equation (4). The choice of the predictors is further explained in Supplementary Text 2.

4 $$\overline{{n}_{{\rm{z}}}}=f\left(\mathrm{UV},T,\left[{{\rm{H}}}_{2}{\rm{O}}\right],{\sigma }_{\mathrm{ext}},\left[\mathrm{NDVI}\right],[\mathrm{sea}\,\mathrm{ice}]\right)$$

Potential_NPF_ was calculated from the derived ${n}_{{z}}$ values from equation (1b).

Data on surface UVB fluxes (UV) and precipitable water data ([H_2_O]) were obtained from the Clouds and the Earth’s Radiant Energy System (CERES) SYN1deg dataset^89–92^. Air temperature (*T*) data were obtained from monthly ERA5 records^76^. Aerosol extinction coefficient at 532 nm (${\sigma }_{\mathrm{ext}}$) was obtained from CALIPSO (Cloud–Aerosol Lidar and Infrared Pathfinder Satellite Observations) lidar profiles^93^. Extinction values were vertically integrated using the trapezoidal rule across the height bins from the surface up to 1,500 m above ground level. The NDVI dataset was obtained from the MODIS Version 6.1 vegetation indices product^94^; the sea-ice concentration data were obtained from the Global Sea Ice Concentration Climate Data Record produced by EUMETSAT (European Organisation for the Exploitation of Meteorological Satellites)^95^. To capture the influence of upwind precursor sources on a given location, the values of [NDVI] and [sea ice] at each pixel were computed as the spatial means across a 500-km buffer, approximating the fetch of transported precursors. These datasets were then resampled to monthly resolution and regridded to a 1° latitude × 2° longitude grid covering the polar region from 65° N to 83° N.

To parameterize equation (4), a machine-learning approach—the eXtreme Gradient Boosting (XGBoost) algorithm^96^—was trained using fivefold cross validation on collocated observational data from Zeppelin, Villum, Tiksi, Värriö, Utqiaġvik and from the Central Arctic cruise campaigns. Data from Pallas were withheld entirely from training and were used solely as an independent validation set. Hyperparameter optimization was performed over 500 candidate configurations, yielding a best cross-validated *R*^2^ of 0.84 (MAE (mean absolute error) = 0.18, *n* = 1836) on the training dataset. When evaluated against the held-out Pallas dataset, the algorithm achieved *R*^2^ = 0.67 (MAE = 0.32, *n* = 106), demonstrating good generalization to an unseen site. The derived values were then transformed into Potential_NPF_ maps using equation (1b). Additional information on creating the Potential_NPF_ maps is provided in Supplementary Text 2.

### CCN dataset

A height-resolved CCN dataset at 0.2% supersaturation level, calculated from the CALIOP lidar on board CALIPSO, was also acquired^45^. To focus only on the NPF-relevant CCN population, only CCN from clean continental and marine aerosols were considered. This observational CCN dataset was compiled following the workflow described in Choudhury and Tesche^97^ and has been validated with good accuracy against both measured data and modelled outputs^98,99^. For a given pixel, the CCN number concentration (*N*_CCN_) was obtained by vertically integrating the height layers from ground level to the boundary layer height. Boundary layer heights were obtained from monthly ERA5 data^76^. Supplementary Text 3 provides further discussion on *N*_CCN_ uncertainties.

### Trend and correlation analyses

Across all datasets, trends were calculated with the following approach. For the variables in the observational datasets, a Theil–Sen regression line was fitted to the time series of log_10_-transformed values for the period 2010–2019 to maintain a consistent timeframe for inter-site comparison. These trends, expressed as percentage change (of variable) per year (% yr^−1^), were calculated from equation (5).

5 $$\mathrm{Percentage}\,\mathrm{change}=\left({10}^{m}-1\right)\times 100 \%$$

*m* is the slope of the fitted Theil–Sen regression line.

Uncertainty ranges, when presented, were calculated from the 25th and 75th percentiles of the Theil–Sen slope distribution. Additionally, seasonal Mann–Kendall tests were used to check if the trends differ significantly from zero (no trend) at *p* < 0.1.

For the gridded datasets of Potential_NPF_ and *N*_CCN_, trend analyses were conducted for each pixel. In the case of Potential_NPF_, because Potential_NPF_ is already bounded within (0,100), its rate of change was not calculated from equation (5) but expressed directly as ${\Delta{\mathrm{Potential}}}_{\mathrm{NPF}}$ per year (yr^−1^). An additional metric—percentage change in *N*_CCN_ per unit change in Potential_NPF_ ($\Delta{N}_{\mathrm{CCN}}/{\mathrm{Potential}}_{\mathrm{NPF}}$ (%))—was computed to quantify their relationship during the March–August window. At each pixel, Theil–Sen regression lines were fitted to a scatter of ${\mathrm{log}}_{10}({N}_{\mathrm{CCN}})$ against Potential_NPF_, with the resulting slope converted to percentage change using equation (5). Thus, $\Delta{N}_{\mathrm{CCN}}/{\mathrm{Potential}}_{\mathrm{NPF}}$ represented the percentage change in *N*_CCN_ per 1-unit increase in Potential_NPF_.

The gridded datasets of Potential_NPF_ and *N*_CCN_ were used to classify the polar region into three regimes (Fig. 3) using a Spatial Gaussian Mixture Model—an unsupervised soft clustering approach. Regime-level statistics were then calculated using the area-weighted median of all pixels within each regime. Uncertainty ranges were obtained by bootstrapping the weighted median calculation 10,000 times, with the 95% confidence intervals of the bootstrapped distribution reported.

### The effects of temperature changes

To calculate future temperature-sensitivity estimates, surface air temperatures for the SSP2–4.5 (intermediate emissions) scenario were obtained from five CMIP6 (Coupled Model Intercomparison Project Phase 6) models—CESM2^100^, EC-Earth3-CC^101^, MIROC-ES2L^102^, NorESM2-LM^103^ and UKESM1-0-LL^104^—and averaged to produce an ensemble mean.

To identify the relationship between temperature (*T*) and Potential_NPF_ during the March–August period, a three-step piecewise function was applied at each pixel. At each pixel, three temperature-dependent slopes ($s\left(T\right)$) were computed by Theil–Sen regression of ${\mathrm{log}}_{10}({\mathrm{Potential}}_{\mathrm{NPF}})$ values against *T* values within each temperature bin: below 0 °C, 0–10 °C and above 10 °C (equation (6a)). These slopes were integrated from a baseline temperature (*T*_*i*_)—defined as the median temperatures during 2006–2021—to a projected temperature (*T*_pr__oj_) to output the corresponding temperature-sensitivity ${{\mathrm{Potential}}_{\mathrm{NPF}}}_{\mathrm{Proj}}$ (equation (6b)).

6a $$s\left(T\right)=\left\{\begin{array}{cc}{s}_{\rm{low}}, & T < 0\\ {s}_{\rm{med}}, & 0\le T < 10\\ {s}_{\rm{high}}, & T\ge 10\end{array}\right.$$

6b $${{\mathrm{Potential}}_{\mathrm{NPF}}}_{\mathrm{Proj}}={{\mathrm{Potential}}_{\mathrm{NPF}}}_{i}\times {10}^{{\int }_{{T}_{\mathrm{proj}}}^{{T}_{i}}s(u){\rm{d}}u}$$

Supplementary Text 4 provides further discussion on this temperature parameterization.

## Online content

Any methods, additional references, Nature Portfolio reporting summaries, source data, extended data, supplementary information, acknowledgements, peer review information; details of author contributions and competing interests; and statements of data and code availability are available at https://doi.org/10.1038/s41561-026-02114-x.

## Supplementary information

Supplementary Information Supplementary Texts 1–4, Figs. 1–10 and Tables 1–5.

Peer Review File

## Data Availability

CERES data are available at https://ceres.larc.nasa.gov/data/ (ref. ^89^); CALIOP data are available at https://asdc.larc.nasa.gov/project/CALIPSO (ref. ^93^); temperature, sea-ice concentration and BLH datasets are available at https://cds.climate.copernicus.eu/datasets (refs. ^95,105^); the NDVI dataset is available at https://doi.org/10.5067/MODIS/MOD13A3.061 (ref. ^94^). Global CCN number concentration data are available at https://doi.org/10.1594/PANGAEA.956215 (ref. ^45^). The Potential_NPF_ maps generated and used in this study are available via Zenodo at https://doi.org/10.5281/zenodo.22127460 (ref. ^106^).

## Code availability

Relevant code used in this study is available via Zenodo at https://doi.org/10.5281/zenodo.22127460 (ref. ^106^).
