# Supplementary material for: Contrasting aerosol–cloud feedbacks across a warming Arctic
Source: Nat Geosci. 2026 Sep 28;19(11):1292–300. doi: 10.1038/s41561-026-02114-x (PMC13739744; doi:10.1038/s41561-026-02114-x)
Supplement: Supplementary file 1 [file 41561_2026_2114_MOESM1_ESM.pdf]

nature geoscience

Supplementary information https://doi.org/10.1038/s41561-026-02114-x

Contrasting aerosol–cloud feedbacks across a warming Arctic

In the format provided by the authors and unedited

Supplementary Information for

Contrasting aerosol-cloud feedbacks across a warming Arctic

Corresponding authors:
Samuel De Xun Chua (samuel.chua@helsinki.fi);
Katrianne Lehtipalo (katrianne.lehtipalo@helsinki.fi)

Contents of this file

Text S1 to S4
Figures S1 to S10
Table S1 to S5
Supplementary References

Supplementary Text S1: Quantifying NPF events and potential

Preliminary data curation was performed so that the particle size number distribution (PNSD) datasets from the various stations would be comparable. Although some sites had particle size number distribution (PNSD) extending down to 3 nm-sized particles, the lower cutoff for our harmonized dataset was chosen to be approximately 12 nm, as that was the limit where there was quality-controlled data available across all measurement sites. For Villum, data during 2019-2020 were limited to <360 nm due to instrument limitations. Specific anomaly-detection and calibration procedures applied at each site were documented in the corresponding station references listed in SI Table S4. All in all, the final harmonized dataset comprised a total of 21705 valid data days, although data coverage periods varied (SI Table S4).

Compared to traditional methods that could be subjective and/or time-consuming [1,2], a procedure to objectively and quickly quantify NPF events was applied in this study to ensure inter-site comparability. Given the constraint of comparable data for only particles > 12nm and expanding on the idea that daily fluctuations of nucleation-mode particles can be indicators of NPF events [3,4], the magnitude of diurnal fluctuations of particle number concentration within the 12.5-15 nm diameter range ($\Delta N_{12.5-15\,nm}$) was thus computed and used as a proxy of atmospheric NPF.

Since Arctic NPF events mirror the diurnal solar radiation cycle even during the summer months [5,6], there would be an active time window for NPF, often during or shortly after the peak sunlit hours, when there would be a rise in $N_{12.5-15\,nm}$ that corresponds to NPF activity. Thus, to ensure that $N_{12.5-15\,nm}$ only captured the peak due to NPF activity, $N_{12.5-15\,nm}$ was taken to be the difference between the daily peak $N_{12.5-15\,nm}$ values and the median $N_{12.5-15\,nm}$ value during the background hours. The background hours, often during the night, were identified by the four consecutive hours with the lowest median $N_{12.5-15\,nm}$ in each month. The plot of formation rates of 15 nm-particles (J_{15}) against $N_{12.5-15\,nm}$ showed that J_{15} increased with increasing $\Delta N_{12.5-15}$ (SI Figure S7a), further justifying $\Delta N_{12.5-15}$ as a suitable proxy for NPF intensity.

To enable inter-site comparison, Z-normalization was applied to $\log \Delta N_{12.5-15}$ values following Eq. 1a, with the resulting Z_{score} denoted as n_z. Density histograms of n_z were fitted with Kernel Density Estimator (KDE) curves, and five curves were sufficient to reasonably reproduce the distribution at each site (SI Figure S6). Clear 'banana'-shaped events (Class I under the classification scheme by Dal Maso et al. (2005)) were observed in Mode 5 (SI Figure S6)—the KDE mode with the highest central value—while the other modes showed little or no signs of particle growth (belonging to the undefined category under the classification scheme by Dal Maso et al. (2005)).

In a general situation with a sufficiently large number of datapoints, n_z follows a normal distribution centered at 0 (SI Figure S8a). Similarly, this distribution could be decomposed into five KDE curves, with Mode 5 representing the 'NPF mode'. Using this normal distribution, $Potential_{NPF}$ would be the corresponding output of the cumulative distribution function of the NPF mode for a given n_z (Eq. 1b). The calculated Gaussian parameters for the NPF mode of the general distribution were $\mu = 1.5$ and $\sigma = 0.56$, consistent with those derived from the monitoring stations ($\mu \approx 1.2$–1.6, $\sigma \approx 0.37$–0.50) (SI Figure S8b).

In contrast to the Bayesian probabilistic approach outlined above, a common technique for estimating 'NPF probability' would be to divide the number of identified 'NPF days' by the entire dataset – a frequentist probabilistic approach. However, this approach is reliant on the period of the dataset; for

example, a dataset collected only during the summer NPF season will yield a higher NPF occurrence percentage than another dataset collected year-round. Therefore, given the Arctic's unique situation of sparsely located sites and irregular monitoring periods due to the harsh winters, the Bayesian probabilistic approach used here could be less subjective than the said conventional frequentist probabilistic method.

Importantly, $Potential_{NPF}$ should be interpreted as a probabilistic metric that reflects the position of the observed n_z value within the NPF-mode distribution. Despite this probabilistic formulation, $Potential_{NPF}$, when compared to formation rates of 15 nm-particles (J_{15}), showed that $Potential_{NPF}$ increased with increasing J_{15} (SI Figure S7b), justifying $Potential_{NPF}$ as an indicator for NPF intensity. At Värriö, the longest dataset in our records, monthly values of $Potential_{NPF}$ also correlated well with the monthly percentage of days with Class I or Class II events (classified manually according to Dal Maso et al. (2005)) in a 1:1 relationship (SI Figure S7c). Therefore, an approximation could be made, with care, that the monthly value of $Potential_{NPF}$ roughly corresponded to the percentage of days within that month during which NPF events are likely to occur.

Supplementary Text S2: Estimating NPF potential from satellites

In principle, nucleation mode particle concentration should be linked to environmental variables of UVB radiation, precursor gas concentrations and condensation sink [7,8]. In practice, however, the challenges of obtaining accurate satellite-based proxies for these parameters, combined with their nonlinear relationships with nucleation mode aerosols, had limited progress in developing reliable satellite-based mapping. Notwithstanding, NPF intensity, sometimes expressed through formation rate, had been parametrized using auxiliary variables such as global radiation, precursor gas concentration, relative humidity and condensation sink [9–11]. Given the Arctic setting of this study, the terrestrial precursor vapours are likely dominated by highly oxygenated organic molecules (HOMs) associated with terrestrial biogenic emissions[12,13], while marine precursor vapours are likely composed of iodine- and sulfur-containing compounds linked to open waters and processes associated with sea-ice formation and breakup [5,12,14–19]. Since these precursor vapours could not be reliably observed from satellite measurements, indicators of their source regions, namely NDVI and sea-ice concentration, were thereby used as proxies for their abundance.

Regarding the satellite-derived variables as shown in Eq. 4, Surface UVB fluxes (*UV*) were obtained from the Clouds and the Earth's Radiant Energy System (CERES) SYN1deg dataset, which provided harmonized spectral irradiance derived from CERES scanning radiometers aboard multiple satellite platforms, including MODIS Terra, MODIS Aqua, and Suomi NPP [20,21]. From the same CERES data collection, precipitable water data ($[H_2O]$), here used as an indicator of water vapour, were also obtained. These values were derived from the Goddard Earth Observing System (GEOS) version 5.4.1, and constrained by both ground-based and satellite observations [22,23]. As the CERES $[H_2O]$ dataset was not height resolved, with output similar to that from a microwave radiometer, a high $[H_2O]$ value could indicate high specific humidity and/or the presence of clouds.

As a proxy for atmospheric aerosol load, the aerosol extinction coefficient at 532 nm (σ_{ext}) was obtained from CALIPSO (Cloud-Aerosol Lidar and Infrared Pathfinder Satellite Observations) lidar profiles [24]. Humidity may influence the spaceborne retrieval of σ_{ext} [25], leading to divergence from ground-based measurements of aerosol optical properties. In this study, the coupling of $[H_2O]$ and σ_{ext} within the model could address, at least partially, the effects of humidity on aerosol optical properties. The σ_{ext} values were vertically integrated using the trapezoidal rule across the height bins from the

surface up to 1500 m above ground level to approximate the total aerosol loading in the lower troposphere. Data required to calculate surface topography were derived from the Global Multi-resolution Terrain Elevation Data 2010 (GMTED2010) digital elevation model dataset [26].

The NDVI dataset, derived from multispectral sensors onboard Terra, was obtained from the MODIS Version 6.1 vegetation indices product [27]. Sea ice concentration, defined as the fraction of the gridded product covered by sea ice, was obtained from the EUMETSAT Ocean and Sea Ice Satellite Application Facility (OSI SAF) global sea ice concentration climate data record. This sea ice dataset was derived from brightness temperatures measured by the Special Sensor Microwave Imager/Sounder [28].

Besides XGBoost, several alternative calibration algorithms were evaluated to derive the $Potential_{NPF}$ parameterisation; however, XGBoost demonstrated the best overall performance. Other regression approaches trained on the full dataset, including multiple linear regression, ridge regression, and Lasso regression, yielded R^2 values of 0.31, 0.39, and 0.43, respectively, much lower than the R^2 of 0.84 achieved by the XGBoost model on the training dataset (SI Figure S9a). The testing datasets, which were the hold-out predictions from the five-fold cross-validation, achieved good performance with a mean R^2 of 0.65 (SI Figure S9b). The comparable R^2 values for the unseen dataset of Pallas (R^2 = 0.67) justified that the XGBoost algorithm had not overfitted the training data and remained generalisable to an unseen location. Slight negative biases in n_z were observed in the testing datasets (-0.05), meaning that n_z were systematically underestimated. Consequently, estimations of $Potential_{NPF}$ would likely be lower than those of actual conditions.

To assess the influence of the independent variables in Eq. 4, the SHapely Additive exPlanations (SHAP) framework was used [29]. The absolute SHAP values provided a measure of the relative importance of each predictor to the target variable, with larger absolute values indicating greater influence. Based on this SHAP analysis (SI Figure S9c), the predictors ranked in order of importance were: UV radiation (UV), water vapour content ($[H_2O]$), atmospheric aerosol load (σ_{ext}), sea ice concentration ($[sea\ ice]$), NDVI ($[NDVI]$) and air temperature (T).

However, a limitation of the machine learning model was that the cruise-based observations (Arctic Ocean 2018, MOSAiC, ARTofMELT) were much shorter in duration than the records at the long-term terrestrial stations so the estimated $Potential_{NPF}$ values might not have been representative of Maritime Arctic conditions year-round. In particular, the MOSAiC expedition occurred during an extreme positive Arctic Oscillation year (2019–2020), which coincided with anomalously high wintertime accumulation-mode concentrations [30]. Therefore the $Potential_{NPF}$ values over the open ocean would benefit from dedicated long-term measurements to better constrain them.

<u>Supplementary Text S3: CCN number concentration</u>

Although model-based estimates for global CCN number concentration exist, matching the simulated results to field observations had proved challenging, due to the incomplete representation of aerosol chemistry and/or physics [31,32]. In this study, we used the CALIPSO-derived CCN dataset from Choudhury & Tesche[33] because of its good reported agreement with ground observations (correlation coefficient ~0.7) [34] that made it one of the better observationally constrained CCN dataset at the point of writing.

This dataset likely underestimated absolute N_{CCN} values in the Arctic due to the following reasons. Within the Arctic, CCN-capable particles can be as small as 30 nm [35], near the detection limits of satellite lidar; thus, a substantial fraction of CCN-sized particles was likely not retrieved. Also, the

choice of supersaturation ratio (SS) added another area of uncertainty. In-situ observations from the Arctic showed a wide range of SS from ~0.11 to 1 [36–41]. In this study, the use of SS = 0.2%, at the lower range of observed values, could thus lead to further undercounting of CCN-capable particles.

The reliability of our estimates further depended on accurately identifying the planetary boundary layer (PBL), where cloud processing occurs. In theory, N_{CCN} should be homogeneous across the mixing layer; however, CALIPSO extinction profiles in the Arctic showed declining aerosol concentrations with height even within the boundary layer [42]. This observation could reflect misidentification of the PBL in reanalysis products such as ERA5 [43], that would again underestimate the CALIPSO-derived N_{CCN} values with respect to surface measurements.

To assess the scale of the underestimation, N_{CCN} (SS =0.2%) data from long-term field observations at Zeppelin [44] were compared to collocated CALIPSO-derived CCN number concentration from all aerosol types, here denoted as $N_{CCN\ all}$ (SI Table S5). The observations at Zeppelin were the only set of quality-controlled measurements that had the same supersaturation ratio and similar seasonal coverage to the CALIPSO-derived $N_{CCN\ all}$. Median values for the field measurements were 80 cm^{-3} (IQR: 71–89 cm^{-3}) while that of the CALIPSO-derived dataset was lower at 54 cm^{-3} (IQR: 44–69 cm^{-3}). Thus, the underestimation of the CALIPSO-derived $N_{CCN\ all}$ as compared to field values could be ~1.5-2× lower.

Using the SS-N_{CCN} correlation parameterised at Zeppelin [37], N_{CCN} at SS=1.0 could be 1.5-2× higher than that at SS=0.2. Estimating N_{CCN} values at other SS levels can be done by using suitable scaling parameters – a procedure that has been described in multiple studies [37,41,45–47]. Altogether, considering the various uncertainties in using spaceborne sensors vis-à-vis ground-based measurements, the CALIPSO-derived $N_{CCN\ all}$ values in this study could be 1.5-4× lower than the actual activated N_{CCN} values. Importantly, the N_{CCN} data presented in this study (unless specifically stated) only presented CCN from clean continental and marine aerosols, thereby excluding other CCN types such as polluted continental and elevated smoke. Thus, the N_{CCN} values presented in this study cannot be taken as the total CCN population.

Given this level of uncertainty, the absolute values of N_{CCN} presented in this study could be interpreted as the lower limit of true ground conditions. However, the arguments based on the $\Delta N_{CCn}/Potential_{NPF}$ metric remained valid as it reflected relative changes; the systematic underestimation in absolute CCN values cancelled out mathematically.

Supplementary Text S4: Temperature parameterization

Since empirical observations of temperature (T) and $Potential_{NPF}$ indicated that they are related non-linearly (SI Figure S5a, b, c), the temperature-$Potential_{NPF}$ relationship was expressed with a piecewise function (Eq.6a), recognizing the differences in processes below 0°C, in the transition range of 0-10°C, and in the warmer range above 10°C. As illustrated in SI Figure S5, at temperatures below 0°C, $Potential_{NPF}$ generally increased with rising temperature; however, as temperatures increased above 0°C, the positively correlated trend was only preserved in some regions.

Since temperature change is a driving force or an indicator in many Arctic processes [48], temperature change was regarded as a proxy for environmental changes in the Arctic. Among the six predictors in the XGBoost model, surface air temperature was also the most reliably projected CMIP6 variable in the Arctic, whereas future projections of the other variables carried larger inter-model uncertainty. Furthermore, temperature, consistent with its role as a driver of Arctic atmospheric and surface

conditions, was found to be strongly correlated with other environmental indicators such as water vapour, sea ice and terrestrial vegetation (SI Figure S5d, e, f), thereby justifying the use of temperature changes as a good indicator of Arctic warming. The temperature sensitivity analyses in this study were solely built upon existing observed temperature-$Potential_{NPF}$ relationship, implying the necessary assumption that the historical temperature dependence of $Potential_{NPF}$ would persist into the future. This assumption is by no means certain, especially in regions experiencing rapid Arctic amplifications. Therefore, the temperature-sensitivity estimates presented here should be understood as quantifying the $Potential_{NPF}$ response to warming alone, with changes in the remaining predictors representing additional sources of uncertainty not yet quantified.

Given the known cold biases in historical Arctic temperatures simulated by many CMIP6 models [49], we would expect a corresponding cold bias in the projected temperatures used in our analysis. Consequently, the projected warming by 2050–2060 might be underestimated. As our $Potential_{NPF}$ and N_{CCN} estimates were based on empirically derived temperature sensitivities, any underestimation of projected warming would translate into lower-magnitude projected changes in both $Potential_{NPF}$ and N_{CCN}. Therefore, our temperature-sensitivity estimates should be interpreted as conservative estimates rather than upper-bound projections (SI Table S3).

For the temperature-sensitivity estimates, two uncertainty scales were presented (SI Table S3). The first set of "variability" ranges represented the upper and lower quantiles of possible outcomes, considering the model ensemble variability of Arctic warming estimates, $Potential_{NPF}$ estimates, and the fitted temperature-$Potential_{NPF}$ parameterizations (SI Figure S10). For the regional-level analyses presented in the main text, the set of "confidence" ranges was used. This "confidence" range expressed confidence in the area-weighted regional median values, obtained by bootstrapping the procedure 10 000 times and reporting the 95 % confidence interval.

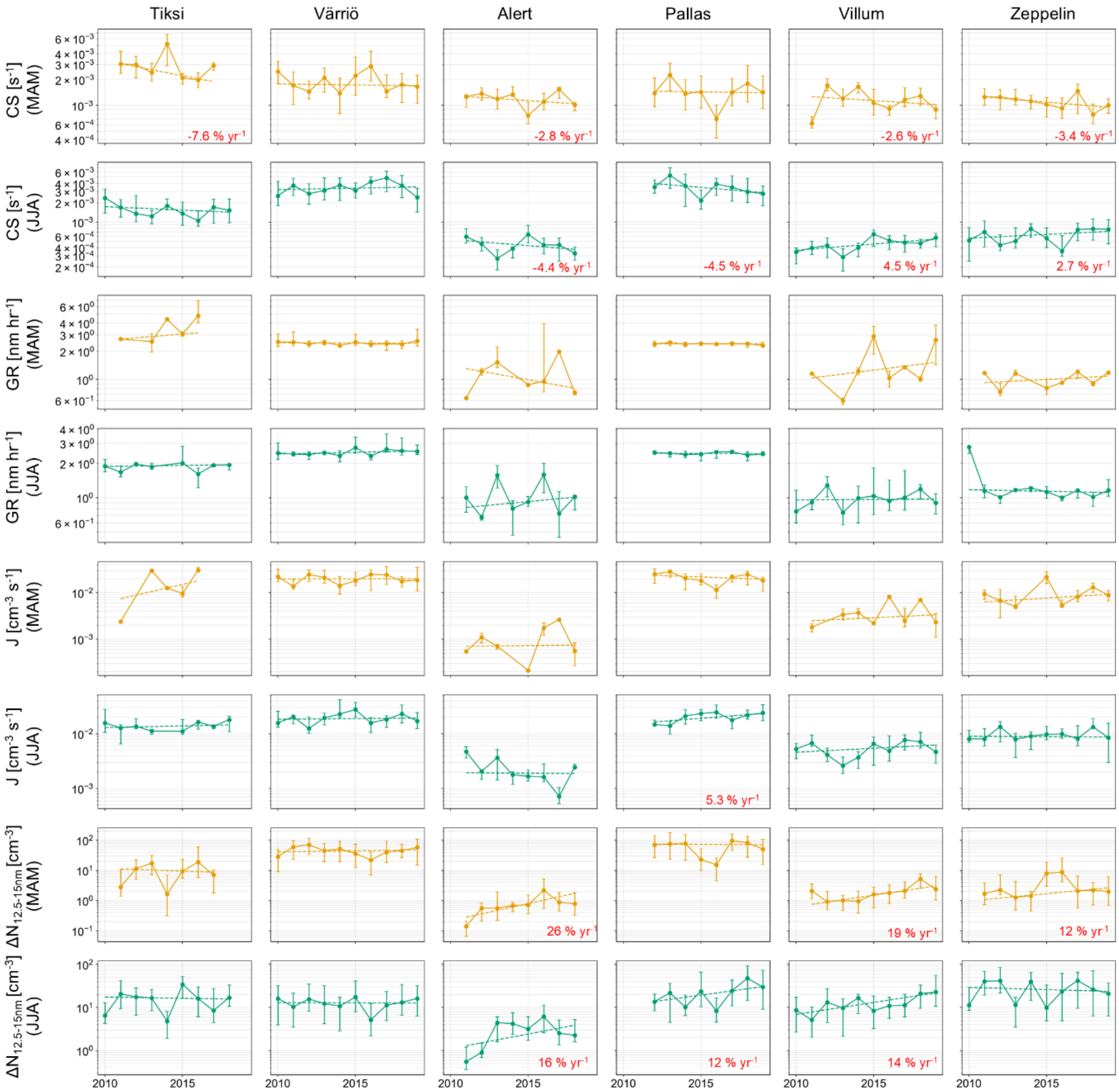

Figure S1. Timeseries of selected aerosol parameters from the common window of 2010-2019 separated by seasons (MAM: March- May, JJA: June-August) with the median and interquartile range at each yearly value shown. Trends were calculated from fitted Theil-Sen trend lines. Slopes that were statistically significant (Mann-Kendall test, $p<0.1$) were expressed as percentage change/year according to Eq. 5 and printed in red in the subplots. Parameters are CS: condensation sink; GR: growth rate at 15-20 nm; J: formation rate at 15 nm; and $\Delta N_{12.5\text{-}15\ nm}$: diurnal variation in 12.5-15 nm particle concentration.

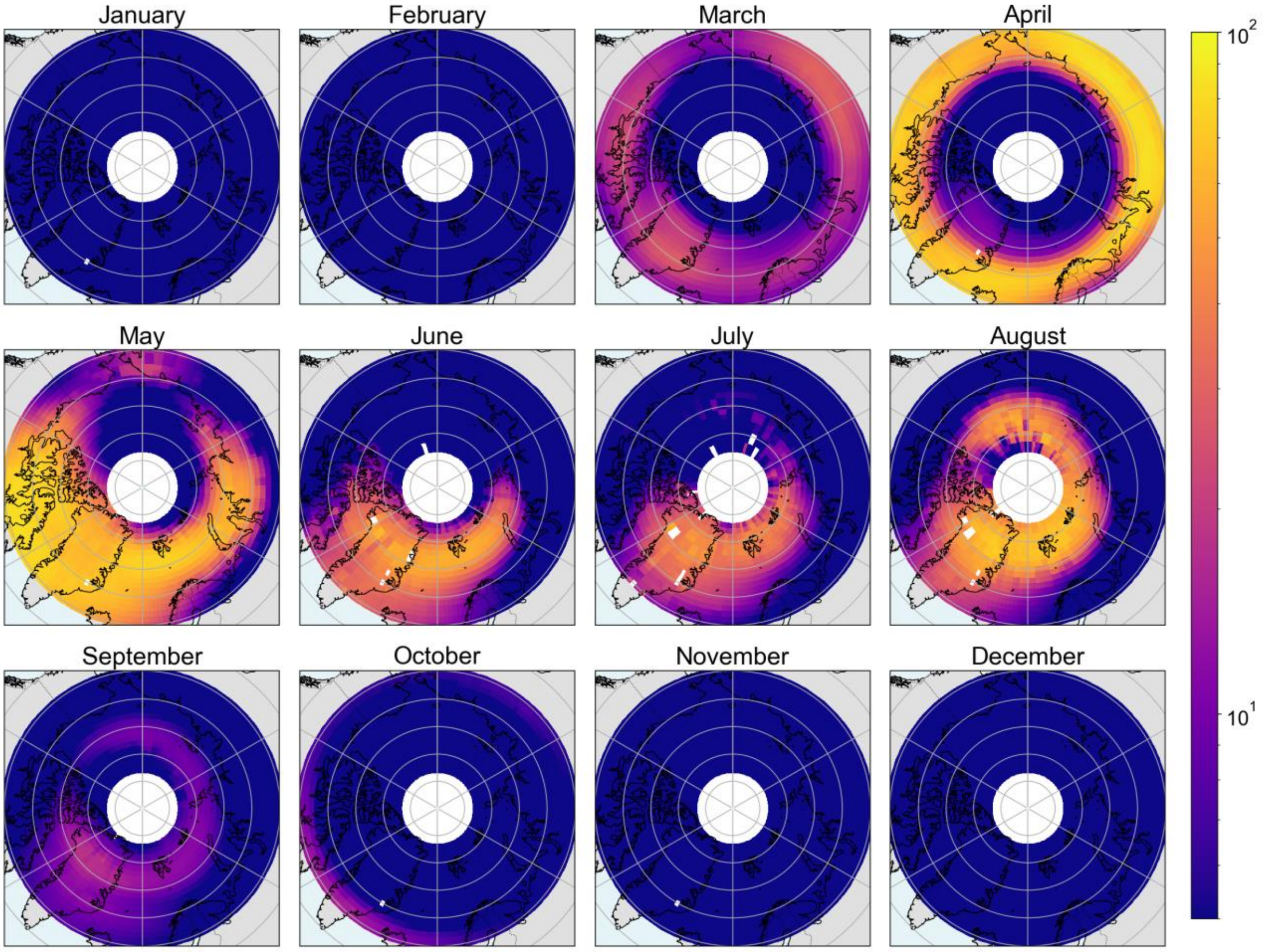

Figure S2. Median monthly maps of NPF potential ($Potential_{NPF}$) averaged over 2006-2021 as estimated from satellite-based proxies. $Potential_{NPF}$ ranged (0,100) with 0 and 100 representing infinitely low and high potential of NPF events occurring respectively

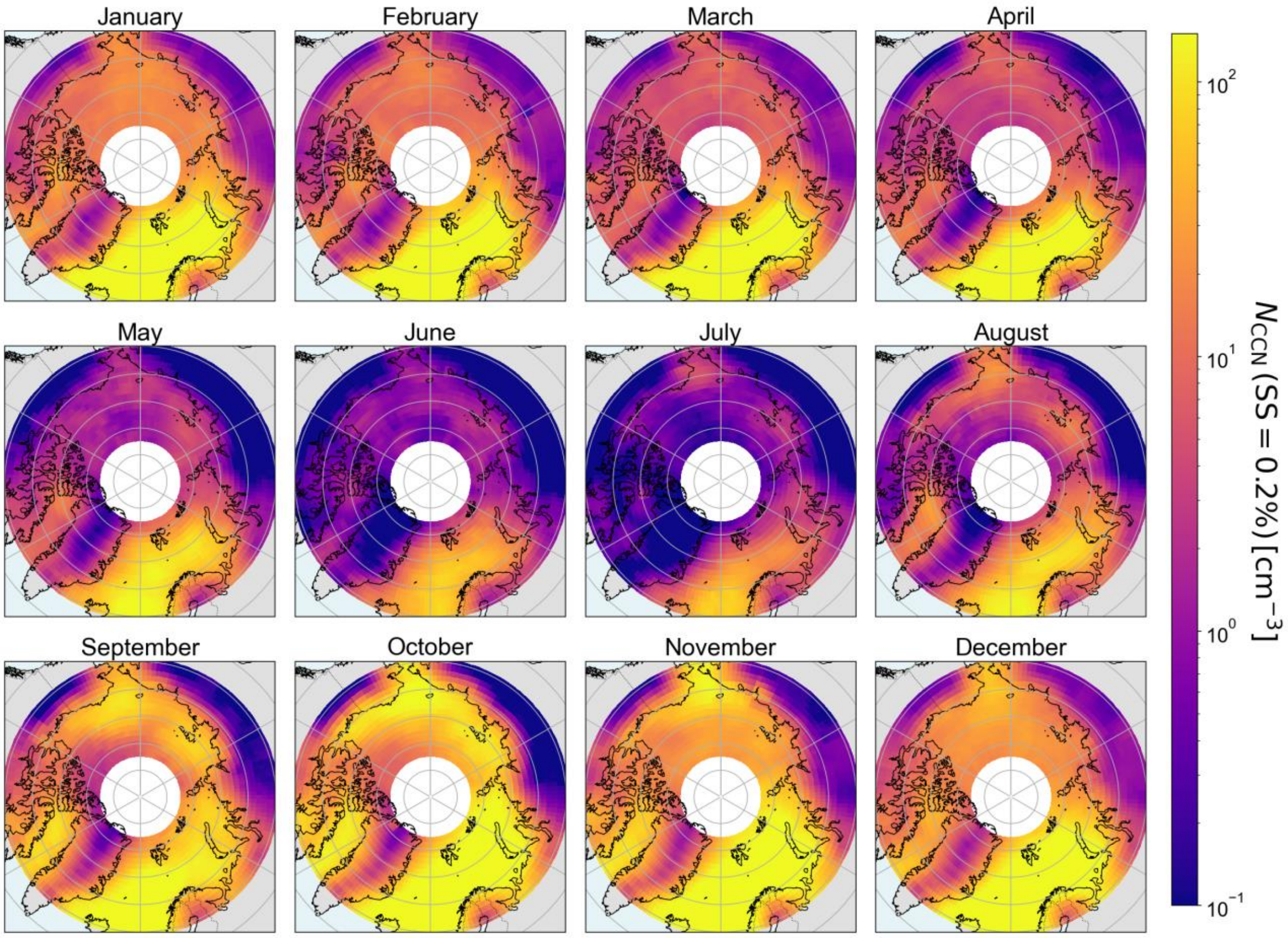

Figure S3. Median monthly maps of CCN (SS=0.2%) number concentration from clean continental and marine aerosols averaged over 2006-2021. Dataset was obtained from Choudhury & Tesche [33]. Number concentrations were obtained by vertically aggregating values from the ground level to boundary layer height, that were obtained from ERA5[50].

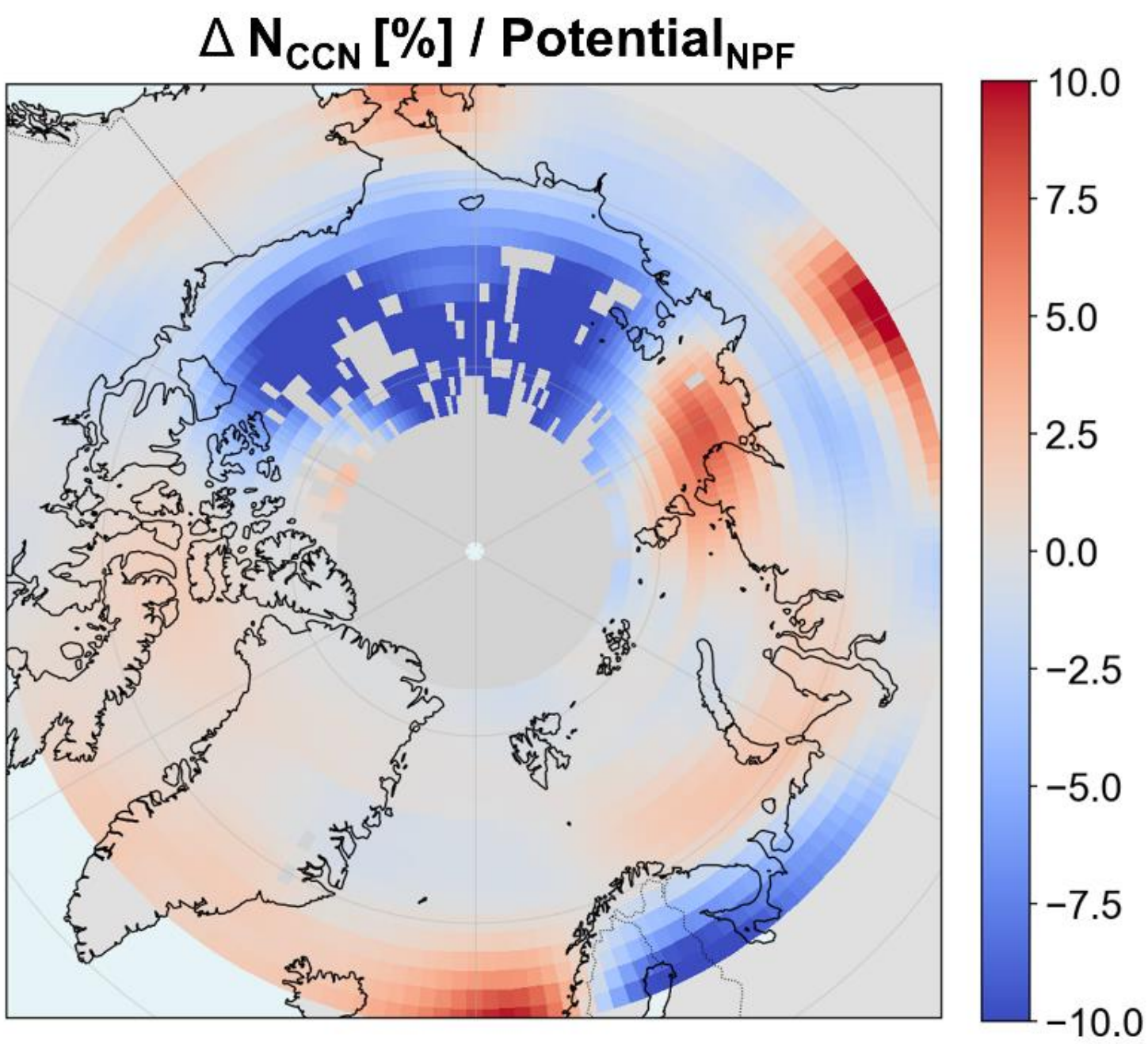

Figure S4. The percentage change in CCN number concentration per 1 unit increase in $Potential_{NPF}$ calculated at each pixel as observed during March-August in 2006-2021.

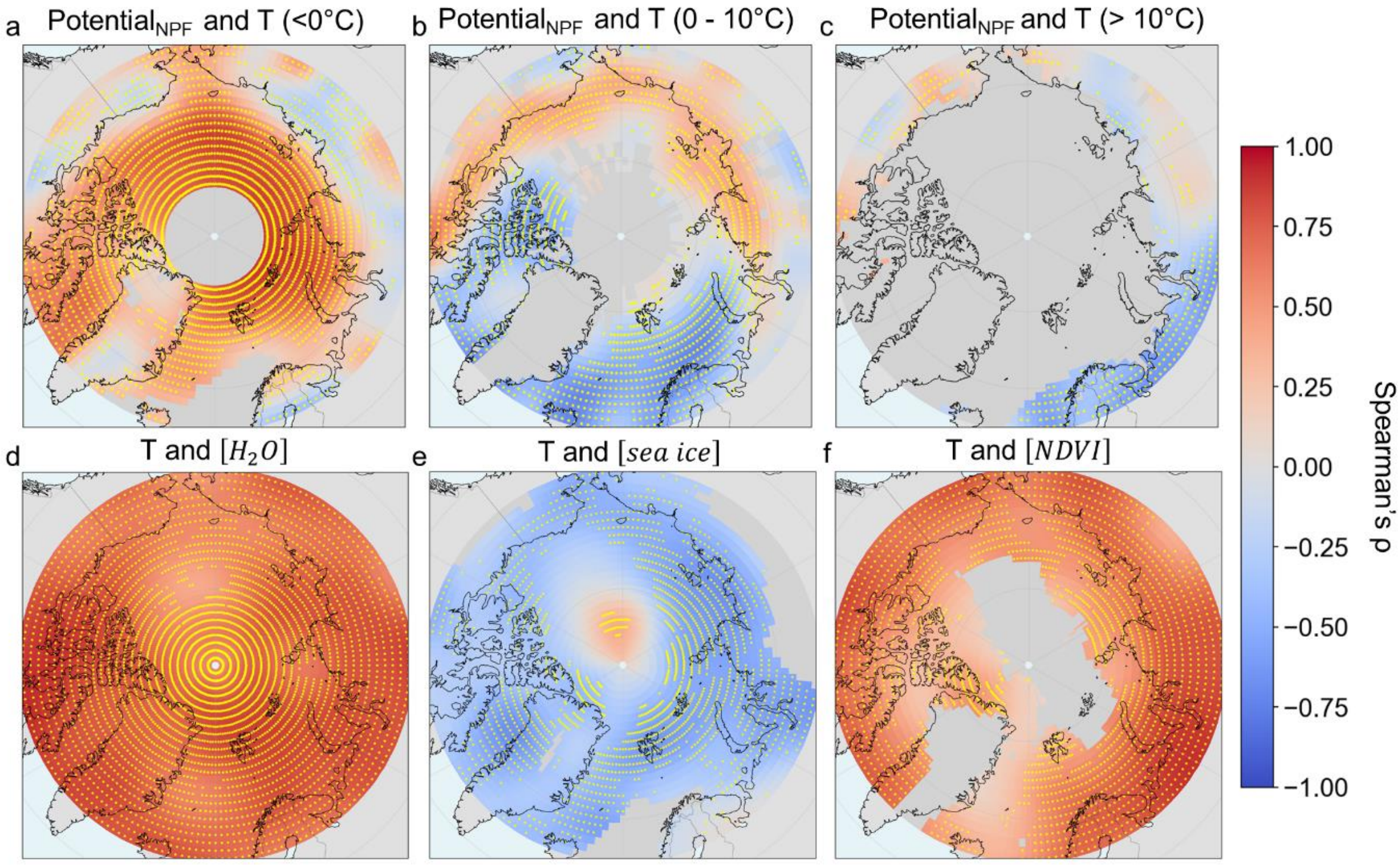

Figure S5. Spearman's ρ values between surface air temperature (T) and $Potential_{NPF}$ calculated at each pixel for data during 2006-2021 March-August at three temperature bins: (a) <0 °C, (b) 0-10 °C, and (c) >10 °C. For visualisation of the sensitivity of the satellite variables to T, Spearman's ρ values were calculated at each pixel for data during 2006-2021 March-August between surface air temperature and (d) water vapour content ($[H_2O]$), (e) sea ice concentration ($[sea\ ice]$), and (f) Normalised Difference Vegetation Index ($[NDVI]$). The values of $[NDVI]$ and $[sea\ ice]$ at each pixel were computed as the spatial mean across a 500 km buffer. Pixels where ρ values were statistically significant ($p < 0.1$) were marked by yellow dots.

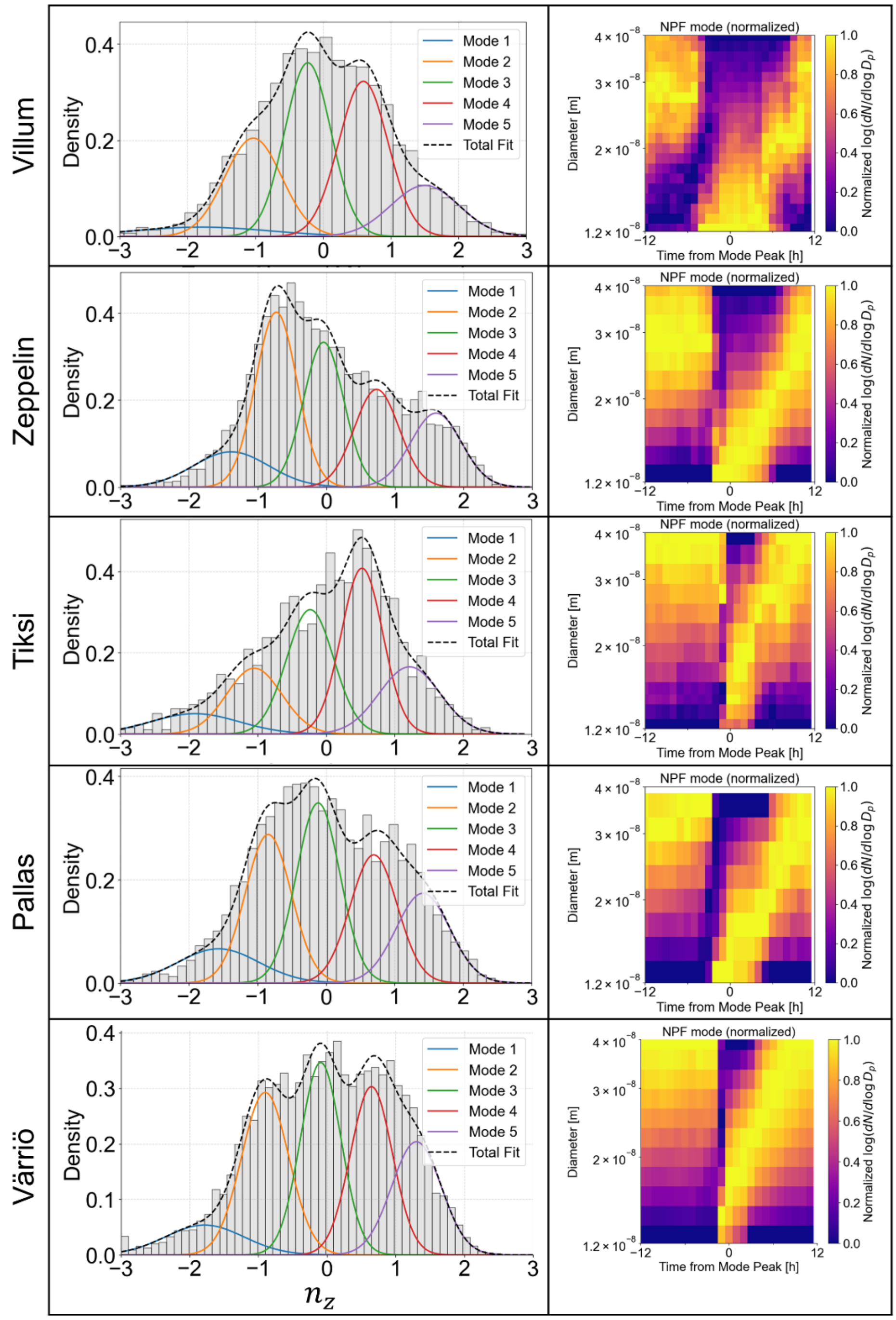

Figure S6. Left column: Density distribution of n_z and its five fitted Gaussian curves at (from top to bottom) Villum, Zeppelin, Tiksi, Pallas, and Värriö, sharing the same x-axis. Right column: Median surface daily plots of the days under the NPF mode (Mode 5) normalized across each time segment (vertically) and aligning the peak values of the 12.5 nm size bin to 0 hour.

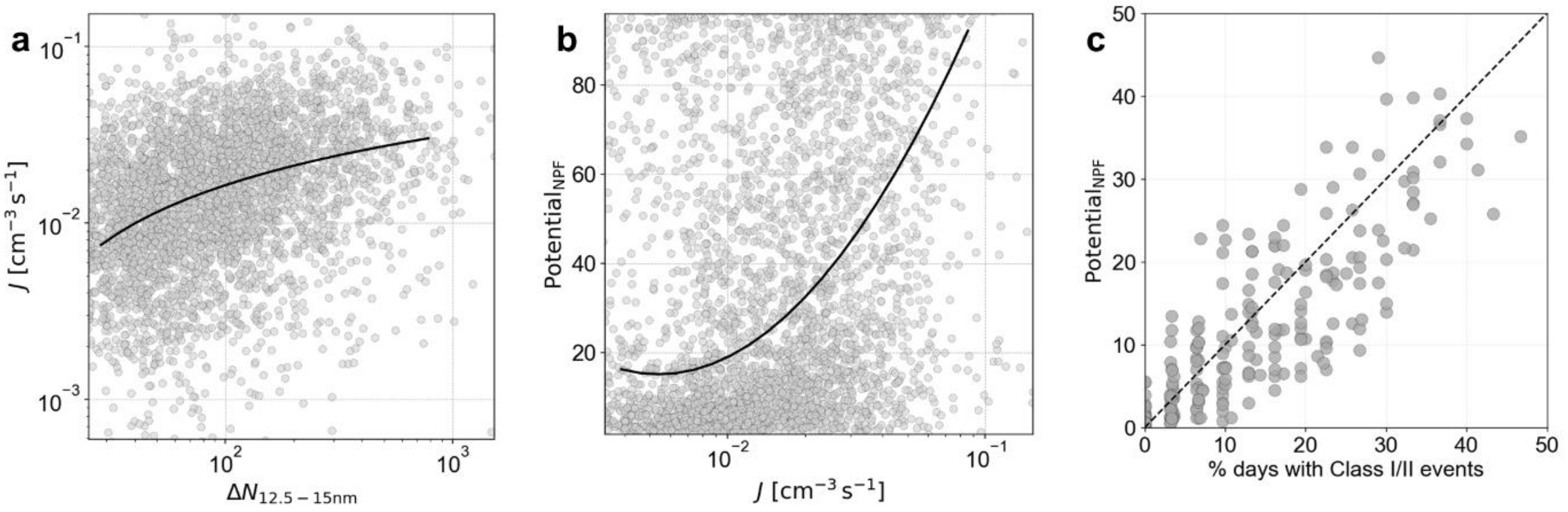

Figure S7. (a) Formation rates of 15 nm particles (J_{15}) against $\Delta N_{12.5-15\,nm}$ values, and (b) $Potential_{NPF}$ against formation rates of 15 nm particles (J_{15}) across all stations. The black lines indicate the median while the grey scatter indicates raw datapoints. (c) Monthly values of $Potential_{NPF}$ at Värriö against the corresponding monthly value of the percentage of days observed with Class I/II events (according to Dal Maso et al. (2005)) observed. Dashed line is the 1:1 ratio line.

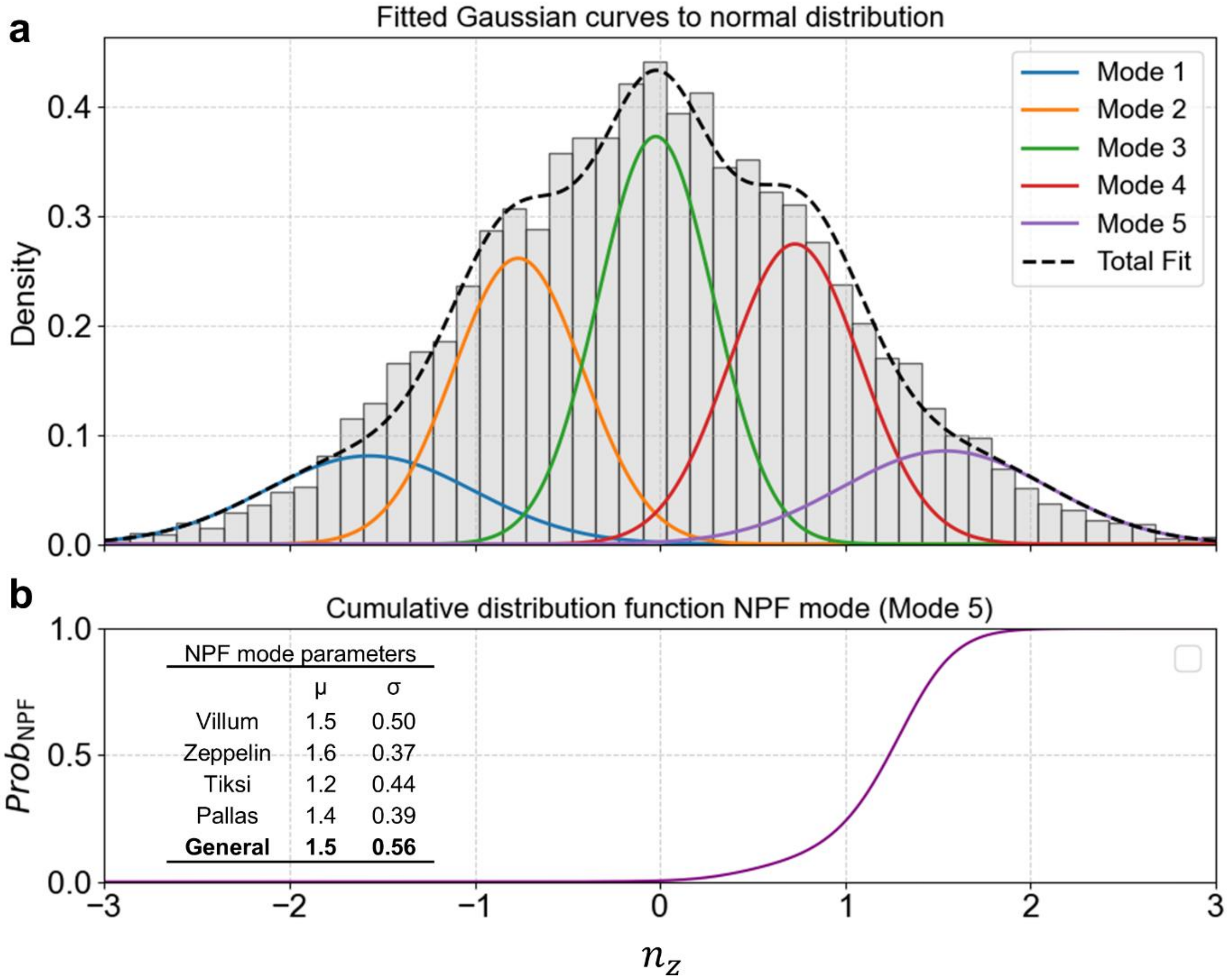

Figure S8. a) A general normal distribution of values of n_z and its five fitted KDE curves. b) Cumulative distribution function of the NPF mode (Mode 5 in (a)) to relate $Potential_{NPF}$ as a function of n_z. Inset table show the parameters (μ and σ) of the KDE distribution fitted to the NPF modes at Villum, Zeppelin, Tiksi, Pallas and the general case in (a).

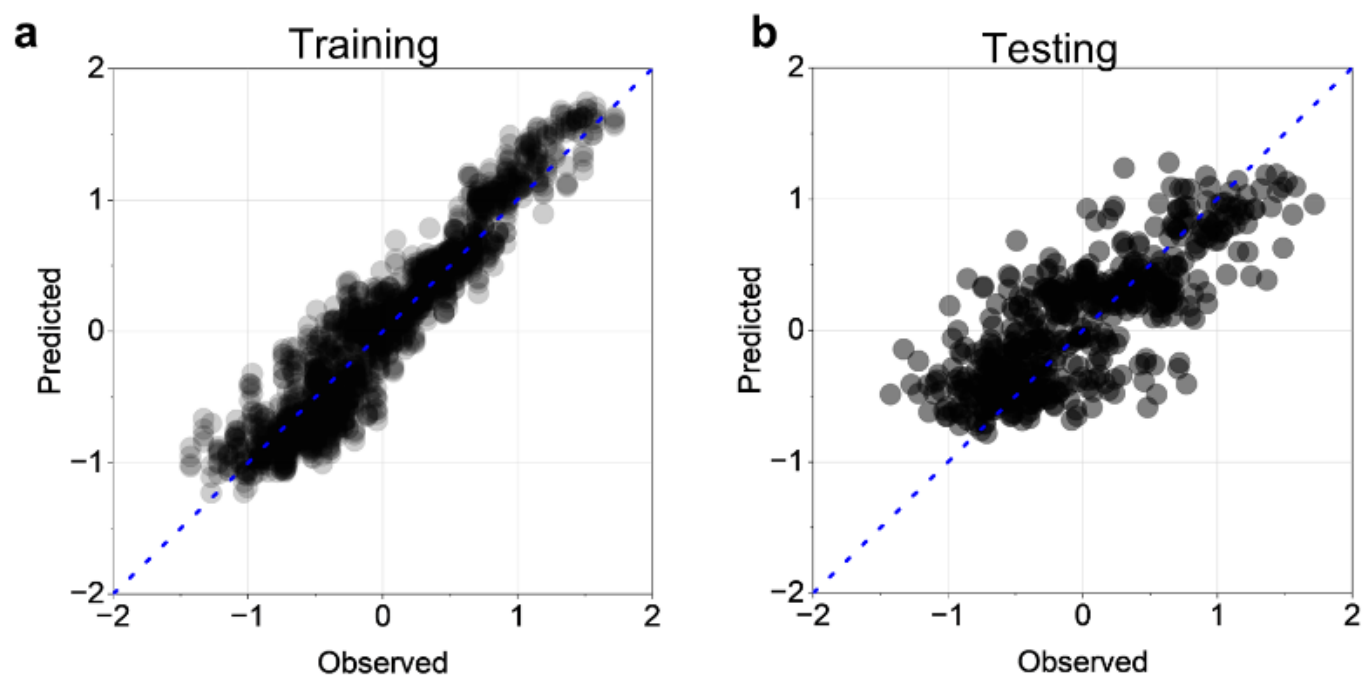

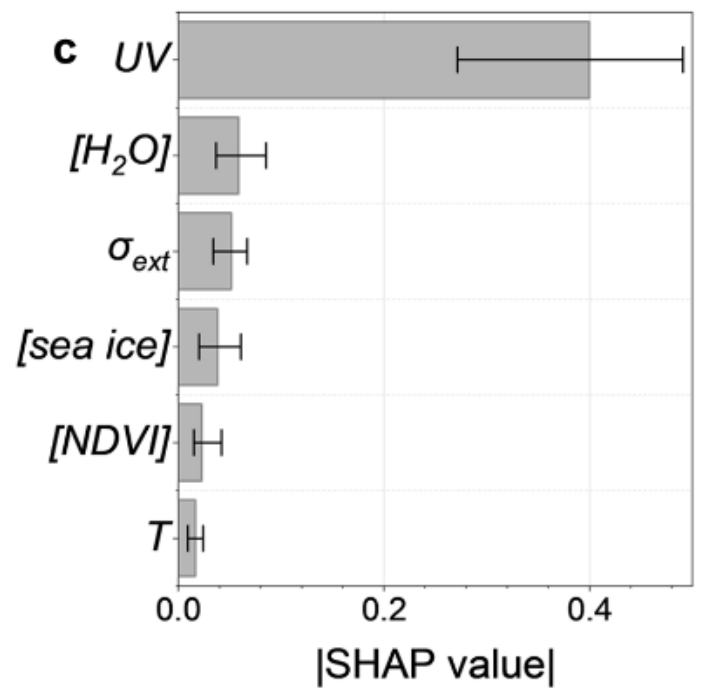

	Training	Testing
n	1836	459
R^2	0.84	0.65
MAE	0.18	0.31
Bias	-0.06	-0.05

Figure S9. Observed versus predicted n_z values for the (a) training datasets and (b) testing datasets, with the testing results obtained by combining the hold-out predictions from the five folds of the cross-validation. (c) Median absolute SHAP values of the variables in Eq.4 that were trained in the XGBoost algorithm, with error bars indicating the interquartile range. Higher SHAP values indicate larger importance of the variable in predicting the target variable. Inset table shows the accuracy metrics of the training and testing datasets: n, R^2, MAE (mean absolute error), and bias.

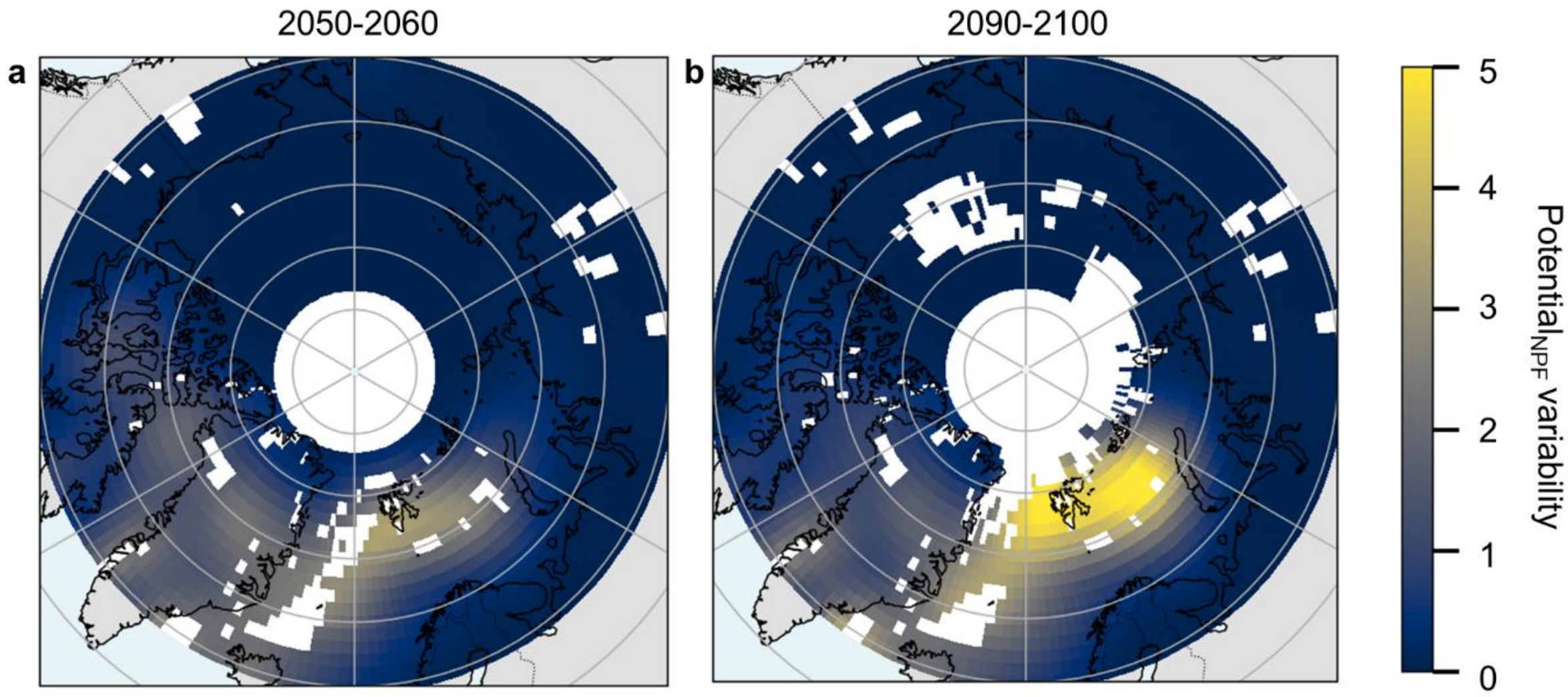

Figure S10. The variability in (a) 2050-2060 and (b) 2090-2100 $Potential_{NPF}$ temperature-sensitivity estimates based on changes for SSP 2-4.5. The variability here indicates the half-IQR spread around each pixel, or the larger absolute difference of $Potential_{NPF}$ between the median and the upper/lower quartile estimates.

Table S1. Median aerosol parameters across eight Arctic locations separated by seasons (DJF: December-February, MAM: March-May, JJA: June-August, SON: September-November). Parameters are $N_{12.5\text{-}25\ nm}$: nucleation mode particles concentration; $N_{25\text{-}100\ nm}$: Aitken mode particles concentration; $N_{100\text{-}500\ nm}$: accumulation mode particles concentration; CS: condensation sink; GR: growth rate at 15-20 nm; J: formation rate at 15 nm; and $\Delta N_{12.5\text{-}15\ nm}$: diurnal variation in 12.5-15 nm particle concentration. Brackets indicate the interquartile range of the variable. Values represent medians calculated over the full available observational period for each site or campaign, with brackets indicating the interquartile range of each variable. The duration of the datasets differed: Zeppelin (08/2010–10/2024), Villum (07/2010–09/2020), Tiksi (07/2010–10/2018), Pallas (01/2012–06/2021), Värriö (01/2003–06/2024), Alert (03/2011–12/2018), Utqiaġvik (10/2019–12/2024), Central Arctic (08/2018–06/2023).

		$N_{12.5\text{-}25nm}$ [cm^{-3}]	$N_{25\text{-}100nm}$ [cm^{-3}]	$N_{100\text{-}500nm}$ [cm^{-3}]	CS [$\times10^{-4}$ s^{-1}]	GR [nm hr^{-1}]	J [$\times10^{-3}$ cm^{-3} s^{-1}]	$\Delta N_{12.5\text{-}15nm}$ [cm^{-3}]
DJF	Alert	2.5 (1.8-4.1)	28 (20-38)	62 (42-88)	7.9 (5.5-12)			
	Central Arctic	5.3 (4.7-8.6)	34 (22-59)	96 (65-132)	13 (9.7-19)			
	Pallas	19 (11-36)	77 (38-134)	34 (17-80)	5.2 (2.8-11)			
	Tiksi	16 (6.2-61)	121 (67-361)	156 (112-254)	20 (16-28)			
	Utqiagvik	9.0 (3.8-38)	27 (15-76)	48 (28-60)	5.9 (3.7-8.9)			
	Värriö	13 (7.3-24)	79 (41-148)	95 (45-189)	13 (6.6-25)			
	Villum	5.8 (3.4-9.6)	35 (26-38)	72 (51-107)	9.5 (6.2-14)			
	Zeppelin	5.4 (3.2-9.5)	19 (11-33)	36 (19-77)	5.1 (2.4-11)			
MAM	Alert	4.6 (2.6-9.4)	44 (33-59)	99 (64-129)	12 (8.6-16)	0.9 (0.7-2.4)	0.8 (0.4-1.9)	0.7 (0.1-2.6)
	Central Arctic	5.1 (4.6-8.7)	42 (28-55)	101 (82-115)	12 (10-14)			0.5 (0.2-5.4)
	Pallas	73 (31-193)	207 (78-508)	91 (49-178)	13 (7.2-25)	2.4 (2.1-2.6)	21 (11-31)	51 (11-174)
	Tiksi	24 (6.7-94)	147 (77-475)	202 (126-420)	24 (17-34)	3.1 (2.5-4.9)	26 (11-31)	8.1 (1.3-34)
	Utqiagvik	108 (11-445)	79 (28-214)	97 (51-127)	13 (7.6-19)			61 (1.3-237)
	Värriö	61 (23-179)	283 (108-596)	143 (75-257)	20 (11-35)	2.4 (2.1-3.2)	19 (11-35)	39 (9.6-112)
	Villum	8.4 (4.5-16)	51 (34-73)	93 (63-139)	11 (7.6-17)	1.2 (0.8-1.5)	3.2 (1.6-7.0)	1.7 (0.5-5.7)
	Zeppelin	10 (4.9-28)	51 (30-87)	88 (53-132)	11 (6.5-16)	1.0 (0.7-1.2)	6.5 (3.1-14)	2.3 (0.5-14)
JJA	Alert	19 (9.6-39)	105 (49-177)	32 (18-59)	4.2 (2.5-6.4)	1.1 (0.7-1.9)	1.8 (1.0-3.5)	2.6 (0.8-8.5)
	Central Arctic	26 (9.8-39)	71 (27-138)	29 (11-48)	4.5 (1.9-6.7)	2.4 (1.5-2.5)	1.3 (0.8-1.6)	3.9 (0.6-8.6)
	Pallas	38 (16-108)	474 (258-795)	217 (101-491)	32 (15-61)	2.4 (2.1-2.6)	20 (11-31)	19 (5.5-75)
	Tiksi	27 (11-74)	262 (129-504)	100 (56-220)	14 (7.8-30)	1.9 (1.7-2.6)	12 (9.5-19)	17 (4.9-44)
	Utqiagvik	60 (12-214)	114 (34-214)	38 (18-73)	5.2 (2.7-10)	1.6 (1.4-2.5)	22 (12-28)	58 (6.2-158)
	Värriö	28 (12-75)	460 (255-734)	255 (117-515)	36 (19-59)	2.5 (2.1-3.3)	19 (10-31)	12 (3.1-42)
	Villum	35 (15-88)	102 (60-172)	35 (17-55)	4.4 (2.6-7.0)	1.0 (0.6-1.8)	4.7 (2.4-8.7)	12 (2.9-34)
	Zeppelin	34 (14-95)	116 (57-226)	49 (25-86)	6.2 (3.4-11)	1.1 (0.9-1.3)	8.5 (4.0-16)	22 (4.5-79)
SON	Alert	4.1 (2.3-12)	23 (15-37)	29 (18-51)	5.1 (2.5-9.5)	1.5 (0.8-3.5)	0.9 (0.6-1.7)	0.4 (0.1-1.7)
	Central Arctic	12 (5.9-29)	21 (11-33)	20 (11-47)	2.8 (1.6-6.1)	3.2 (2.7-3.3)	1.6 (1.2-2.1)	2.3 (0.4-7.8)
	Pallas	29 (12-96)	131 (51-366)	34 (13-86)	5.5 (2.6-14)	2.4 (2.1-2.6)	14 (7.5-24)	13 (3.4-51)
	Tiksi	17 (9.0-37)	123 (68-215)	99 (64-145)	14 (8.9-20)	1.9 (1.6-2.7)	8.5 (4.2-13)	5.1 (1.4-18)
	Utqiagvik	11 (4.4-46)	26 (12-69)	34 (17-53)	3.5 (2.0-7.2)	2.1 (1.5-2.1)	13 (5.3-29)	3.3 (0.8-21)
	Värriö	22 (9.2-61)	130 (53-343)	68 (36-146)	10 (5.4-20)	2.4 (2.1-3.0)	13 (6.7-22)	8.2 (1.7-34)
	Villum	13 (6.3-31)	40 (28-67)	41 (27-62)	4.7 (3.0-7.7)	1.3 (0.8-2.5)	4.4 (1.1-9.9)	2.6 (0.6-7.4)
	Zeppelin	5.5 (2.8-13)	20 (11-40)	23 (11-44)	2.8 (1.4-5.5)	1.2 (0.9-1.3)	3.3 (2.4-7.5)	0.7 (0.2-2.6)

Table S2. Percentage change per year in aerosol parameters from 2010 to 2019 separated by seasons (DJF: December-February, MAM: March-May, JJA: June-August, SON: September-November) with its interquartile range indicated in brackets. Trends were calculated from fitted Theil–Sen trend lines and expressed in percentage change/year. Asterisks indicate that trend is statistically significant (Mann-Kendall seasonal test, $p < 0.1$). Parameters are $N_{12.5\text{-}25\ nm}$: nucleation mode particles concentration; $N_{25\text{-}100\ nm}$: Aitken mode particles concentration; $N_{100\text{-}500\ nm}$: accumulation mode particles concentration; CS: condensation sink; GR: growth rate at 15-20 nm; J: formation rate at 15 nm; and $\Delta N_{12.5\text{-}15\ nm}$: diurnal variation in 12.5-15 nm particle concentration. The calculated changes in $N_{100\text{-}500\ nm}$ and CS of Villum were only from 2010-2018 due to data gaps.

		$N_{12.5\text{-}25nm}$	$N_{25\text{-}100nm}$	$N_{100\text{-}500nm}$	CS	GR	J	$\Delta N_{12.5\text{-}15nm}$
DJF	Alert	6.3 (4.5 – 7.9)*	-1.3 (-2.4 – -0.2)	-3.5 (-4.8 – -2.2)*	-3.4 (-5.2 – -1.4)*			
	Pallas	1.7 (0.1 – 3.1)	-6.6 (-8.1 – -5.1)*	-8.8 (-11 – -6.9)*	-8.7 (-10 – -7.0)*			
	Tiksi	-20 (-25 – -14)*	1.5 (-2.7 – 5.8)	-1.3 (-4.0 – 1.4)	2.2 (-0.2 – 4.5)			
	Värriö	-0.9 (-1.9 – 0.2)	-5.7 (-6.7 – -4.6)*	-9.7 (-11 – -8.7)*	-9.7 (-11 – -8.7)*			
	Villum	3.0 (1.7 – 4.1)*	1.4 (0.7 – 2.2)*	1.1 (0.0 – 2.2)	0.6 (-0.4 – 1.5)			
	Zeppelin	0.0 (-1.4 – 1.4)	-2.0 (-3.2 – -0.7)	-3.9 (-5.4 – -2.4)*	-4.0 (-5.6 – -2.4)*			
MAM	Alert	3.9 (1.0 – 6.6)	-4.2 (-5.2 – -3.1)*	-3.0 (-4.1 – -2.0)*	-2.8 (-4.1 – -1.4)*	-6.9 (-14 – -0.7)	-0.9 (-10 – 11)	26 (20 – 32)*
	Pallas	-1.3 (-3.5 – 0.9)	-2.5 (-4.4 – -0.6)	-0.3 (-2.0 – 1.4)	-0.6 (-2.2 – 1.0)	0.0 (-0.4 – 0.3)	-2.9 (-4.7 – -1.1)	-0.7 (-3.8 – 2.4)
	Tiksi	-10 (-16 – -2.5)	-8.6 (-13 – -3.9)	-2.9 (-6.0 – 0.2)	-7.6 (-9.9 – -5.4)*	3.1 (-7.5 – 14)	19 (-1.4 – 57)	-3.9 (-13 – 5.8)
	Värriö	1.9 (-0.0 – 3.3)	0.9 (-0.5 – 2.2)	-0.2 (-1.3 – 0.9)	-0.5 (-1.6 – 0.5)	-0.1 (-0.6 – 0.4)	0.1 (-1.4 – 1.5)	1.5 (-0.6 – 3.6)
	Villum	9.2 (7.6 – 11)*	1.9 (0.9 – 2.8)*	0.3 (-0.7 – 1.4)	-2.6 (-3.5 – -1.8)*	4.9 (0.8 – 13)	3.7 (-7.6 – 14)	19 (16 – 22)*
	Zeppelin	7.1 (5.1 – 9.0)*	3.8 (2.4 – 5.1)*	-2.7 (-3.7 – -1.7)*	-3.4 (-4.5 – -2.4)*	2.0 (0.6 – 5.2)	4.7 (-2.5 – 13)	12 (7.5 – 17)*
JJA	Alert	4.1 (1.7 – 6.5)	-0.8 (-2.8 – 1.1)	-2.1 (-4.3 – 0.2)	-4.4 (-5.9 – -2.9)*	3.0 (-2.4 – 8.1)	-0.5 (-7.3 – 7.2)	16 (12 – 20)*
	Pallas	4.1 (1.5 – 6.7)*	0.4 (-1.3 – 2.1)	-4.8 (-6.9 – -2.8)*	-4.5 (-6.2 – -2.8)*	-0.2 (-0.6 – 0.4)	5.3 (2.4 – 8.2)*	12 (8.1 – 16)*
	Tiksi	-4.7 (-7.3 – -2.3)*	-3.9 (-5.5 – -2.2)*	-1.9 (-3.6 – -0.1)	-2.4 (-4.2 – -0.6)	0.4 (-0.4 – 1.8)	1.4 (-1.3 – 4.6)	-1.3 (-4.5 – 2.0)
	Värriö	-2.7 (-4.3 – -1.2)*	1.0 (0.0 – 2.0)	1.0 (-0.1 – 2.2)	1.1 (0.1 – 2.1)	0.7 (0.0 – 1.4)	0.5 (-1.3 – 2.1)	-0.2 (-2.4 – 2.1)
	Villum	9.9 (8.0 – 12)*	5.2 (4.0 – 6.4)*	5.1 (3.6 – 6.6)*	4.5 (3.5 – 5.5)*	0.2 (-1.8 – 2.7)	3.5 (0.9 – 6.4)	14 (11 – 17)*
	Zeppelin	-1.4 (-3.5 – 0.8)	0.5 (-1.0 – 2.0)	2.6 (1.3 – 4.0)*	2.7 (1.3 – 4.2)*	-0.6 (-1.6 – 0.1)	-0.3 (-3.3 – 2.5)	-2.3 (-5.4 – 1.1)
SON	Alert	-0.7 (-3.0 – 1.8)	-0.3 (-1.6 – 1.0)	-0.1 (-1.4 – 1.4)	3.3 (1.4 – 5.1)*	6.7 (-2.7 – 19)	-6.2 (-13 – 1.0)	13 (8 – 18)*
	Pallas	-5.0 (-7.3 – -2.8)*	-8.7 (-11 – -6.8)*	-8.7 (-11 – -6.6)*	-8.2 (-10 – -6.3)*	-0.1 (-0.5 – 0.2)	0.2 (-2.7 – 3.5)	0.3 (-3.0 – 3.6)
	Tiksi	-2.7 (-5.1 – -0.4)	-3.8 (-5.5 – -2.2)*	-2.6 (-4.0 – -1.3)*	-2.0 (-3.4 – -0.7)	5.8 (3.1 – 14)	-5.3 (-10 – 1.3)	2.0 (-1.9 – 6.3)
	Värriö	-3.1 (-4.6 – -1.7)*	-4.6 (-6.0 – -3.2)*	-3.8 (-4.9 – -2.6)*	-3.2 (-4.3 – -2.1)*	0.1 (-0.4 – 0.7)	3.2 (1.0 – 5.2)	-4.4 (-6.6 – -2.1)*
	Villum	4.4 (2.2 – 6.6)*	1.7 (0.5 – 2.8)	-0.2 (-1.7 – 1.3)	-3.8 (-5.1 – -2.6)*	17 (-0.6 – 30)	33 (16 – 50)	0.8 (-2.8 – 4.4)
	Zeppelin	-1.7 (-3.5 – 0.1)	-5.0 (-6.4 – -3.6)*	-5.7 (-7.0 – -4.4)*	-5.8 (-7.2 – -4.4)*	1.1 (-1.6 – 6.8)	-11 (-27 – 5.5)	-5.7 (-8.4 – -3.0)*

Table S3: Area-weighted median values of estimated change in $Potential_{NPF}$ in 2050-2060 and 2090-2100 under the SSP2-4.5 climate scenario compared to its baseline level (2006-2021). The variability range indicate the interquartile range of possible outcomes while the confidence range indicate 95% confidence interval around the median value. Refer to Text S4 for more details on the uncertainty ranges.

	2050-2060			2090-2100		
		Variability range	Confidence range		Variability range	Confidence range
High Arctic	0.6	-3.3 – 9.5	0.1 – 1.3	0.9	-2.6 – 10.8	0.7 – 1.9
Maritime Arctic	-3.4	-11.8 – 8.7	-5.9 – -2.9	-6.6	-13.0 – 0.2	-8.3 – -6.5
Continental Arctic	0.0	-0.5 – 1.4	-0.1 – 0.1	0.0	-0.6 – 1.4	-0.1 – 0.1

Table S4. Information on the list of long-term observational sites and research expeditions where particle number size distribution data were obtained from. More information about the sites, instrumentation and data processing can be found in the accompanying reference(s).

Site / Campaign	Location	Coordinates	Data period (data days)	Instrumentation	Reference(s)
Zeppelin	Svalbard	78.9°N, 11.9°E	08/2010 – 10/2024 (4719 d)	Custom twin DMPS, TSI 3010, TSI 3772	51
Villum	Northeast Greenland	81.6°N, 16.7°W	07/2010 – 09/2020 (2499 d)	Custom DMPS, TSI 3772, TSI 3010	52
Tiksi	North Siberia	71.6°N, 128.9°E	07/2010 – 10/2018 (1484 d)	Custom twin DMPS, TSI 3772, TSI 3776	53
Pallas	Finnish Lapland	70.0°N, 24.1°E	01/2012 – 06/2021 (3154 d)	Custom DMPS, TSI 3010	54
Värriö		67.8°N, 29.6°E	01/2003 – 06/2024 (7744 d)	Custom DMPS, TSI 3025	55,56
Alert	Nunavut	82.5°N, 62.3°W	03/2011 – 12/2018 (1548 d)	SMPS, TSI 3034	57
Utqiaġvik	North Alaska	71.3°N, 156.6°W	10/2019 – 12/2024 (347 d)	MPSS, TSI 3010	58,59
Arctic Ocean 2018			08/2018 – 09/2018 (36 d)	Custom DMPS, TSI 3776	60
MOSAiC	Central Arctic Ocean	80°N to 90°N	10/2019 – 09/2020 (157 d)	SMPS, TSI 3936	30,61
ARTofMELT			05/2023 – 06/2023 (17 d)	Custom DMPS, TSI 3776	62

Table S5. Comparison of CCN number concentration ($N_{CCN\ all}$) values in cm^{-3} at supersaturation(SS) = 0.2 obtained by CALIPSO over Zeppelin versus that measured at a concurrent ground observation site from 2007 to 2015 [44]. The CALIPSO-derived $N_{CCN\ all}$ were sum of all aerosol types and had been aggregated from the ground level to the boundary layer height. Values presented are seasonal median values with brackets indicating the interquartile range.

	CALIPSO-derived $N_{CCN\ all}$	Ground observation
March – May	101 (53 – 115)	107 (102 – 126)
June – August	55 (20 – 68)	56 (52 – 58)
March – August	54 (44 – 69)	80 (71 – 89)

Supplementary References

1. Bhandari, R. *et al.* Identification of New Particle Formation Events Using a You Only Look Once (YOLO) Deep Learning Algorithm. *ACS EST Air* **2**, 1202–1213 (2025).
2. Dal Maso, M. *et al.* Formation and growth of fresh atmospheric aerosols: eight years of aerosol size distribution data from SMEAR II, Hyytiälä, Finland. *Boreal environment research* **10**, 323 (2005).
3. Tammet, H., Komsaare, K. & Hõrrak, U. Intermediate ions in the atmosphere. *Atmospheric Research* **135–136**, 263–273 (2014).
4. Tuovinen, S., Lampilahti, J., Kerminen, V.-M. & Kulmala, M. Intermediate ions as indicator for local new particle formation. *Aerosol Research* **2**, 93–105 (2024).
5. Dall´Osto, M. *et al.* Arctic sea ice melt leads to atmospheric new particle formation. *Sci Rep* **7**, 3318 (2017).
6. Heintzenberg, J., Tunved, P., Galí, M. & Leck, C. New particle formation in the Svalbard region 2006–2015. *Atmos. Chem. Phys.* **17**, 6153–6175 (2017).
7. Kulmala, M. *et al.* The first estimates of global nucleation mode aerosol concentrations based on satellite measurements. *Atmos. Chem. Phys.* **11**, 10791–10801 (2011).
8. Sundström, A.-M. *et al.* Characterization of satellite-based proxies for estimating nucleation mode particles over South Africa. *Atmos. Chem. Phys.* **15**, 4983–4996 (2015).
9. Dada, L. *et al.* Sources and sinks driving sulfuric acid concentrations in contrasting environments: implications on proxy calculations. *Atmos. Chem. Phys.* **20**, 11747–11766 (2020).
10. Li, X. *et al.* Parameterization of particle formation rates in distinct atmospheric environments. *Aerosol Research* **3**, 271–291 (2025).
11. Petäjä, T. *et al.* Sulfuric acid and OH concentrations in a boreal forest site. *Atmos. Chem. Phys.* **9**, 7435–7448 (2009).
12. Beck, L. J. *et al.* Differing Mechanisms of New Particle Formation at Two Arctic Sites. *Geophysical Research Letters* **48**, e2020GL091334 (2021).
13. Jokinen, T. *et al.* Measurement report: Long-term measurements of aerosol precursor concentrations in the Finnish subarctic boreal forest. *Atmos. Chem. Phys.* **22**, 2237–2254 (2022).
14. Boyer, M. *et al.* The annual cycle and sources of relevant aerosol precursor vapors in the central Arctic during the MOSAiC expedition. *Atmos. Chem. Phys.* **24**, 12595–12621 (2024).
15. Dall´Osto, M. *et al.* Regions of open water and melting sea ice drive new particle formation in North East Greenland. *Sci Rep* **8**, (2018).
16. He, X.-C. *et al.* Role of iodine oxoacids in atmospheric aerosol nucleation. *Science* **371**, 589–595 (2021).
17. Kecorius, S. *et al.* New particle formation and its effect on cloud condensation nuclei abundance in the summer Arctic: a case study in the Fram Strait and Barents Sea. *Atmos. Chem. Phys.* **19**, 14339–14364 (2019).
18. Kerminen, V.-M. *et al.* Atmospheric new particle formation and growth: review of field observations. *Environ. Res. Lett.* **13**, 103003 (2018).
19. Pernov, J. B. *et al.* Pan-Arctic methanesulfonic acid aerosol: source regions, atmospheric drivers, and future projections. *npj Clim Atmos Sci* **7**, 166 (2024).
20. Smith, G. L. *et al.* Clouds and Earth Radiant Energy System (CERES), a review: Past, present and future. *Advances in Space Research* **48**, 254–263 (2011).
21. Su, W., Charlock, T. P. & Rose, F. G. Deriving surface ultraviolet radiation from CERES surface and atmospheric radiation budget: Methodology. *J. Geophys. Res.* **110**, (2005).
22. Doelling, D. R. *et al.* Advances in Geostationary-Derived Longwave Fluxes for the CERES Synoptic (SYN1deg) Product. *Journal of Atmospheric and Oceanic Technology* **33**, 503–521 (2016).
23. Rienecker, M. M. *et al.* MERRA: NASA's Modern-Era Retrospective Analysis for Research and Applications. *Journal of Climate* **24**, 3624–3648 (2011).
24. Young, S. A. *et al.* Extinction and optical depth retrievals for CALIPSO's Version 4 data release. *Atmos. Meas. Tech.* **11**, 5701–5727 (2018).

25. Tesche, M. *et al.* Reconciling aerosol light extinction measurements from spaceborne lidar observations and in situ measurements in the Arctic. *Atmos. Chem. Phys.* **14**, 7869–7882 (2014).
26. Danielson, J. J. & Gesch, D. B. *Global Multi-Resolution Terrain Elevation Data 2010 (GMTED2010)*. https://pubs.usgs.gov/publication/ofr20111073 (2011) doi:10.3133/ofr20111073.
27. Didan, K. MODIS/Terra Vegetation Indices Monthly L3 Global 1km SIN Grid V061. NASA Land Processes Distributed Active Archive Center https://doi.org/10.5067/MODIS/MOD13A3.061 (2021).
28. Kunkee, D. B. *et al.* Design and Evaluation of the First Special Sensor Microwave Imager/Sounder. *IEEE Trans. Geosci. Remote Sensing* **46**, 863–883 (2008).
29. Marcilio, W. E. & Eler, D. M. From explanations to feature selection: assessing SHAP values as feature selection mechanism. in *2020 33rd SIBGRAPI Conference on Graphics, Patterns and Images (SIBGRAPI)* 340–347 (IEEE, Recife/Porto de Galinhas, Brazil, 2020). doi:10.1109/SIBGRAPI51738.2020.00053.
30. Boyer, M. *et al.* A full year of aerosol size distribution data from the central Arctic under an extreme positive Arctic Oscillation: insights from the Multidisciplinary drifting Observatory for the Study of Arctic Climate (MOSAiC) expedition. *Atmos. Chem. Phys.* **23**, 389–415 (2023).
31. Fanourgakis, G. S. *et al.* Evaluation of global simulations of aerosol particle and cloud condensation nuclei number, with implications for cloud droplet formation. *Atmos. Chem. Phys.* **19**, 8591–8617 (2019).
32. Virtanen, A. *et al.* High sensitivity of cloud formation to aerosol changes. *Nat. Geosci.* **18**, 289–295 (2025).
33. Choudhury, G. & Tesche, M. A first global height-resolved cloud condensation nuclei data set derived from spaceborne lidar measurements. *Earth Syst. Sci. Data* **15**, 3747–3760 (2023).
34. Choudhury, G. & Tesche, M. Assessment of CALIOP-Derived CCN Concentrations by In Situ Surface Measurements. *Remote Sensing* **14**, 3342 (2022).
35. Koike, M. *et al.* Year-Round In Situ Measurements of Arctic Low-Level Clouds: Microphysical Properties and Their Relationships With Aerosols. *JGR Atmospheres* **124**, 1798–1822 (2019).
36. Herenz, P. *et al.* Measurements of aerosol and CCN properties in the Mackenzie River delta (Canadian Arctic) during spring–summer transition in May 2014. *Atmos. Chem. Phys.* **18**, 4477–4496 (2018).
37. Jung, C. H. *et al.* The seasonal characteristics of cloud condensation nuclei (CCN) in the arctic lower troposphere. *Tellus B: Chemical and Physical Meteorology* **70**, 1513291 (2018).
38. Massling, A. *et al.* Measurement report: High Arctic aerosol hygroscopicity at sub- and supersaturated conditions during spring and summer. *Atmos. Chem. Phys.* **23**, 4931–4953 (2023).
39. Moore, R. H. *et al.* Hygroscopicity and composition of Alaskan Arctic CCN during April 2008. *Atmos. Chem. Phys.* **11**, 11807–11825 (2011).
40. Motos, G. *et al.* Aerosol and dynamical contributions to cloud droplet formation in Arctic low-level clouds. *Atmos. Chem. Phys.* **23**, 13941–13956 (2023).
41. Park, J. *et al.* Shipborne observations reveal contrasting Arctic marine, Arctic terrestrial and Pacific marine aerosol properties. *Atmos. Chem. Phys.* **20**, 5573–5590 (2020).
42. Yao, W. *et al.* Seasonal cycles and long-term trends of arctic tropospheric aerosols based on CALIPSO lidar observations. *Environmental Research* **216**, 114613 (2023).
43. Xi, X. *et al.* Evaluation of the Planetary Boundary Layer Height From ERA5 Reanalysis With MOSAiC Observations Over the Arctic Ocean. *JGR Atmospheres* **129**, e2024JD040779 (2024).
44. Yoon, Y. Measurement of Cloud_condensation_nuclei_number_concentration at Zeppelin mountain (Ny-Ålesund). NILU, Department for Atmospheric Research, EBAS https://doi.org/10.48597/A4MR-N5SU (2019).
45. Kim, J. *et al.* Seasonal variations in physical characteristics of aerosol particles at the King Sejong Station, Antarctic Peninsula. *Atmos. Chem. Phys.* **17**, 12985–12999 (2017).
46. Mamouri, R.-E. & Ansmann, A. Potential of polarization lidar to provide profiles of CCN- and INP-relevant aerosol parameters. *Atmos. Chem. Phys.* **16**, 5905–5931 (2016).
47. Paramonov, M. *et al.* A synthesis of cloud condensation nuclei counter (CCNC) measurements within the EUCAARI network. *Atmos. Chem. Phys.* **15**, 12211–12229 (2015).
48. Box, J. E. *et al.* Key indicators of Arctic climate change: 1971–2017. *Environ. Res. Lett.* **14**, 045010 (2019).

49. Cai, Z. *et al.* Arctic Warming Revealed by Multiple CMIP6 Models: Evaluation of Historical Simulations and Quantification of Future Projection Uncertainties. *Journal of Climate* **34**, 4871–4892 (2021).
50. Copernicus Climate Change Service. ERA5 monthly averaged data on single levels from 1940 to present. Copernicus Climate Change Service (C3S) Climate Data Store (CDS) https://doi.org/10.24381/CDS.F17050D7 (2019).
51. Tunved, P., Ström, J. & Krejci, R. Arctic aerosol life cycle: linking aerosol size distributions observed between 2000 and 2010 with air mass transport and precipitation at Zeppelin station, Ny-Ålesund, Svalbard. *Atmos. Chem. Phys.* **13**, 3643–3660 (2013).
52. Nguyen, Q. T. *et al.* Seasonal variation of atmospheric particle number concentrations, newparticle formation and atmospheric oxidation capacity at the high Arcticsite Villum Research Station, Station Nord. *Atmos. Chem. Phys.* **16**, 11319–11336 (2016).
53. Asmi, E. *et al.* Aerosol size distribution seasonal characteristics measured in Tiksi, Russian Arctic. *Atmos. Chem. Phys.* **16**, 1271–1287 (2016).
54. Asmi, E. *et al.* Secondary new particle formation in Northern Finland Pallas site between the years 2000 and 2010. *Atmos. Chem. Phys.* **11**, 12959–12972 (2011).
55. Ruuskanen, T. M. *et al.* Atmospheric trace gas and aerosol particle concentration measurements in Eastern Lapland, Finland 1992–2001. *Boreal environment research* **8**, 335 (2003).
56. Kyrö, E.-M. *et al.* Trends in new particle formation in eastern Lapland, Finland: effect of decreasing sulfur emissions from Kola Peninsula. *Atmos. Chem. Phys.* **14**, 4383–4396 (2014).
57. Croft, B. *et al.* Processes controlling the annual cycle of Arctic aerosol number and size distributions. *Atmos. Chem. Phys.* **16**, 3665–3682 (2016).
58. Freud, E. *et al.* Pan-Arctic aerosol number size distributions: seasonality and transport patterns. *Atmos. Chem. Phys.* **17**, 8101–8128 (2017).
59. Russell, L. M. *et al.* Aerosol Measurements at Utqiagvik, Alaska, from 2022 to 2027 for Sustaining The Utqiagvik Aerosol Record of Decades (STUARD). UC San Diego Library Digital Collections https://doi.org/10.6075/J0W66KZF (2024).
60. Karlsson, L. *et al.* Physical and Chemical Properties of Cloud Droplet Residuals and Aerosol Particles During the Arctic Ocean 2018 Expedition. *JGR Atmospheres* **127**, e2021JD036383 (2022).
61. Kuang, C., Singh, A., Howie, J., Salwen, C. & Hayes, C. Scanning Mobility Particle Sizer, b1 level. Atmospheric Radiation Measurement (ARM) Archive, Oak Ridge National Laboratory (ORNL), Oak Ridge, TN (US); ARM Data Center, Oak Ridge National Laboratory (ORNL), Oak Ridge, TN (United States) https://doi.org/10.5439/1476898 (2021).
62. Freitas, G. P. *et al.* A comprehensive characterisation of natural aerosol sources in the high Arctic during the onset of sea ice melt. *Faraday Discuss.* **258**, 120–146 (2025).
