# Supplementary material for: Contrasting aerosol–cloud feedbacks across a warming Arctic
Source: Nat Geosci. 2026 Sep 28;19(11):1292–300. doi: 10.1038/s41561-026-02114-x (PMC13739744; doi:10.1038/s41561-026-02114-x)
Supplement: Supplementary file 2 [file 41561_2026_2114_MOESM2_ESM.pdf]

nature portfolio

Peer Review File

Contrasting aerosol-cloud feedbacks across a warming Arctic

Corresponding Author: Mr Samuel Chua

Version 0:

Reviewer comments:

Reviewer #1

(Remarks to the Author)
The authors present an intriguing method for estimating future Arctic CCN concentrations based on a combination of ground-based in-situ measurements, satellite observations, and climate projections. The work is generally suitable for publication in Nature Geoscience. However, major revisions are needed for a clearer and more grounded presentation and interpretation of the results.

Major items:
- The authors repeatedly state that they are using a combined 60 years of harmonised aerosol observations. This statement is misleading as it suggests that they are dealing with a 60-year time series. In fact, I am missing a clear statement (or a table) as to which time periods are considered from which site. There's mentioning of 2010 to 2019, indicating that we are dealing with ten actual years of observations.
- The manuscript uses a lot of references. While this is to be expected when combining such different data sets as in this work, some references seem somewhat dates. I also cannot assess from the way references are formatted if we are not dealing with excessive self-citations as well.
- I have some difficulties with Figure 1 and the related analysis. First of all, it is really hard to recognise anything in those plots. If the work is focussed on March to August, panels a to g could be simplified to focus on these periods. I reckon the shading in those panels refers to the different sectors identified in the data analysis? In that context, I would challenge putting Tiksi and Utqiakvik, which are clearly affected by very different sources, in the same pot. Also, seasons are defined in the plot but not used in the text.
- It seems that the considered homogenised data set is restricted to 2010-2019. Clearly, some of the sites will have much longer time series. Could this be exploited to check in how far the predictions made with the method developed here hold when faced with past data? This would certainly make the work more convincing.
- The authors refer to the harmonised data set but it is not entirely clear what is meant with harmonisation. I suggest to move some details from the supplement (maybe Figure S6) to the methods section to walk the readers through the different steps of data analysis. A flowchart might help in that context.
- PotentialNPF-retrieval: Do the authors consider all aerosol types in the CALIPSO CCN climatology? Some of those might not be relevant for the Arctic boundary layer. Also, does the relationship assume that all CCN are related to NPF? It is stated earlier that long-range transport has a huge impact on aerosol concentrations in the Arctic.
- Interpretation of PotentialNPF: Does a value of 40 mean that NPF might occur in 2 out of 5 days or is it more abstract? Is there a minimum persistence of those events to classify a day as NPF day and to imply relevance for nCCN?
- The authors use the correlation between nCCN and PotentialNPF to come up with the concepts in Figure 4. I understand that the projection is based on future temperature. But from line 189 onwards, there is a discussion of the potential role of multiple processes in future particle concentrations. It is not clear how this complexity can be reconciled with the projection approach. Instead, one gets the impression that all this is pure speculation. In particular, the relationships underlining the projection approach are valid for a certain state of the system. But the Arctic will experience change that goes beyond higher temperature, such as sea-ice melt and the related exposure of new particle sources. Can the authors comment as to how their method will be valid if the considered system itself changes?
- The discussion needs to stick to what can be extracted with some fidelity from the findings. While the authors highlight the complexity of the Arctic system, they also take quite some liberty in the interpretation of their findings. As for the Abstract, large parts of the Discussion could have been written without much data analysis.

Minor items:
- Please check the tense of the text. I don't think past tense should be used throughout the manuscript but please confirm with the Editor for certainty.

- The title is catchy by unsuitable as the authors don't really provide a clear answer.
- The abstract could provide more quantitative findings. In its current form, it might have just as well been written without much of the presented research.
- line 48: I wouldn't refer to ACI as only parts of those interactions are mentioned. Given that the observations are in the Arctic, I would expect a statement regarding the relevance of aerosols for ice clouds.
- line 96: Not sure if causation can be implied from this observation without looking at air-mass history.
- line 131: It's not the entire Arctic when latitudes north of 82 degrees are excluded.
- line 135: It would be nice to know why exactly those parameters provide the necessary information. Also, which parameters are taken from the model for the projection of PotentialNPF?
- Figure 3: not clear how the error bars in those plots can be so small.
- line 263: What's hyper-local NPF?
- line 270 to 272: Not helpful without some idea of how the new observations will help.
- line 327: What is a daily surface play?
- line 329: Reference to quiet NPF days and the related citation seems unnecessary.
- line 337: It would be great if there was some motivation for this activity.
- line 348: Why 1500 m. This seems excessive for the Arctic boundary layer. In fact, https://doi.org/10.5194/acp-14-7869-2014 shows that reconciling in-situ measurements and active remote sensing is far from straightforward in the Arctic.

Reviewer #2

(Remarks to the Author)
Notes on Chua et al.
'Will climate change increase cloud condensation nuclei in the Arctic?'

The authors develop a new method to quantify the potential for new particle formation (Potential_NPF). By linking Potential_NPF derived at a limited number of sites to satellite-based H2O, UV, and aerosol extinction, they nicely extend the estimates to the broader Arctic region (65N-82N). Furthermore, by relating Potential_NPF to 2m temperature, they also project future changes in Potential_NPF (and CCN) under a warming scenario.

The topic is timely and highly relevant for climate science. However, I find that the methodology has several weaknesses that make it difficult to draw convincing conclusions. There are quite a few simplified assumptions along the whole chain, from deriving Potential_ NPF, to linking it to satellite variables, and then to projecting future changes, which raises concerns about the robustness of the results. While the study is interesting, I'm not fully convinced it meets the level of rigor expected for Nature Geoscience. See below for my main comments on the methodology.

Major comments:

The authors define a metric Potential_NPF to quantify the NPF probability accoring to \Delta N[12.5-15]. Given that this is a new metric, some evaluation against observations would be helpful, for instance by comparing it with the probability of observing banana-shaped events at different Potential_NPF values.

Overall, the method for deriving Potential_ NPF seems to rely on quite a few assumptions that are not well jutisfied:

- The authors fit five Gaussian KDE modes to n_z and treat the mode with the largest n_z as the NPF mode (5). While the banana-shaped pattern is visible, it seems quite possible that non-NPF events are still included in mode 5, especially given the long left tail extending toward zero. At the same time, mode 4 might also include a fair amount of NPF events, since its right tail overlaps with (or even exceeds) the mean of mode 5. If you check the high end of mode 4 (identified as non-NPF mode), you'd probably see a similar banana-like shape as well. As such, the definition of the NPF mode may be somewhat questionable.

- The assumption of a normal distribution for n_z across all sites also looks a bit too strong. From Fig. S6, the distributions (e.g., Zeppelin and Tiksi) do not appear to be normally distributed, which makes this step also somewhat questionable.

- The last step - linking Potential_ NPF to satellite-derived UV, H2O, and extinction using a regression - also seems a bit weak. For the study region (65°N < lat < 82°N), passive satellite retrievals (CERES in this case) are generally unreliable, which could bias the results. On top of that, the regression performance is not very strong ($R2\sim0.5$), and the errors are quite large (MAPE up to ~64%, even >80% at some sites). This might either indicates the impact of unreliabled retrievals or

missing processes in the regression model.

- The authors relate Potential_NPF based on UV, H2O, and extinction in the regression model, but only consider temperature in the future projections. Since H2O and extinction will also change, the resulting projections seem overly simplified. If the authors think T is improtant, why not include it in the regression model? The logic feels a bit self-contradictory

- With all this, the derivation of Potential_ NPF doesn't feel fully convincing for subsquent consclusions...

Abstract: The phrase "a combined total of 60 years of data" is somewhat misleading. Only after reading the Methods in more detail did I realise that this refers to the total volume of data across all sites, rather than the actual time period covered. The dataset is accutally from 2010-2019, is that right? Please clarify this in the main text and avoid using the phrase "60 years of data", as it may be very misleading.

Minor comments:

Fig. 1 : The text in Fig. 1 is quite small and hard to read. In Fig. 1a, the range (12.5–15) also doesn't match what's written in the caption (12.5–25), so that might need checking. It would also help to mark the sites for different regimes in different colours, so it's easier to link the figure to the text.

Table S2 : Time evolution plots alongside Table S2 (perhaps in the SI) would be helpful to better visualize the trends from 2010 to 2019, compared to just listing numbers.
L315: mean (μ) at 0.5 -> 1.5(?)

L354–356: The description of the soft clustering is quite vague. Although not explicitly stated, I assume the intention is to match each satellite pixel to the parameterizations derived from the four sites. More details on the clustering method and weighting should be included in the Methods, not only in the SI, or at least clearly referenced there.

Reviewer #3

(Remarks to the Author)
The manuscript discusses the development of cloud condensation nuclei (CCN) in the Arctic using observational data from different stations, campaigns, and satellite data. Combining these different data sets in a harmonised way enables the authors to investigate the relationship of new particle formation (NPF) and CCN concentration in Arctic regions. For future projections, the authors use SSP 5-8.5 models to forecast the change of NPF and thus CCNs in a warmer climate. The authors develop a new measure for NPF, Potential_NPF, which they use to compare the capability for NPF events in satellite and model data. For the analysis, they distinguish three different Arctic regions, the High Arctic, the Maritime Arctic, and Continental Eurasia. They find that the development of NPF varies between the different regions. While it increases in the High Arctic, the signal is not as unambiguous in the other regions.
The manuscript is well written and the argumentation is comprehensible. Both observational and model data are used for the study. The methods are well explained in the method section and supplementary information (SI). Combining the different data sets gives an interesting and new perspective and allows for Arctic wide comparison of NPF and CCN concentration which leads to the conclusion that it is not trivial to forecast the development of CCN concentration in a warmer climate since it strongly depends on the specific region in the Arctic and the different processes taken into account.

I consider the manuscript suitable for being published in Nature Geoscience after the following points have been addressed:

Comments:

- Fig. 1: Please comment on the physical process of condensation sinks also in the main text and not only in the appendix.
- Line 69 ff: A brief description on the methodology for the harmonisation is missing here. It is described in the methods section and in the SI in more detail. However, I think it is important to have a short description here as well to interpret the results presented in the following. At least, there should be a reference to the explanation.
- Line 69 ff: A statement about temporal data coverage of the different stations is missing.
- Please add a comment about the used instruments used to collect the data at the different stations. The table in the SI is not very well connected to the text and can be easily missed.
- Fig. 1: I am not completely sure how you calculate deltaN. Can you please explain this bit more comprehensively?
- Line 146 ff and Fig. 2: Can you compare the values of Potential_NPF to other regions than the Arctic to get a better feeling for the numbers? For example, is 50 a high or a low value? Since it is a newly defined parameter, it is hard to get a feeling about the meaning of the specific values.
- Fig. 3 and Line 289 ff: I understand that you use the station data to compare them to the satellite derived Potential_NPF. However, in the Maritime Arctic there is no station. Do you use campaign data for this, e.g. MOSAiC? If so (and also in general for the used ship-borne data), please add a comment about the different temporal and spatial coverages compared to the stations and how this biases the results.

- Line 185 ff: Relate this statement more concretely to the figure.
- Fig. 4: I like the figure. What do the grey circles stand for? Add an explanation to the caption.
- Line 207 ff: How good are the models at all in simulating NPF and CCNs, especially in polar regions? There should be a comment about the expected ability of the models to capture the studied processes, since I think, it can be questioned that they do a very good job here. How do the models compare to observations looking at simulations without extra warming? Also, the models do not capture Arctic amplification correctly. So how can they be trusted here?
- Line 390: Can you relate this to the main text? Where do you use the temperatures explicitly? If you use it for the computation/comparison of the Potential_NPF, please comment above in the main text.
- Can you include a plot of the temporal evolution of the Potential_NPF and NPF-CCN relationship during the measurement period of the stations? I think, it would be a good addition to the future model prediction. Can you already recognise the trend shown in the models? Also, how does the trend in the satellite data look like, e.g. relating to Fig. S1?
- SI Line 42 and Fig. S7: Is Z_score the same as n_z? Can you explain a bit more?
- SI Line 52: I am a bit confused about mu and sigma you use, also in the main text. Are you using the same mean and variance for all stations or is this the result of a fit? Did you calculate the numbers or did you get them from somewhere?

Technical comments:

- Line 52: Typo. I guess you mean „at times"
- Line 247: Check sentence. Do you mean „than local NPF potential"?
- Line 339/344: Introduce the abbreviations UV and [H20] when they are used for the first time in line 339.
- SI Line 108/109: Typo. Either "with increasing…" or "as Potential increases".
- Line 208: I would suggest to add „The" before „Table" in the caption of S8.

Version 1:

Reviewer comments:

Reviewer #1

(Remarks to the Author)
Thank you for considering my comments and for revising the manuscript accordingly. The work is much improved thanks to the added details and revised descriptions.
There is still one major issue, though, which is about the use of the high emission scenario SSP5-8.5 in the presentation of the findings an their discussion. As this scenario was recently dismissed by the scientific community as unrealistic, I suggest that any results related to it are omitted from the paper and that the work should focus on findings related to SSP2-4.5 instead.

Reviewer #2

(Remarks to the Author)
Thank you to the authors for addressing my comments. They took my concerns seriously and made substantial improvements to the manuscript. In particular, they evaluated the PotentialNPF metric against observations and significantly improved its estimation using a machine learning approach with additional predictors, including temperature.

The results and conclusions are now much more robust, and provide valuable insights into future CCN changes associated with NPF. I am very happy to give the manuscript a green light for publication.

Reviewer #3

(Remarks to the Author)
The manuscript has been revised according to the suggestions of all three reviewers. I think the changes improve the argumentation structure and comprehensibility and reduce the number of unclear formulations.
Regarding statistics, box plots present the distributions, and error bars are included where appropriate. Uncertainties are also mentioned in the text.
I consider the manuscript suitable for publication in Nature Geoscience and do not need to see a further revision.

I have the following minor points and typos to correct:

L. 32: I believe you mean the Maritime Arctic, i.e., use "the" instead of "and."
L. 104: Remove the colon.
L. 122: "at" appears twice in "where observed at only at a few sites"; please remove one.
L. 123 ff: Please explain the meaning of the brackets.
L. 164: Insert a comma before "too."
L. 169: How do you know that the air is "clean"? Please explain.
Fig. 3: Please write out the abbreviation "SS" — I assume it stands for supersaturation, but it was not introduced earlier.
Fig. 4: In the fourth line before "may increase" and "may decrease," please remove the dots for High Arctic and Maritime Arctic, as they appear to belong with the preceding line.
L. 403: Please italicize "PotentialNPF" and format "NPF" as a subscript/index.

Open Access This Peer Review File is licensed under a Creative Commons Attribution 4.0 International License, which permits use, sharing, adaptation, distribution and reproduction in any medium or format, as long as you give appropriate credit to the original author(s) and the source, provide a link to the Creative Commons license, and indicate if changes were made.
In cases where reviewers are anonymous, credit should be given to 'Anonymous Referee' and the source.
The images or other third party material in this Peer Review File are included in the article's Creative Commons license, unless indicated otherwise in a credit line to the material. If material is not included in the article's Creative Commons license and your intended use is not permitted by statutory regulation or exceeds the permitted use, you will need to obtain permission directly from the copyright holder.
To view a copy of this license, visit https://creativecommons.org/licenses/by/4.0/

General Response

We thank all three reviewers for their thorough and constructive engagement with our manuscript. In this revision, we have addressed all major and minor concerns. The most substantive changes include: (1) replacement of the original log-log multiple linear regression for $Potential_{NPF}$ with a machine-learning algorithm incorporating an expanded set of physically motivated predictors, substantially improving model performance from the previous R^2-value of 0.31 to the current 0.84 (full observational dataset); (2) explicit validation of $Potential_{NPF}$ against observed NPF variables; (3) an updated observational CCN dataset that focus on selected aerosol type so as to draw a clearer link between NPF and CCN; which lead to (4) a restructured regime classification with revised naming and clearer estimation of NPF and CCN under future temperature change in the Arctic. All changes are described in detail below. Original comments are in blue while text quoted from the revised manuscripts is indicated in italics.

Reviewer #1

The authors present an intriguing method for estimating future Arctic CCN concentrations based on a combination of ground-based in-situ measurements, satellite observations, and climate projections. The work is generally suitable for publication in Nature Geoscience. However, major revisions are needed for a clearer and more grounded presentation and interpretation of the results.

We thank the reviewer for the helpful comments. We have made several changes that we hope could improve the clarity of the methods and the derived conclusions. We appreciate the feedback.

The authors repeatedly state that they are using a combined 60 years of harmonised aerosol observations. This statement is misleading as it suggests that they are dealing with a 60-year time series. In fact, I am missing a clear statement (or a table) as to which time periods are considered from which site. There's mentioning of 2010 to 2019, indicating that we are dealing with ten actual years of observations.

We agree the original phrasing was misleading and apologise for the confusion. The "60 combined years" refers to the sum of valid observation-days across all stations (21,705 days in total, spanning data periods from 2003 to 2024), not a 60-year continuous record at any one site. The 2010–2019 period refers to the common 'window' with adequate data across multiple sites. that we used exclusively for trend analyses (Table S2). In fact, Table S4 had already listed the specific time periods of data availability at each observation location.

We have clarified this throughout the manuscript and added explicit temporal coverage per site in SI Table S4, which now lists the data period and number of valid data days for each station. Various edits were also made in the text to highlight the 2010-2019 'window' or the varying data periods. The confusing "60-year" phrasing has also been removed throughout the manuscript.

In the main manuscript:

> *"During a common 2010-2019 window, significant declines in accumulation-mode…"*

> *"From the common 2010-2019 window, Arctic-wide declines in CS…"*

In the methods:

"The final harmonized dataset comprised of a total of 21705 valid data days across eight different Arctic locations spanning 2003-2024, although data coverage periods varied (SI Table S4)."

In SI Text 1:

"...All in all, the final harmonized dataset comprised of a total of 21705 valid data days, although data coverage periods varied (SI Table S4)"

Caption for Table S1:

"…The duration of the datasets differed across stations and campaigns: Zeppelin (08/2010–10/2024), Villum (07/2010–09/2020), Tiksi (07/2010–10/2018), Pallas (01/2012–06/2021), Värriö (01/2003–06/2024), Alert (03/2011–12/2018), Utqiaġvik (10/2019–12/2024), Central Arctic (08/2018–06/2023)."

The manuscript uses a lot of references. While this is to be expected when combining such different data sets as in this work, some references seem somewhat dated. I also cannot assess from the way references are formatted if we are not dealing with excessive self-citations as well.

The references were formatted according to Nature's publishing style. We have reviewed and checked the reference list. Of the 145 references cited in the main manuscript, only eight are first-authored or corresponding-authored by the authors of the present study. Given the breadth of topics covered and the continuity in research development, it is inevitable that we have these self-citations. We seek the understanding here.

I have some difficulties with Figure 1 and the related analysis. First of all, it is really hard to recognise anything in those plots. If the work is focussed on March to August, panels a to g could be simplified to focus on these periods. I reckon the shading in those panels refers to the different sectors identified in the data analysis? In that context, I would challenge putting Tiksi and Utqiakvik, which are clearly affected by very different sources, in the same pot. Also, seasons are defined in the plot but not used in the text.

We thank the reviewer for the recommendations. The cosmetic shading that had coincidentally placed Tiksi and Utqiagvik together has been removed. We have retained all four seasons in Figure 1 as the December to February winter transport aka the 'Arctic haze' period is scientifically important for the full Arctic aerosol picture.

Figure 1 now looks:

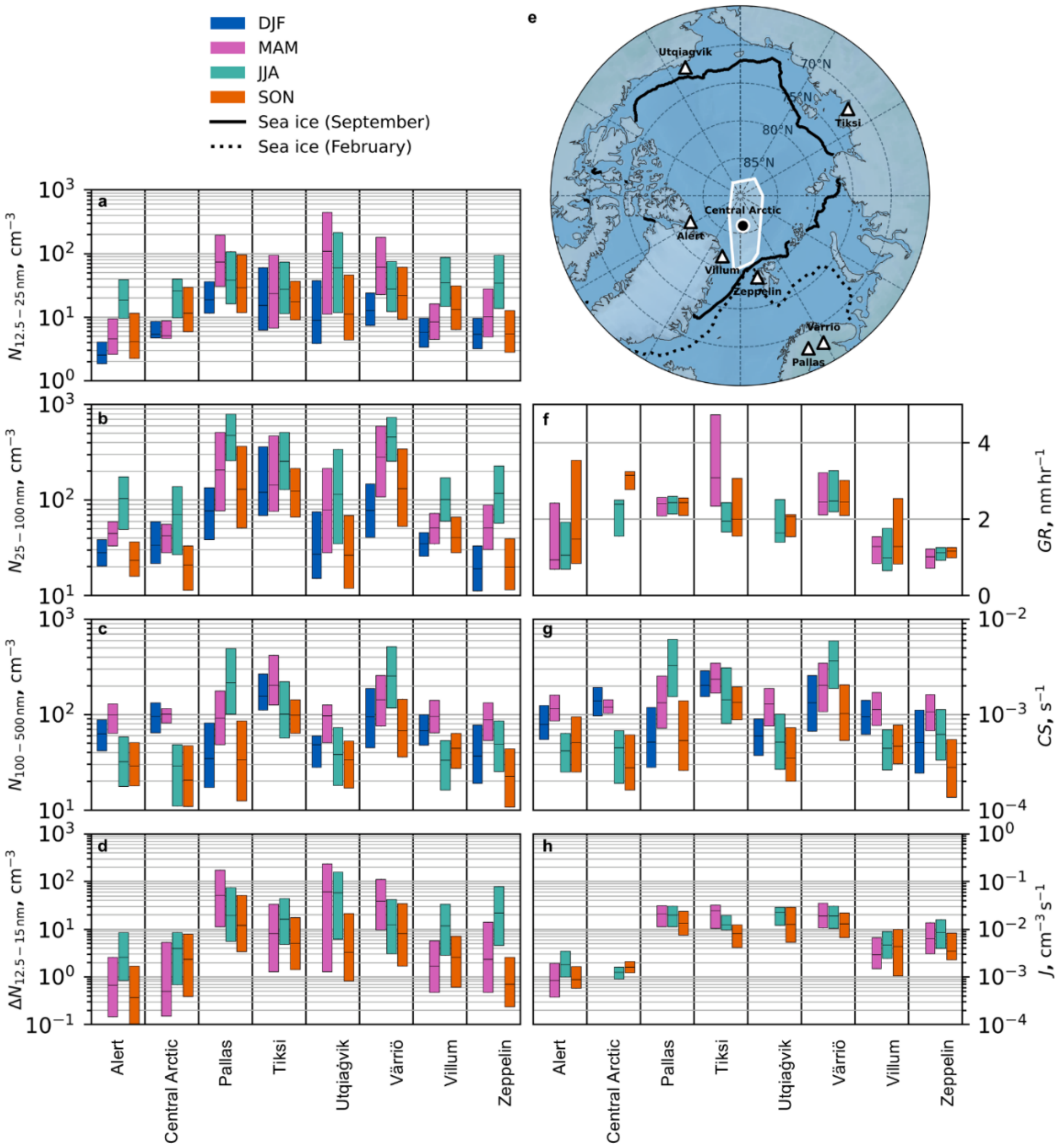

Figure 1. Aerosol parameters across eight Arctic locations (Alert, Central Arctic, Pallas, Tiksi, Utqiaġvik. Värrio, Villum, and Zeppelin) separated by traditional seasonal classification (December-February, March-May, June-August, and September-November) (a) Nucleation mode particles concentration: $N_{12.5\text{-}25\ nm}$; (b) Aitken mode particles concentration: $N_{25\text{-}100\ nm}$; (c) accumulation mode particles concentration: $N_{100\text{-}500\ nm}$; (d) diurnal variation in 12.5-15 nm particle concentration: $\Delta N_{12.5\text{-}15\ nm}$; (f) growth rate at 15-20 nm: GR; (g) condensation sink: CS; and (h) formation rate at 15 nm: J. Boxplots indicate the median and IQR of the parameters. (e) Map of various Arctic sites, a 'Central Arctic' polygon that shows the extent of the cruise campaigns, and maximum (February) and minimum (September) sea ice extent from 1981-2010 medians (Meier et al., 2024) .

It seems that the considered homogenised data set is restricted to 2010-2019. Clearly, some of the sites will have much longer time series. Could this be exploited to check in how far the predictions made with the method developed here hold when faced with past data? This would certainly make the work more convincing.

We apologise for the 2010-2019 confusion. Less the trend analyses (SI Table S2), all other observational aerosol records presented are from the full homogenised data set.

Regarding back testing, since the revised $Potential_{NPF}$ method is trained on observational data; therefore, a degree of agreement between observed and predicted values is expected. The time series of observed and predicted $Potential_{NPF}$ at each site show that the method reproduces the seasonal cycle (Figure R1). For a more quantitative assessment of performance, we refer to the error metrics provided in SI Text S2 and SI Figure S9. For reference, the model achieves an R^2 of 0.84 on the training dataset and an R^2 of 0.67 on the independent test site (Pallas), which was not used during model development.

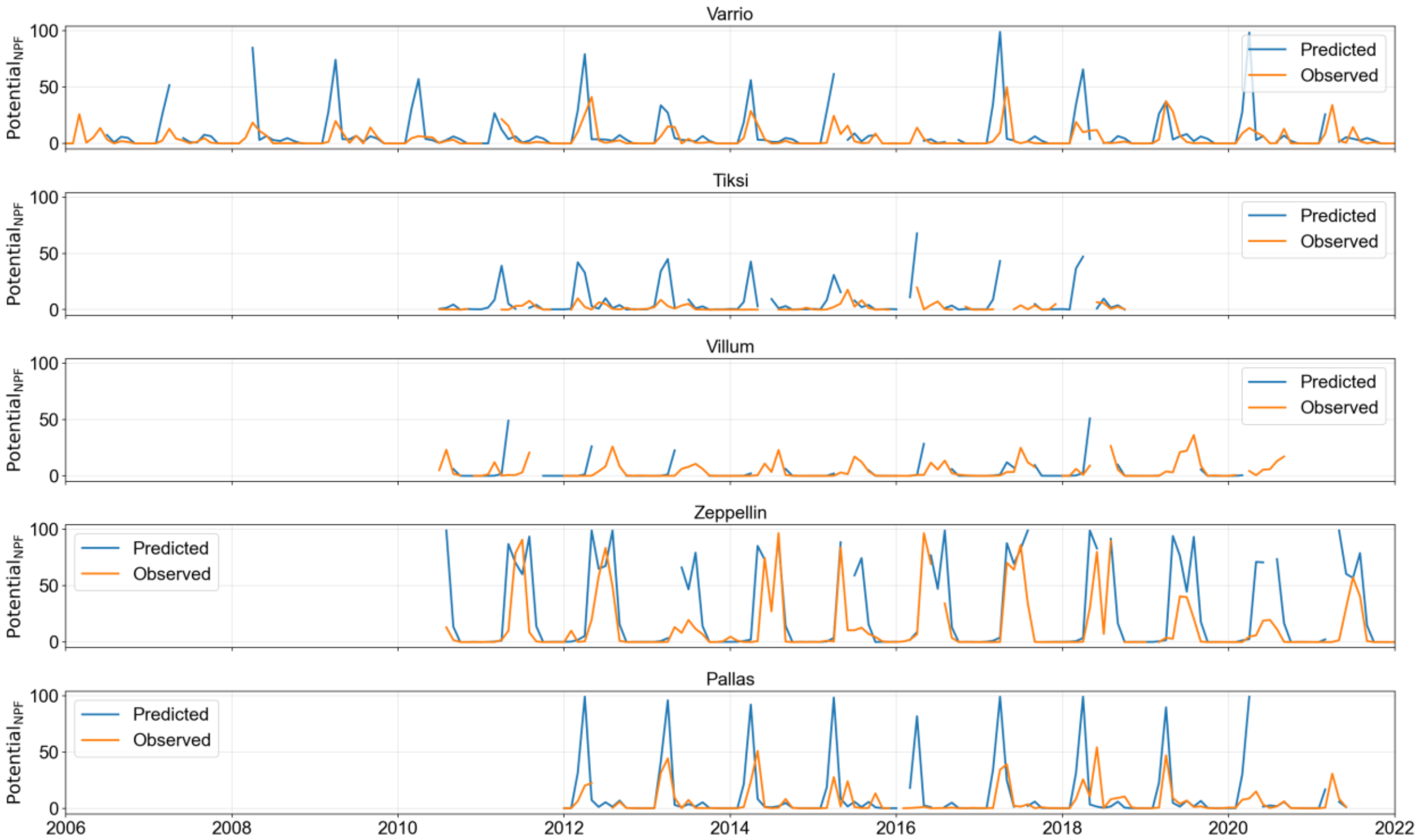

Figure R1: Timeseries of observed (orange) and predicted (blue) values of $Potential_{NPF}$ at each of the stations. Values were in monthly intervals during 2006-2022.

The authors refer to the harmonised data set but it is not entirely clear what is meant with harmonisation. I suggest to move some details from the supplement (maybe Figure S6) to the methods section to walk the readers through the different steps of data analysis. A flowchart might help in that context.

In our study, we refer to data harmonisation as the set of procedures used to make datasets collected across different sites comparable with one another. We appreciate the reviewer's suggestion regarding the inclusion of a flowchart. However, as the harmonisation procedures were not necessarily sequential and were often applied iteratively or independently, we considered that a flowchart may not accurately represent the workflow employed in this study.

Specifically, the harmonisation process involved several key steps:

1. Establishing common aerosol size-distribution cut-offs, which in this study were set to 12–500 nm. As different observational records may involve different instruments with varying detectable size ranges, applying a common size range, although potentially excluding information from larger or smaller particles, was necessary to ensure comparability across datasets.

2. Applying a unified methodology for deriving NPF-related parameters. Previously, the quantification of parameters such as NPF-event identification or particle growth rates could involve subjective visual fitting. In this work, algorithmic and objective approaches were used to minimise human bias and improve consistency across datasets.

3. Data quality check to ensure that data were consistent across the measurement period. Specific procedures differ by sites but were documented in the prior studies. The datasets used in this study were thus quality checked.

For Steps 1 and 2, additional details have now been included in the Methods section and SI Text S1. More detailed descriptions of the specific quality control procedures associated with Steps 3 are likely beyond the scope of the present manuscript. Instead, we have provided the original references in SI Table S4, where these procedures are documented in detail, so that interested readers may consult the relevant station-specific studies directly.

Methods now reads:

> *"The aerosol number size distributions were constrained to 12-500 nm to ensure comparability across observations. Site specific data inspection and quality-control procedures had been performed with more details on the specific procedures implemented at the individual observations documented in the corresponding references (Asmi et al., 2011, 2016; I. Beck et al., 2022; Boyer et al., 2023; Croft et al., 2016; Freud et al., 2017; Karlsson et al., 2022; Kyrö et al., 2014; Nguyen et al., 2016; Tunved et al., 2013; Väänänen et al., 2013)...*
>
> *...NPF relevant parameters, as part of the data harmonisation process, were computed using algorithmic objective methods(Aliaga et al., 2023; Kulmala et al., 2022; Lampilahti et al., 2025) to ensure inter-site comparability."*

SI Text 1 now reads:

> *"...Although some sites have particle size number distribution (PNSD) extending down to 3 nm-sized particles, the lower cutoff for our harmonized dataset was chosen to be approximately 12 nm, as that is the limit where there is quality-controlled data available across all measurement sites. For Villum, data during 2019-2020 were limited to <360 nm due to instrument limitations. Specific anomaly-detection and calibration procedures applied at each site were documented in the corresponding station references listed in SI Table S4...*
>
> *...Compared to traditional methods that could be subjective and/or time-consuming (Bhandari et al., 2025; Dal Maso et al., 2005), procedures to objectively and quickly quantify NPF events was applied in this study to ensure inter-site comparability."*

PotentialNPF-retrieval: Do the authors consider all aerosol types in the CALIPSO CCN climatology? Some of those might not be relevant for the Arctic boundary layer. Also, does the relationship assume that all CCN are related to NPF? It is stated earlier that long-range transport has a huge impact on aerosol concentrations in the Arctic.

Thank you for pointing this out and we concur with the reviewer that not all of the aerosol types are relevant for the purpose of our analysis. In this revision, we selected only CCN derived from clean continental and marine aerosol types (i.e. excluding polluted continental, elevated smoke, and dusty aerosol types). By focussing on these two aerosol types, we hope that the NPF-CCN relationships that we hypothesise would be more representative of local natural processes.

Separately, we have qualified our NPF-CCN relationship analysis by discussing only the degree to which NPF activity and CCN co-vary, not a claim that one is the sole source of the other. The sign and magnitude of this relationship are used as approximate indicators of the extent to which local NPF activity and CCN co-vary within different aerosol regimes. Weak or near-zero relationships are interpreted as indicative of stronger transport influence that is an important aerosol source in the Arctic. This clarification has now been stated explicitly in the revised text.

In the main text NPF potential section:

> *"The $Potential_{NPF}$ maps were overlaid atop a separately derived pan-Arctic dataset of CCN number concentration (N_{CCN}) (Choudhury & Tesche, 2023) and divided into aerosol regimes using a spatial clustering approach (Figure 3). As only CCN from clean continental and marine aerosols were considered, the N_{CCN} values presented here could be considered of clean natural background conditions…*
>
> *… To understand the extent to which local NPF affects CCN variability within each regime, the sign and magnitude of the N_{CCN} - $Potential_{NPF}$ slope provide useful, albeit approximate, indicators. A positive slope suggests that NPF activity and N_{CCN} are driven by the same local processes or even linked via a direct NPF-to-CCN pathway, whereas a zero or negative slope indicates a greater influence of aerosol transport or sources uncorrelated to NPF."*

In the methods:

> *"A height-resolved CCN dataset at 0.2% supersaturation level, calculated from a spaceborne lidar on board CALIPSO, was also acquired (Choudhury & Tesche, 2023). To focus only on the NPF-relevant CCN population, only CCN from clean continental and marine aerosols were considered…"*

Interpretation of PotentialNPF: Does a value of 40 mean that NPF might occur in 2 out of 5 days or is it more abstract? Is there a minimum persistence of those events to classify a day as NPF day and to imply relevance for nCCN?

We agree that the abstract number of $Potential_{NPF}$ could be hard to grasp. Justification of $Potential_{NPF}$ by showing its monotonic relationship with formation rates were added in the supplement. Additionally, the traditional Dal Maso (2005) classification was manually applied to the Värriö dataset, the longest dataset in our records. A rough 1:1 approximation could be made between the monthly percentage of days with Class I and Class II events and $Potential_{NPF}$ values (R^2=0.72). Therefore, for easy interpretation, $Potential_{NPF}$ could be understood as the percentage of days within that month during which NPF events are likely to occur.

The section in SI Text S1 reads:

"Importantly, $Potential_{NPF}$ should be interpreted as a probabilistic metric that reflects the position of the observed n_z value within the NPF-mode distribution. Despite this probabilistic formulation, $Potential_{NPF}$, when compared to formation rates of 15 nm-particles (J_{15}), showed that $Potential_{NPF}$ increased with increasing J_{15} (SI Figure S7b), justifying $Potential_{NPF}$ as an indicator for NPF intensity. At Värriö, the longest dataset in our records, monthly values of $Potential_{NPF}$ also correlated well in a 1:1 relationship with the monthly percentage of days with Class I and Class II events (classified according to Dal Maso et al. (2005)) (SI Figure S7c). Therefore, an approximation could be made, with care, that the monthly value of $Potential_{NPF}$ roughly corresponds to the percentage of days within that month during which NPF events are likely to occur."

SI Figure S7 looks:

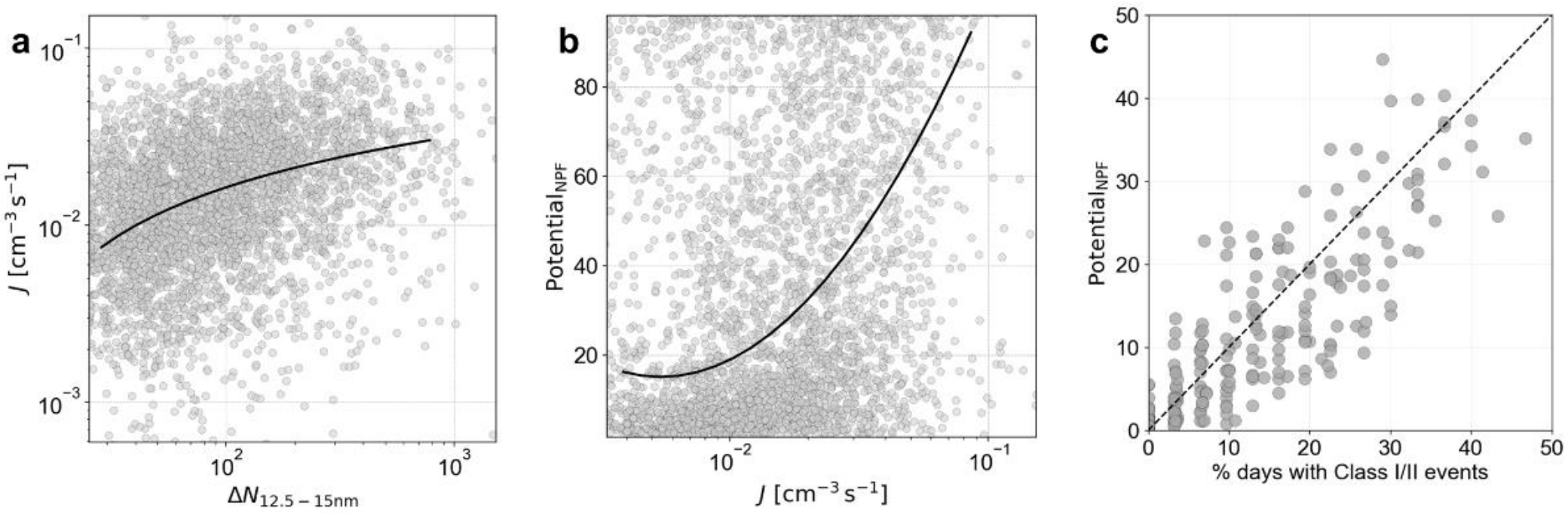

Figure S7. (a) Formation rates of 15 nm particles (J_{15}), against $\Delta N_{12.5-15}$ values, and (b) $Potential_{NPF}$ against formation rates of 15 nm particles (J_{15}) across all stations. The black lines indicate the median while the grey scatter indicates raw datapoints. (c) Monthly values of $Potential_{NPF}$ at Värriö against the corresponding monthly value of the percentage of days with Class I/II events (according to Dal Maso et al. (2005)) observed. Dashed line is the 1:1 ratio.

The authors use the correlation between nCCN and PotentialNPF to come up with the concepts in Figure 4. I understand that the projection is based on future temperature. But from line 189 onwards, there is a discussion of the potential role of multiple processes in future particle concentrations. It is not clear how this complexity can be reconciled with the projection approach. Instead, one gets the impression that all this is pure speculation. In particular, the relationships underlining the projection approach are valid for a certain state of the system. But the Arctic will experience change that goes beyond higher temperature, such as sea-ice melt and the related exposure of new particle sources. Can the authors comment as to how their method will be valid if the considered system itself changes?

We agree that future changes in $Potential_{NPF}$ and N_{CCN} are likely more complex than represented here, and we acknowledge that the original projection approach may have been overstated. In response, we have made several revisions to clarify and appropriately qualify our analysis.

First, we now frame these results as *temperature-sensitivity estimates* rather than absolute projections. This reflects the substantial uncertainties associated with future aerosol and precursor emissions, particularly the fate of transported anthropogenic aerosols.

Second, we have added a dedicated justification for using temperature as the primary parameterising variable in the main manuscript, rather than alternative predictors such as sea-ice concentration. A correlation analysis shows that temperature is correlated with the other variables in the parameterisation. Specifically, temperature is positively correlated with water vapour content ($[H_2O]$) and NDVI ($[NDVI]$), and negatively correlated with sea ice concentration ($[sea\ ice]$). Therefore, temperature- $Potential_{NPF}$ relationship indirectly captures the influence of multiple environmental factors, including precursor vapour availability and surface conditions, through these covariations. These are the changes we made:

In 'NPF potential and CCN under a warming Arctic' section of the manuscript:

> *"Given the strong correlations between air temperature and other environmental variables such as water vapour content or sea ice concentration (SI Figure S5), temperature changes could thus act as a physical marker of Arctic warming…"*

In SI Text S4:

> *"Since temperature change is a driving force or indicator in many Arctic processes, temperature change was regarded as the proxy for environmental changes in the Arctic (Box et al., 2019). Among the six predictors in the XGBoost model, surface air temperature is also the most reliably projected CMIP6 variable in the Arctic, whereas future projections of the other variables carry substantially greater inter-model uncertainty. Furthermore, temperature, consistent with its role as a co-varying driver of Arctic atmospheric and surface conditions, is found to be strongly correlated to other environmental indicators such as water vapour, sea ice and terrestrial vegetation (SI Figure S5), justifying temperature changes as a good indicator of Arctic warming…"*

SI Figure S5:

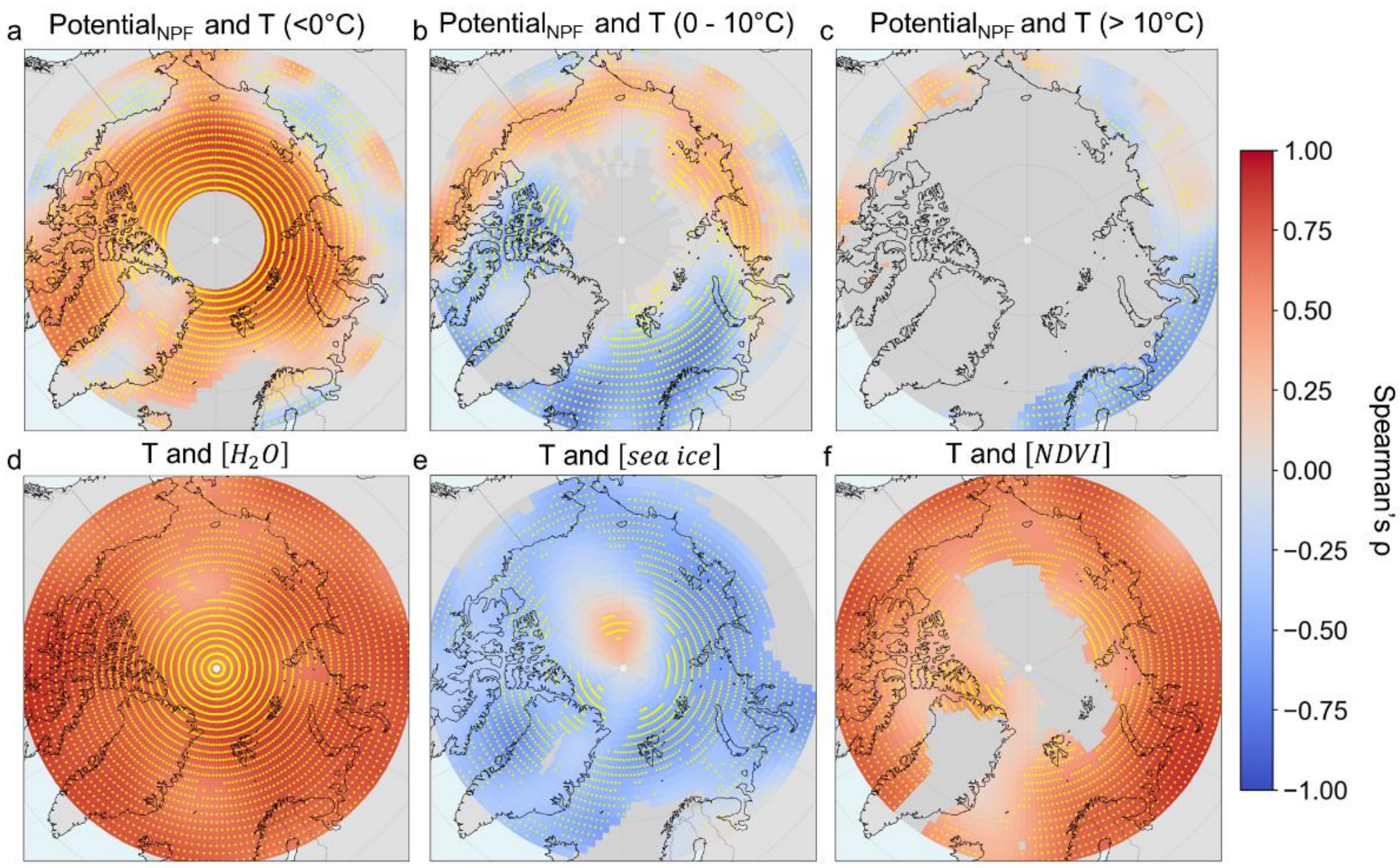

Figure S5. Spearman's ρ values between surface air temperature (T) and $Potential_{NPF}$ calculated at each pixel for data during 2006-2021 March-August at three temperature bins: (a) <0 °C, (b) 0-10 °C, and (c) >10 °C. For visualisation of the sensitivity of the satellite variables to T, Spearman's ρ values were calculated at each pixel for data during 2006-2021 March-August between surface air temperature (T) and (d) water vapour content ($[H_2O]$), (e) sea ice concentration ($[sea\ ice]$), and (f) Normalised Difference Vegetation Index ($[NDVI]$). The values of $[NDVI]$ and $[sea\ ice]$ at each pixel were computed as the spatial mean across a 500 km buffer. Pixels where ρ value is statistically significant ($p < 0.1$) are marked by yellow dots.

The discussion needs to stick to what can be extracted with some fidelity from the findings. While the authors highlight the complexity of the Arctic system, they also take quite some liberty in the interpretation of their findings. As for the Abstract, large parts of the Discussion could have been written without much data analysis.

In revising the manuscript, we have reassessed the Discussion section to ensure that the interpretations are more clearly linked to the results and that speculative statements are appropriately moderated. Following the journal's style, much of the interpretation and contextual discussion associated with the findings was integrated directly into the earlier sections ("Latitudinal and seasonal gradients…", "NPF potential…", and "NPF potential and CCN…"), where the observational analyses are presented alongside their interpretation. Consequently, the final Discussion section was intended primarily to synthesise the broader implications, limitations, future directions and to conclude the study.

The Discussion section was thus organised around three paragraphs: (1) the broader contribution of this study relative to previous Arctic aerosol studies, (2) limitations associated with the methodologies and interpretations drawn in this study and (3) priorities for future work and closing statement. In particular, the second paragraph were included specifically to acknowledge important limitations in both the methodology and interpretation. These sections were included to appropriately qualify the interpretation of the findings and to avoid overinterpretation of the results. Overly speculative statements were also removed or moderated. Please refer to the updated Discussion section.

Please check the tense of the text. I don't think past tense should be used throughout the manuscript but please confirm with the Editor for certainty.

We have used the tenses according to what we considered most appropriate for the context of each clause. We would be grateful for any suggested corrections or improvements.

The title is catchy by unsuitable as the authors don't really provide a clear answer.

We intended the title to be interrogative as it challenges common assumptions that that Arctic trends in CCN are monotonic as we write in the conclusion "… caution that simple narratives – such as 'warming will increase NPF and CCN across the Arctic'- risk being misleading". At the same time, we thought that such a style might capture the general readership of *Nature Geoscience*.

We acknowledge the reviewer's concern and instead of waiting until the Discussion to provide the answer, the answer is now immediately thereafter in the revised abstract:

> "*CCN concentrations associated with NPF activity could increase by 2.8– 13% in the High Arctic, but decline by 4.5– 22% in the Maritime Arctic.*"

Nonetheless, we shall follow any judgement of the editor on the title.

The abstract could provide more quantitative findings. In its current form, it might have just as well been written without much of the presented research.

We agree and have rewritten the abstract. The revised abstract now cites specific figures.

Abstract reads:

> *"In the rapidly warming Arctic, aerosol–cloud interactions remain one of the key uncertainties in predicting whether clouds will amplify or buffer ongoing warming. A part of this uncertainty is new particle formation (NPF): a key source of aerosol particles that can grow into cloud condensation nuclei (CCN). Here, harmonized field aerosol observations across the Arctic spanning 2003-2024, alongside satellite-derived data, were analysed to quantify NPF trends and estimate their climatic implications across the Arctic region. Three distinct aerosol regimes with contrasting NPF–CCN relationships were identified across the Arctic: the locally dominated High Arctic and Maritime Arctic, and the transport-dominated Continental Arctic. Temperature-sensitivity estimates based on future warming scenarios (SSP 5-8.5) suggests that by 2050-2060, CCN concentrations associated with NPF activity could increase by 2.8– 13% in the High Arctic, but decline by 4.5– 22% in the Maritime Arctic. Inasmuch as these divergent projections show that Arctic aerosol–cloud feedbacks will evolve differently, the mosaic of competing aerosol regimes must hence be captured to better understand the changing Arctic."*

line 48: I wouldn't refer to ACI as only parts of those interactions are mentioned. Given that the observations are in the Arctic, I would expect a statement regarding the relevance of aerosols for ice clouds.

Revised. Sentence now reads:

> *"Aerosol–cloud interactions, including the role of aerosol particles as cloud condensation nuclei (CCN) for liquid clouds and as ice-nucleating particles (INP) for ice and mixed-phase clouds, are an important but insufficiently represented part of the Arctic climate system(Prenni et al., 2007; Schmale et al., 2021; Stevens et al., 2018). Here, this paper focuses on CCN, as new particle formation (NPF) is an important local source of CCN in the Arctic."*

line 96: Not sure if causation can be implied from this observation without looking at air-mass history.

While air-mass trajectory or transport analyses were not explicitly performed in this study, previous studies have conducted similar analyses and demonstrated that accumulation-mode aerosols in the Arctic can be strongly influenced by Eurasian or North American emissions transported into the region. These studies are cited in support of the statement. In the revised manuscript, the wording

has also been softened to indicate a possible contribution rather than implying direct or definitive causation.

Sentence reads:

> *"… one possible reason could be the reduction in anthropogenic emissions across Eurasia and North America transported to the Arctic, either at source or due to changing atmospheric patterns (Bourgeois & Bey, 2011; Breider et al., 2017; Heslin-Rees et al., 2024; Mackie et al., 2016; Pernov et al., 2022; Schmale et al., 2022)."*

line 131: It's not the entire Arctic when latitudes north of 82 degrees are excluded.

We removed the "entire Arctic" phrasing throughout the manuscript. Sentence now reads:

> *"To address these limitations, we developed a novel metric, $Potential_{NPF}$, and used it to derive a 15-year (2006-2021) dataset of monthly maps spanning the Arctic from 65°N to 83°N (Figure 2 and SI Figure S2)."*

line 135: It would be nice to know why exactly those parameters provide the necessary information. Also, which parameters are taken from the model for the projection of PotentialNPF?

The choice of parameters was guided by a priori parameterisations of nucleation-mode particle formation presented in previous studies (Kulmala et al., 2011; Petäjä et al., 2009; Sundström et al., 2015), in which NPF activity was expressed as a function of photochemistry, meteorological conditions, aerosol sinks, and precursor vapour availability. These relationships were merely translated into a mathematical framework as presented in Eq. 4. We have now added a brief justification in the main text, together with more detailed explanations in SI Text S2.

Text now reads:

> *"$Potential_{NPF}$ is derived from monthly satellite-based environmental variables, including ultraviolet radiation (UV), water vapour content ($[H_2O]$), atmospheric aerosol loading (σ_{ext}), vegetation indices ($[NDVI]$), and sea-ice concentration ($[sea\ ice]$), together with surface air temperature (T) obtained from a reanalysis dataset. These predictors were selected because they represent proxies of environmental conditions affecting NPF processes, including photochemistry (UV), meteorological conditions ($[H_2O]$, T), aerosol sinks (σ_{ext}), and precursor vapour availability ($[NDVI]$, $[sea\ ice]$)."*

For the estimation of $Potential_{NPF}$ based on temperature changes by 2050-2060, the only parameter that we used is surface air temperature.

Figure 3: not clear how the error bars in those plots can be so small.

The relatively small error bars reflect the 95% confidence intervals (CIs) of the area-weighted median, estimated using 10,000 bootstrap iterations. As the statistics were derived from 15 years of gridded observations, the uncertainty associated with the median estimate can become relatively small when the underlying spatial patterns are consistent. Since our primary objective here is to quantify the precision of the regime-average estimates, we though that perhaps confidence intervals are appropriate as an indication of the robustness of the median estimates. To improve clarity, we

have now explicitly stated in the Figure 3 caption that the error bars represent bootstrap-derived 95% confidence intervals of the area-weighted median.

Caption reads:

> *"Error bars in (b) - (d) represent the 95% bootstrap confidence interval around the area-weighted median estimated from 10,000 bootstrap iterations."*

line 263: What's hyper-local NPF?

Reworded to "local".

line 270 to 272: Not helpful without some idea of how the new observations will help.

Sentence now reads:

> *"Adding to the complexity, the current temperature dependence of $Potential_{NPF}$ might also evolve as NPF-related emission sources and the seasonality of their release changes (L. J. Beck et al., 2021; Chang et al., 2011; Dall´Osto et al., 2018; Tham et al., 2023) – a reality already in the rapidly-changing marginal ice zone of the High Arctic"*

line 327: What is a daily surface play?

Line has been removed in this revised manuscript

line 329: Reference to quiet NPF days and the related citation seems unnecessary.

We retain the reference to Kulmala et al. (2022) as the normalisation step the study pioneered is important to our harmonisation procedure. Without scaling minimum and maximum values along each time bin, low-intensity NPF events – which are characteristic of the Arctic as compared to boreal forest environments – would be invisible in the aggregated surface plots and excluded from our analysis, leading to systematic underestimation of NPF occurrence. The citation therefore directly justifies a methodological choice.

line 337: It would be great if there was some motivation for this activity.

We have added a brief justification for the selection of the proxy predictors, including the environmental processes that they are intended to represent. Additional details and rationale for each predictor are now also provided in SI Text S2.

Section now reads:

> *"The dependent variable was n_z while six proxy predictors were included, namely UV radiation (UV), air temperature (T), water vapour content ($[H_2O]$), atmospheric aerosol load (σ_{ext}), terrestrial precursor vapours ($[NDVI]$) using NDVI (Normalised Difference Vegetation Index) as the proxy, and marine precursor vapours ($[sea\ ice]$) with sea ice concentration used as a proxy, as expressed in Eq. 4. The choice of the predictors is further explained in SI Text S2."*

In SI Text 2:

"NPF intensity, expressed through formation rate, has been parametrized using auxiliary variables such as global radiation, precursor gas concentration, relative humidity and condensation sink (Dada et al., 2020; Heintzenberg et al., 2017; Li et al., 2025; Petäjä et al., 2009). Given the Arctic setting of this study, the terrestrial precursor vapours are likely dominated by highly oxygenated organic molecules (HOMs) associated with terrestrial biogenic emissions(L. J. Beck et al., 2021; Jokinen et al., 2022), while marine precursor vapours are likely composed of iodine- and sulfur-containing compounds linked to open waters and processes associated with sea-ice formation and breakup (L. J. Beck et al., 2021; Boyer et al., 2024; Dall´Osto et al., 2017, 2018; He et al., 2021; Kecorius et al., 2019; Kerminen et al., 2018; Pernov et al., 2024). Since these precursor vapours cannot be directly observed from satellite measurements, indicators of their source regions, namely NDVI and sea-ice concentration, were thereby used as proxies for their abundance."

line 348: Why 1500 m. This seems excessive for the Arctic boundary layer. In fact, https://doi.org/10.5194/acp-14-7869-2014 shows that reconciling in-situ measurements and active remote sensing is far from straightforward in the Arctic.

Indeed, we fully agree and acknowledge that reconciling CALIPSO extinction retrievals with in-situ measurements in the Arctic is non-trivial, as pointed out in (Tesche et al., 2014). In this revised manuscript, we have replaced the simplistic log-log multiple linear regression between variables with an XGBoost tree-based model, which captures non-linear interactions between predictors better. The feature importance analysis (SHAP values, SI Figure S9c) identified $[H_2O]$ as the second most important predictor of n_z, with σ_{ext} ranking third. Their relatively high importance suggests that the model could have learned to use $[H_2O]$ partly as a covariate for σ_{ext}, which was what had been sugggested in Tesche et al. (2014).

Regarding the 1500 m integration limit, our aim is to capture the total aerosol column load rather than the boundary layer concentration. CALIPSO extinction profiles in the Arctic show values decaying toward zero between 1–2 km (Yao et al., 2023), so integration to 1500 m captures the bulk of the column under most conditions. If the true boundary layer were shallower, the additional integrated layers would contribute near-zero extinction values with negligible impact on the total load estimate. As Arctic boundary layer identification from reanalysis remains uncertain (Xi et al., 2024), we avoided using ERA5-derived boundary layer heights as the upper limit so that we avoid another source of uncertainty if possible. Comparable column-integration approaches to 2000 m has been used for CALIPSO-derived aerosol extinction in other polar studies (Dasarathy et al., 2021, 2023), providing methodological precedent.

Reviewer #2

The authors develop a new method to quantify the potential for new particle formation (Potential_NPF). By linking Potential_NPF derived at a limited number of sites to satellite-based H2O, UV, and aerosol extinction, they nicely extend the estimates to the broader Arctic region (65N-82N). Furthermore, by relating Potential_NPF to 2m temperature, they also project future changes in Potential_NPF (and CCN) under a warming scenario.

The topic is timely and highly relevant for climate science. However, I find that the methodology has several weaknesses that make it difficult to draw convincing conclusions. There are quite a few simplified assumptions along the whole chain, from deriving Potential_ NPF, to linking it to satellite variables, and then to projecting future changes, which raises concerns about the robustness of the results. While the study is interesting, I'm not fully convinced it meets the level of rigor expected for Nature Geoscience. See below for my main comments on the methodology.

We thank the reviewer for the constructive assessment of our study. In response to the reviewer's comments, we have revised several aspects of the methodology, even adopting a new estimation model. We feel that these revisions have strengthened the robustness of the analysis and in some cases, led to more nuanced conclusions. We believe that the revised manuscript now presents a clearer and more rigorously constrained assessment of Arctic NPF and its possible relationship with CCN variability.

The authors define a metric Potential_NPF to quantify the NPF probability according to ΔN[12.5-15]. Given that this is a new metric, some evaluation against observations would be helpful, for instance by comparing it with the probability of observing banana-shaped events at different Potential_NPF values.

We agree and have made additional evaluation steps to better interprete the $Potential_{NPF}$ values. When plotted against formation rates of 15 nm-particles (J_{15}), a trend of $Potential_{NPF}$ increasing with rising J_{15} was observed, justifying the interpretation of $Potential_{NPF}$ as an indicator of NPF intensity. For a practical test, we laboriously applied the traditional Dal Maso (2005) classification to the Värriö dataset, the longest dataset in our records. We found that monthly values of $Potential_{NPF}$ correlated well in a roughly 1:1 relationship (R^2=0.72) with the monthly percentage of days with Class I and Class II events. Correspondingly, a practical way to conceptualise the $Potential_{NPF}$ values is to use it as a rough approximation of the percentage of days within that month during which NPF events are likely to occur. These evaluation tests are added into the revised manuscript.

In SI Text S1:

> *"Importantly, $Potential_{NPF}$ should be interpreted as a probabilistic metric that reflects the position of the observed n_z value within the NPF-mode distribution. Despite this probabilistic formulation, $Potential_{NPF}$, when compared to formation rates of 15 nm-particles (J_{15}), showed that $Potential_{NPF}$ increased with increasing J_{15} (SI Figure S7b), justifying $Potential_{NPF}$ as an indicator for NPF intensity. At Värriö, the longest dataset in our records, monthly values of $Potential_{NPF}$ also correlated well in a 1:1 relationship with the monthly percentage of days with Class I and Class II events (classified according to Dal Maso et al. (2005)) (SI Figure S7c). Therefore, an approximation could be made,*

with care, that the monthly value of $Potential_{NPF}$ roughly corresponds to the percentage of days within that month during which NPF events are likely to occur."

SI Figure S7 looks:

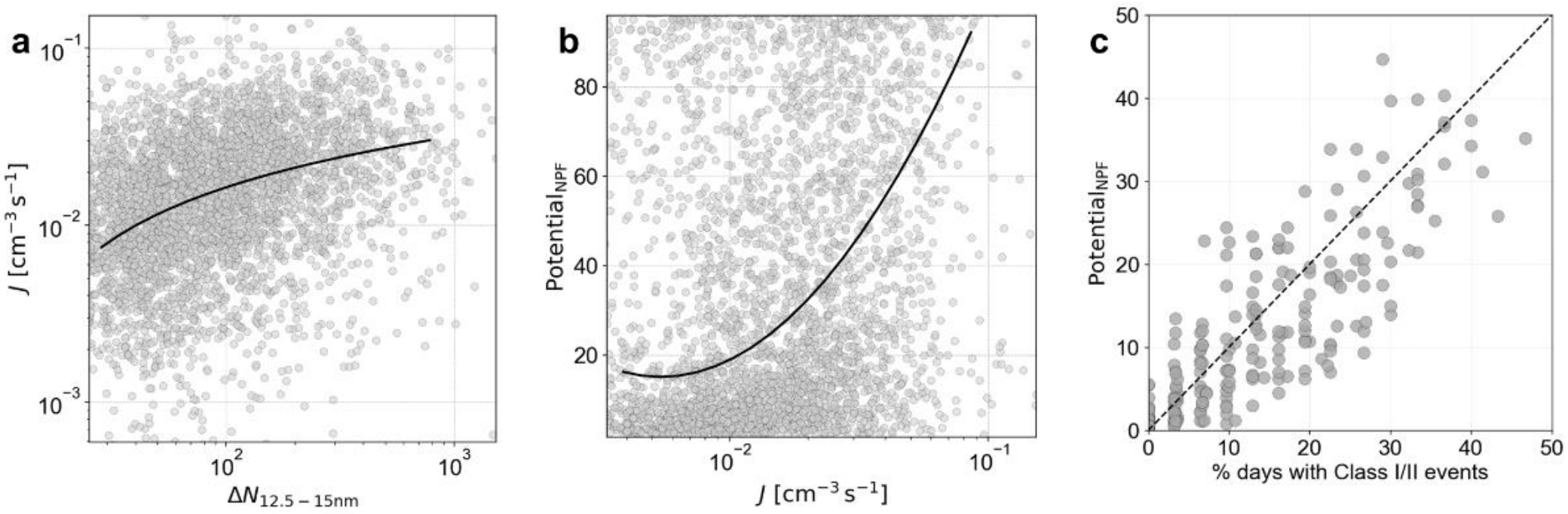

Figure S7. (a) Formation rates of 15 nm particles (J_{15}), against $\Delta N_{12.5-15}$ values, and (b) $Potential_{NPF}$ against formation rates of 15 nm particles (J_{15}) across all stations. The black lines indicate the median while the grey scatter indicates raw datapoints. (c) Monthly values of $Potential_{NPF}$ at Värriö against the corresponding monthly value of the percentage of days with Class I/II events (according to Dal Maso et al. (2005)) observed. Dashed line is the 1:1 ratio.

Overall, the method for deriving Potential_ NPF seems to rely on quite a few assumptions that are not well jutisfied:

We shall address these assumptions one-by-one in the following responses

The authors fit five Gaussian KDE modes to n_z and treat the mode with the largest n_z as the NPF mode (5). While the banana-shaped pattern is visible, it seems quite possible that non-NPF events are still included in mode 5, especially given the long left tail extending toward zero. At the same time, mode 4 might also include a fair amount of NPF events, since its right tail overlaps with (or even exceeds) the mean of mode 5.

We apologise for the unclear explanation.

The principle behind the KDE mode fits is to provide a categorical interpretation for n_z. What does a value of n_z =1 mean? As documented in (Aliaga et al., 2023) Section 2.1.2, a distribution of n_z, which is an indicator of NPF intensity, can be used to identify any NPF mode(s). Thus, higher values of n_z, implying higher NPF intensity, would more likely belong to the NPF mode(s) and vice versa.

We then tried to assign a mode to each daily value of n_z, for the only purpose of able to plot a 'average' surface daily plot to elucidate the bananas in Figure S6. The nature of fitting 5 Gaussian KDE modes to the density distribution means that at each value of n_z, there is differing mode contribution. For example at n_z=1, it might be Mode 1 = 1%, Mode 2 =2%. Mode 3 =3%, Mode 4 =30%, Mode 5 = 64%. Therefore, the Mode with the highest percentage contribution, in the case of our example here Mode 5 would be assigned to the particular value of n_z. Again, this assignment is for the visualisation of the banana plots in Figure S6.

We found that 5 modes sufficiently replicated the distribution across all sites, and serendipitously, with clear NPF events bananas observed in the Mode 5 plots, leading us to label it as the 'NPF mode'. Moving to a situation of a blind location, that is the purpose of our satellite-based estimation of $Potential_{NPF}$, we assume that the n_z values there follow a normal distribution (addressed in a later response below) and that the rightmost mode, or Mode 5, is the NPF mode. Back to our example, if this blind station has a value of n_z=1, the corresponding Mode 5 contribution is 23.5%, that we define as $Potential_{NPF}$ = 23.5, as expressed in Eq. 1b too.

Based on this logic, it is not a case whether an observation belongs to Mode 4 or Mode 5 with a categorical outcome. Instead, we provide a Bayesian probabilistic value of an observation value belonging to Mode 5, or for easy layman's interpretation, the probability of an NPF event occurring at a certain value of n_z.

Going back to the original comment, we agree that certain NPF event might occur in the Mode 4 value domain as in Figure S6 and vice versa. However, in subsequent calculation of $Potential_{NPF}$, due to its probabilistic nature, we do not make the sharp binary cut between modes.

For clarification, Figure R2 below shows the median surface daily plots (normalized) for the five fitted modes at Tiksi and Villum. We can thus ascertain that the median daily value in Mode 1-4 are not characteristic of a NPF event day, with only the median daily value in Mode 5 characteristic of the 'banana' NPF day. We also edited the methods to improve the clarity of this process.

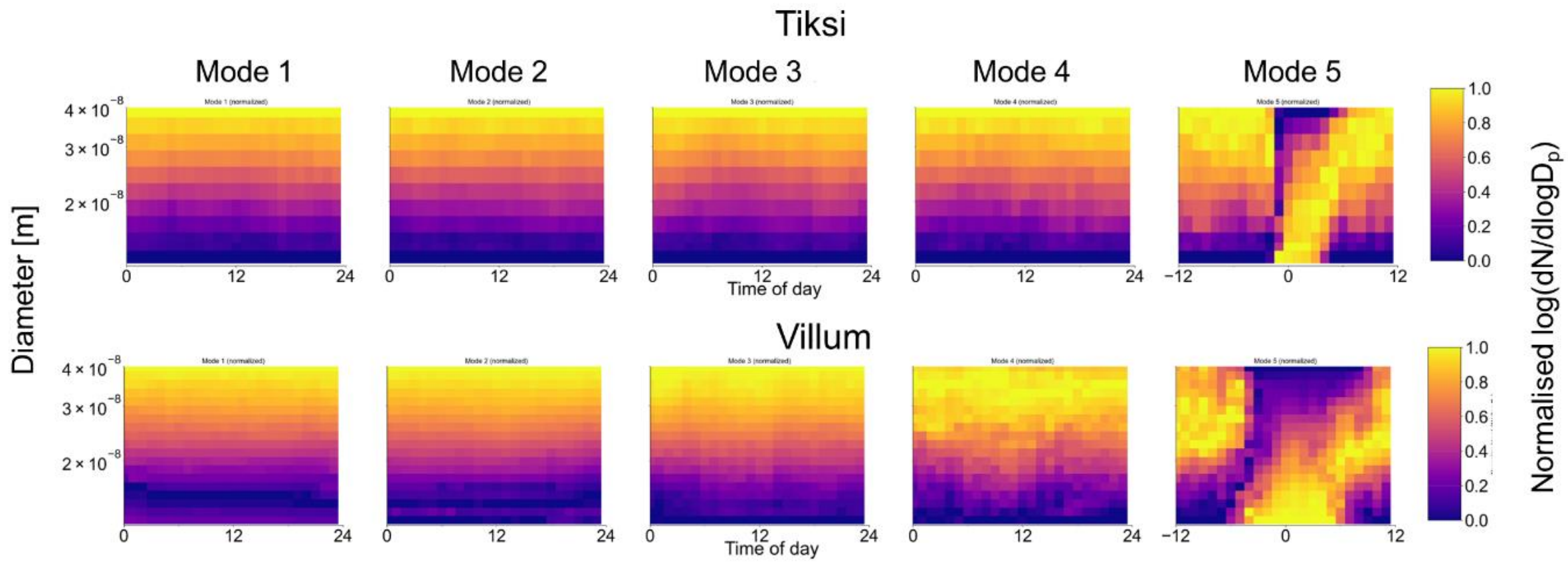

Figure R2. Median surface daily plots of the days under the various modes normalized across each time segment (vertically) and aligning the peak values of the 12.5nm size bin to 0 hour.

In Methods:

> *"For each observational site, density histograms of n_z were fitted with Kernel Density Estimator (KDE) curves, with five curves sufficiently reproducing the distribution at each site. Among these modes, only the aggregated normalised daily surface plots of $dN/d\ logD_p$ for Mode 5, the mode with the highest central values, consistently exhibited the characteristic "NPF banana" pattern associated with particle growth events (Figure S6). Accordingly, Mode 5 was visually identified as the "NPF mode". This visual categorisation was performed primarily for comparison with historical approaches, in which NPF-event days were traditionally identified through manual classification (Aliaga et al., 2023; Dal Maso et al., 2005)."*

The assumption of a normal distribution for n_z across all sites also looks a bit too strong. From Fig. S6, the distributions (e.g., Zeppelin and Tiksi) do not appear to be normally distributed, which makes this step also somewhat questionable.

We apologise if we were unclear. The assumption of a normal distribution was applied to an unseen site/grid, and not directly on the observational dataset per se. The reason why the observational dataset might not appear normally distributed visually reflect the uneven temporal sampling and site-specific conditions that may skew the dataset. For example, a station with more datapoints collected during the summer season when NPF activity is higher might have the n_z distribution more skewed to higher values. To our objective of identifying $Potential_{NPF}$ for an unknown pixel, the issue of uneven sampling would not apply.

Nonetheless, as a proof of concept, we tested the effect of increasing sample size at each of the station and its effect on the Shapiro-Wilk W statistic, a test of normality where W=1 imply that the sample data matches a perfect normal distribution. Across all stations, increasing the size of the bootstrap sample also led to increases in the W statistics towards 1 (Figure R3), following the Central Limit Theorem that a sample with sufficiently large number of datapoints will approach a normal distribution. This pattern supports our assumption that for an unknown pixel with enough datapoints, the n_z distribution would also follow a normal distribution.

We also tested the normality of the n_z values at each of the stations in Figure S6 (Värriö, Tiksi, Villum, Zeppelin, Pallas) and all of them have Shapiro-Wilk W statistic of >0.98 (Table in Figure R3), indicating that they approximate a normal distribution.

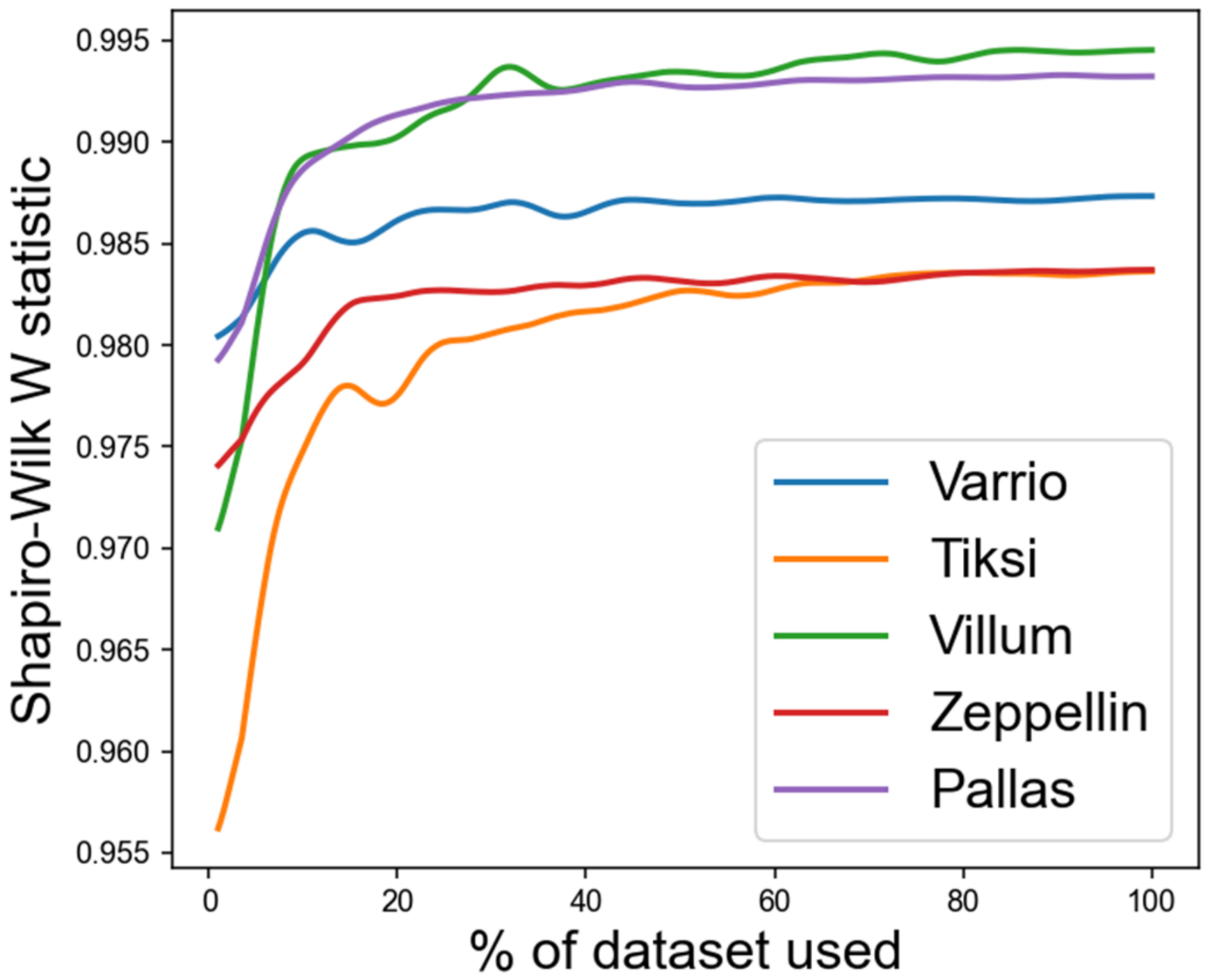

	Värriö	Tiksi	Villum	Zeppelin	Pallas
Shapiro-Wilk W statistic	0.99	0.98	0.99	0.98	0.99
n	7744	1484	2499	4719	3154

Figure R3. Dependence of the Shapiro–Wilk W statistic on the fraction of data used in bootstrap samples for different stations. Smoothed curves show how the W statistic changes with increasing sample size. A W=1 statistic would imply the sample data matches a perfect normal distribution. Inset table show the Shapiro-Wilk W Statistic applied to the full dataset at various stations and the total number of data points.

The last step - linking Potential_NPF to satellite-derived UV, H2O, and extinction using a regression - also seems a bit weak. For the study region, passive satellite retrievals (CERES in this case) are generally unreliable, which could bias the results. On top of that, the regression performance is not very strong (R2~0.5), and the errors are quite large (MAPE up to ~64%).

We agree with this criticism and have substantially upgraded the satellite regression in the revision. The original log-log multiple linear regression ($R^2 \approx 0.52$) derived from just four stations has been replaced with an XGBoost machine-learning algorithm trained on an expanded dataset incorporating six physically motivated predictors: UV radiation (UV), air temperature (T), water vapour content ($[H_2O]$), atmospheric aerosol load (σ_{ext}), terrestrial precursor vapours ([NDVI]) using NDVI (Normalised Difference Vegetation Index) as the proxy, and marine precursor vapours ([sea ice]) with sea ice concentration used as a proxy. Training used five-fold cross-validation on data from six locations: Zeppelin, Villum, Tiksi, Värriö, Utqiaġvik, and the Central Arctic campaigns. Pallas was withheld entirely as an independent test set.

The revised model achieved: Training $R^2 = 0.84$ (MAE = 0.18); Independent validation set (Pallas) $R^2 = 0.67$ (MAE = 0.32). Compared to the earlier model, the expanded parameters could capture more of the environmental drivers of NPF activity. The training dataset has also increased to include Värriö, Utqiagvik and the Central Arctic campaign data, thus expanding the representative of our estimations to other regimes such as the Maritime Arctic region. This revised model allowed us to test the predictions on an unseen location and so the error metrics would be more robust. Precisely, the revised model could replicate 56% of the variability in $Potential_{NPF}$, which is reasonable given that the parameterisation was performed with observational datasets.

Regarding CERES unreliability and bias, other studies have shown that CERES has good performance over our study area. Comparison of CERES shortwave radiation retrieval over the Arctic to in-situ observations were found to have a $R^2 = 0.92$, outperforming other satellite-based products such as ISCCP-FD and GEWEX-SRB (Sun et al., 2018). Another study found that CERES's estimates in the Arctic performed be the best out of 11 products with accuracy of $R^2 = 0.86$ (Wang et al., 2021). Another comparison of CERES's products found that accuracy decreases with decreasing latitude with the polar areas having the highest accuracy of R^2 ~0.9 (Zhang et al., 2022). Therefore, we believe that the CERES retrievals use in this study is valid.

For the revised manuscript, the "Satellite-derived $Potential_{NPF}$ maps" section in the Methods have been rewritten to explain the new approach. Since the output $Potential_{NPF}$ has also been edited, the results and discussion sections were rewritten to the updated values. In the Supporting Information, SI Text S2 has been rewritten for the new $Potential_{NPF}$ estimation approach.

The authors relate Potential_NPF based on UV, H2O, and extinction in the regression model, but only consider temperature in the future projections. Since H2O and extinction will also change, the resulting projections seem overly simplified. If the authors think T is important, why not include it in the regression model? The logic feels a bit self-contradictory.

This was a valid concern with the original manuscript that has been resolved in the revision in two ways.

First, temperature is now included as a predictor in the revised XGBoost regression (Eq. 4). See comment above.

Second, we have reframed our 'projections' as 'temperature-sensitivity estimates' throughout the revised manuscript. Of the six predictors in the revised regression, temperature was selected as the single sensitivity variable for future estimates for two reasons. Empirically, temperature is correlated with the other predictors in our dataset (SI Figure S5), consistent with its role as an integrative driver of Arctic environmental conditions. Practically, surface air temperature is among the most reliably projected CMIP6 variables in the Arctic, whereas future changes in the other variables carry substantially greater model uncertainty. The temperature-sensitivity estimates should therefore be understood as quantifying the NPF response to warming alone, with changes in the remaining predictors representing additional sources of uncertainty not yet quantified. We acknowledge this explicitly in the revised SI Text S4.

> *"Since temperature change is a driving force or indicator in many Arctic processes, temperature change was regarded as the proxy for environmental changes in the Arctic (Box et al., 2019). Among the six predictors in the XGBoost model, surface air temperature is also the most reliably projected CMIP6 variable in the Arctic, whereas future projections of the other variables carry substantially greater inter-model uncertainty. Furthermore, temperature, consistent with its role as a co-varying driver of Arctic atmospheric and surface conditions, is found to be strongly correlated to other environmental indicators such as water vapour, sea ice and terrestrial vegetation (SI Figure S5), justifying temperature changes as a good indicator of Arctic warming… Therefore, the temperature-sensitivity estimates presented here should therefore be understood as quantifying the PotentialNPF response to warming alone, with changes in the remaining predictors representing additional sources of uncertainty not yet quantified."*

SI Figure S5:

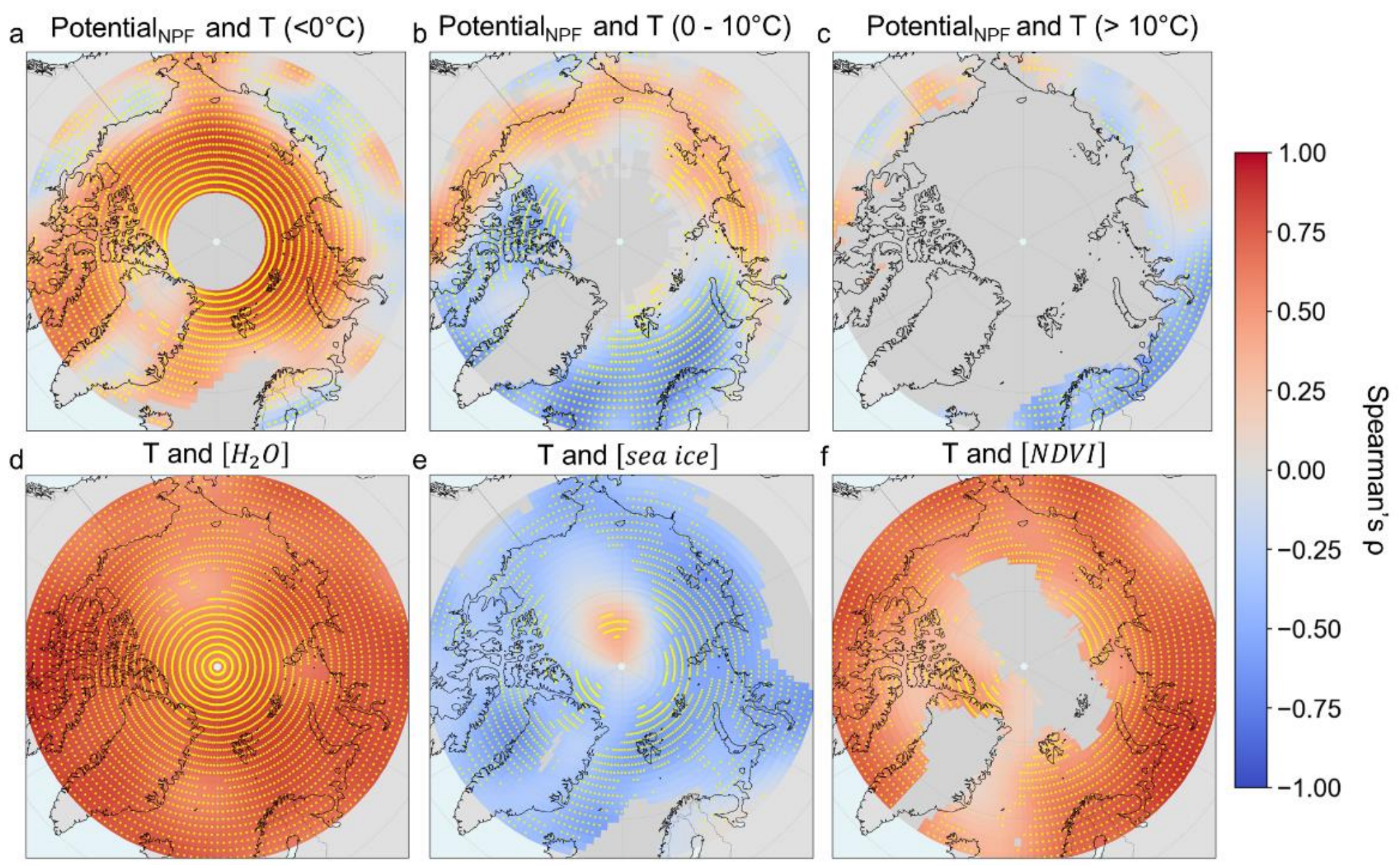

Figure S5. Spearman's ρ values between surface air temperature (T) and $Potential_{NPF}$ calculated at each pixel for data during 2006-2021 March-August at three temperature bins: (a) <0 °C, (b) 0-10 °C, and (c) >10 °C. For visualisation of the sensitivity of the satellite variables to T, Spearman's ρ values were calculated at each pixel for data during 2006-2021 March-August between surface air temperature (T) and (d) water vapour content ($[H_2O]$), (e) sea ice concentration ($[sea\ ice]$), and (f) Normalised Difference Vegetation Index ($[NDVI]$). The values of $[NDVI]$ and $[sea\ ice]$ at each pixel were computed as the spatial mean across a 500 km buffer. Pixels where ρ value is statistically significant ($p < 0.1$) are marked by yellow dots.

With all this, the derivation of Potential_ NPF doesn't feel fully convincing for subsquent consclusions...

In response to the comments raised above, we have carefully re-evaluated and revised the $Potential_{NPF}$ estimation framework accordingly. We believe that these revisions have strengthened the robustness of the methodology and adequately addressed the reviewer's concerns.

Abstract: The phrase "a combined total of 60 years of data" is somewhat misleading. Only after reading the Methods in more detail did I realise that this refers to the total volume of data across all sites, rather than the actual time period covered. The dataset is actually from 2010-2019, is that right? Please clarify this in the main text and avoid using the phrase "60 years of data", as it may be very misleading.

We agree the original phrasing was misleading. The "60 combined years" refers to the sum of valid observation-days across all stations (21,705 days in total, spanning data periods from 2003 to 2024), not a 60-year continuous record at any one site. The 2010–2019 period refers to the common 'window' with adequate data across multiple sites. that we used exclusively for trend analyses (Table S2).

We have clarified this throughout the manuscript and added explicit temporal coverage per site in SI Table S4, which now lists the data period and number of valid data days for each station. Various

edits were also made in the text to highlight the 2010-2019 ‘window’ or the varying data periods. The confusing “60-year” phrasing has also been removed throughout the manuscript.

In the main manuscript:

> *“During a common 2010-2019 window, significant declines in accumulation-mode…”*

> *“From the common 2010-2019 window, Arctic-wide declines in CS…”*

In the methods:

> *“The final harmonized dataset comprised of a total of 21705 valid data days across eight different Arctic locations spanning 2003-2024, although data coverage periods varied (SI Table S4).”*

In SI Text 1:

> *“…All in all, the final harmonized dataset comprised of a total of 21705 valid data days, although data coverage periods varied (SI Table S4)”*

Caption for Table S1:

> *“…The duration of the datasets differed across stations and campaigns: Zeppelin (08/2010–10/2024), Villum (07/2010–09/2020), Tiksi (07/2010–10/2018), Pallas (01/2012–06/2021), Värriö (01/2003–06/2024), Alert (03/2011–12/2018), Utqiaġvik (10/2019–12/2024), Central Arctic (08/2018–06/2023).”*

Fig. 1 : The text in Fig. 1 is quite small and hard to read. In Fig. 1a, the range (12.5–15) also doesn’t match what’s written in the caption (12.5–25), so that might need checking. It would also help to mark the sites for different regimes in different colours, so it’s easier to link the figure to the text.

Thank you for the suggestions. Figure 1 now look:

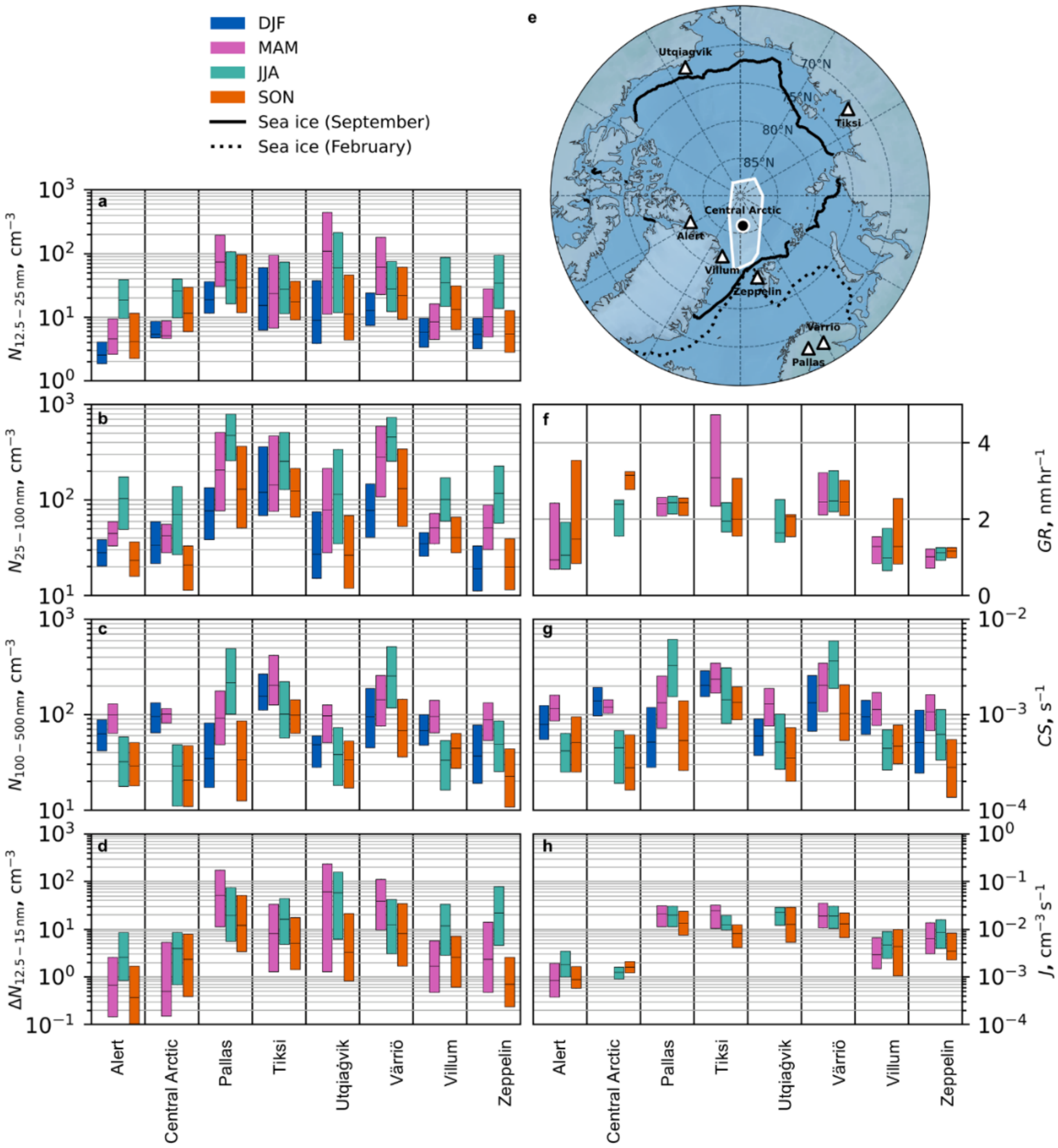

Figure 1. Aerosol parameters across eight Arctic locations (Alert, Central Arctic, Pallas, Tiksi, Utqiaġvik. Värrio, Villum, and Zeppelin) separated by traditional seasonal classification (December-February, March-May, June-August, and September-November) (a) Nucleation mode particles concentration: $N_{12.5\text{-}25\ nm}$; (b) Aitken mode particles concentration: $N_{25\text{-}100\ nm}$; (c) accumulation mode particles concentration: $N_{100\text{-}500\ nm}$; (d) diurnal variation in 12.5-15 nm particle concentration: $\Delta N_{12.5\text{-}15\ nm}$; (f) growth rate at 15-20 nm: GR; (g) condensation sink: CS; and (h) formation rate at 15 nm: J. Boxplots indicate the median and IQR of the parameters. (e) Map of various Arctic sites, a 'Central Arctic' polygon that shows the extent of the cruise campaigns, and maximum (February) and minimum (September) sea ice extent from 1981-2010 medians (Meier et al., 2024) .

Table S2 : Time evolution plots alongside Table S2 (perhaps in the SI) would be helpful to better visualize the trends from 2010 to 2019, compared to just listing numbers.

We agree with the reviewer that time-series plots can be helpful for visualising temporal trends, and we appreciate this suggestion. To keep the number of plots reasonable, we plotted the variables

related to NPF (CS, GR, J and $\Delta N_{12.5\text{-}15\ nm}$) during the NPF-active months of March-May and June-July in Figure S1.

Figure S1 looks:

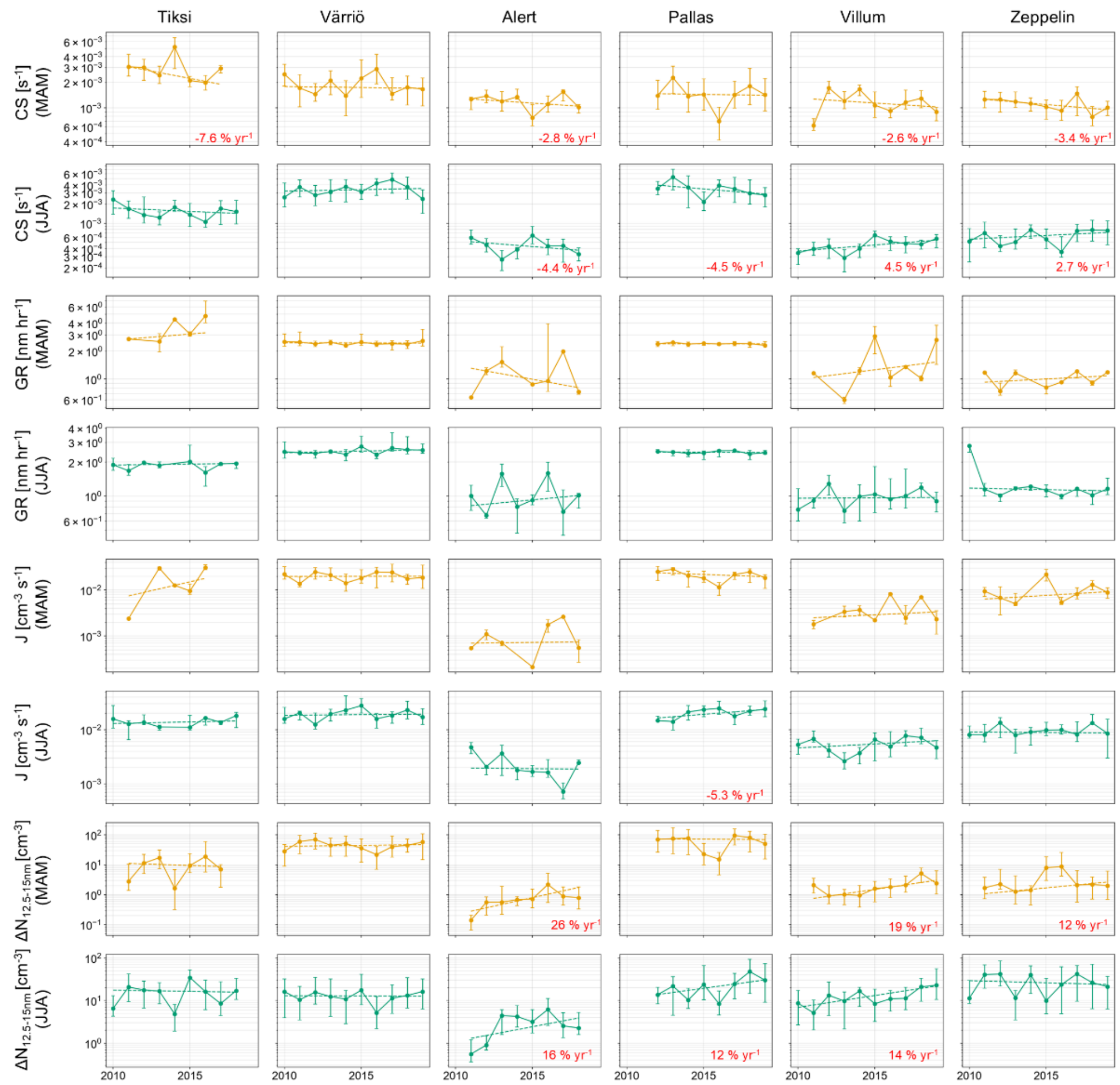

Figure S1. Timeseries of selected aerosol parameters from the common window of 2010-2019 separated by seasons (MAM: March- May, JJA: June-August) with the interquartile range at each yearly value shown. Trends were calculated from fitted Theil-Sen trend lines. Slopes that were statistically significant (Mann-Kendall test, $p<0.1$) were expressed as percentage change/year according to Eq. 6 and printed in red in the subplots. Parameters are CS: condensation sink; GR: growth rate at 15-20 nm; J: formation rate at 15 nm; and $\Delta N_{12.5\text{-}15\ nm}$: diurnal variation in 12.5-15 nm particle concentration.

L315: mean (μ) at 0.5 -> 1.5(?)

Thank you for pointing out the typo. Corrected.

L354–356: The description of the soft clustering is quite vague. Although not explicitly stated, I assume the intention is to match each satellite pixel to the parameterizations derived from the four sites. More details on the clustering method and weighting should be included in the Methods, not only in the SI, or at least clearly referenced there.

As we have changed the $Potential_{NPF}$ method to the XGBoost approach, the spatial clustering approach used previously was not necessary anymore. Section has been removed.

Reviewer #3

The manuscript discusses the development of cloud condensation nuclei (CCN) in the Arctic using observational data from different stations, campaigns, and satellite data. Combining these different data sets in a harmonised way enables the authors to investigate the relationship of new particle formation (NPF) and CCN concentration in Arctic regions. For future projections, the authors use SSP 5-8.5 models to forecast the change of NPF and thus CCNs in a warmer climate. The authors develop a new measure for NPF, Potential_NPF, which they use to compare the capability for NPF events in satellite and model data. For the analysis, they distinguish three different Arctic regions, the High Arctic, the Maritime Arctic, and Continental Eurasia. They find that the development of NPF varies between the different regions. While it increases in the High Arctic, the signal is not as unambiguous in the other regions.

The manuscript is well written and the argumentation is comprehensible. Both observational and model data are used for the study. The methods are well explained in the method section and supplementary information (SI). Combining the different data sets gives an interesting and new perspective and allows for Arctic wide comparison of NPF and CCN concentration which leads to the conclusion that it is not trivial to forecast the development of CCN concentration in a warmer climate since it strongly depends on the specific region in the Arctic and the different processes taken into account.

I consider the manuscript suitable for being published in Nature Geoscience after the following points have been addressed:

Thank you for the positive assessment of our manuscript, and for recognising the value of combining observational, satellite, and model datasets to investigate Arctic NPF and CCN variability. We have edited the manuscript and SI following the comments and suggestions below.

Fig. 1: Please comment on the physical process of condensation sinks also in the main text and not only in the appendix.

Edited. Section now reads:

> *"On the other hand, the potential increase in CCN number concentrations may be overstated, as higher aerosol concentrations from either local or transported sources would also enhance the condensation sink (CS). An increased CS, defined as the rate at which vapour molecules are removed from the atmosphere by condensation onto existing aerosol particles, would limit the formation and subsequent growth of newly formed particles to CCN-relevant sizes (Breider et al., 2010; Browse et al., 2014)."*

Line 69 ff: A brief description on the methodology for the harmonisation is missing here. It is described in the methods section and in the SI in more detail. However, I think it is important to have a short description here as well to interpret the results presented in the following. At least, there should be a reference to the explanation.

The harmonisation methods refer to our efforts to make the datasets comparable across stations and over longer durations (See also Reviewer #1 Comment 6 response). We have thus added a brief explanation on what harmonisation entails. An explicit reference is also added to point readers to SI Text S1 where the harmonisation process is explained in detail.

Methods now reads:

> *"The aerosol number size distributions were constrained to 12-500 nm to ensure comparability across observations. Site specific data inspection and quality-control procedures had been performed with more details on the specific procedures implemented at the individual observations documented in the corresponding references (Asmi et al., 2011, 2016; I. Beck et al., 2022; Boyer et al., 2023; Croft et al., 2016; Freud et al., 2017; Karlsson et al., 2022; Kyrö et al., 2014; Nguyen et al., 2016; Tunved et al., 2013; Väänänen et al., 2013)...*
>
> *…NPF relevant parameters, as part of the data harmonisation process, were computed using algorithmic objective methods(Aliaga et al., 2023; Kulmala et al., 2022; Lampilahti et al., 2025) to ensure inter-site comparability."*

SI Text 1 reads:

> *"…Although some sites have particle size number distribution (PNSD) extending down to 3 nm-sized particles, the lower cutoff for our harmonized dataset was chosen to be approximately 12 nm, as that is the limit where there is quality-controlled data available across all measurement sites. For Villum, data during 2019-2020 were limited to <360 nm due to instrument limitations. Specific anomaly-detection and calibration procedures applied at each site were documented in the corresponding station references listed in SI Table S4…*
>
> *…Compared to traditional methods that could be subjective and/or time-consuming (Bhandari et al., 2025; Dal Maso et al., 2005), procedures to objectively and quickly quantify NPF events was applied in this study to ensure inter-site comparability."*

Line 69 ff: A statement about temporal data coverage of the different stations is missing.

The line is edited to:

> *"Altogether, an unprecedented combined dataset spanning 2003–2024 across eight different Arctic locations, albeit with varying data coverage periods between sites, was compiled to provide new insight into the spatiotemporal variability of aerosol populations and NPF activity across the Arctic."*

In the Methods:

> *"The final harmonized dataset comprised of a total of 21705 valid data days across eight different Arctic locations spanning 2003-2024, although data coverage periods varied (SI Table S4)."*

Please add a comment about the used instruments used to collect the data at the different stations. The table in the SI is not very well connected to the text and can be easily missed.

Additional information are provided in the methods:

> *"Measurements were collected using Differential Mobility Particle Sizers (DMPS) or Scanning Mobility Particle Sizers (SMPS) to obtain size-differentiated aerosol particle*

> *number concentrations. Further details of the instrument models used at each site are provided in SI Table S4."*

Fig. 1: I am not completely sure how you calculate ΔN. Can you please explain this bit more comprehensively?

Line in "Latitudinal and seasonal gradients" section in the main manuscript now reads:

> *"As a proxy for NPF intensity,* $\Delta N_{12.5-15}$*, defined as the difference between the daily peak particle number concentration in the 12.5–15 nm size bin from the daily background hours…"*

In the Methods:

> *"In greater detail,* $\Delta N_{12.5-15}$ *is the difference between the daily peak particle number concentration in the 12.5–15 nm size bin and the median* $N_{12.5-15}$ *during the background hours (the four consecutive hours with the lowest median* $N_{12.5-15}$ *in each month), thus capturing the amplitude of the diurnal NPF signal while minimising contamination from background variability."*

Line 146 ff and Fig. 2: Can you compare the values of Potential_NPF to other regions than the Arctic to get a better feeling for the numbers? For example, is 50 a high or a low value? Since it is a newly defined parameter, it is hard to get a feeling about the meaning of the specific values.

This is a very helpful suggestion. However, following our updated $\mathrm{Potential_{NPF}}$ model, we need more tests and calibration to ensure that our $\mathrm{Potential_{NPF}}$ estimation process are still valid and interpretable outside of the Arctic region. Instead, we have related $\mathrm{Potential_{NPF}}$ to other metrics such as formation rates or practically, the number of days with NPF events. We found that formation rates of 15nm particles ($\mathrm{J_{15}}$) increases with increasing $\mathrm{Potential_{NPF}}$. For instance, when $\mathrm{Potential_{NPF}}$ = 20, $\mathrm{J_{15}}$ ~0.1 $cm^{-3}s^{-1}$. We also manually classified the Värriö dataset using the traditional Dal Maso (2005) method. We found that the monthly values of $\mathrm{Potential_{NPF}}$ approximately equals to the percentage of days within that month which Class I/II events are noted to occur (R^2=0.72). Thus, $\mathrm{Potential_{NPF}}$ can be considered as the probability of days where NPF events are likely to occur. We have edited SI Text S2 to update the evaluation approaches.

The section in SI Text S1 reads:

> *"Importantly,* $Potential_{NPF}$ *should be interpreted as a probabilistic metric that reflects the position of the observed* n_z *value within the NPF-mode distribution. Despite this probabilistic formulation,* $Potential_{NPF}$*, when compared to formation rates of 15 nm-particles (*J_{15}*), showed that* $Potential_{NPF}$ *increased with increasing* J_{15} *(SI Figure S7b), justifying* $Potential_{NPF}$ *as an indicator for NPF intensity. At Värriö, the longest dataset in our records, monthly values of* $Potential_{NPF}$ *also correlated well in a 1:1 relationship with the monthly percentage of days with Class I and Class II events (classified according to Dal Maso et al. (2005)) (SI Figure S7c). Therefore, an approximation could be made, with care, that the monthly value of* $Potential_{NPF}$ *roughly corresponds to the percentage of days within that month during which NPF events are likely to occur."*

SI Figure S7 looks:

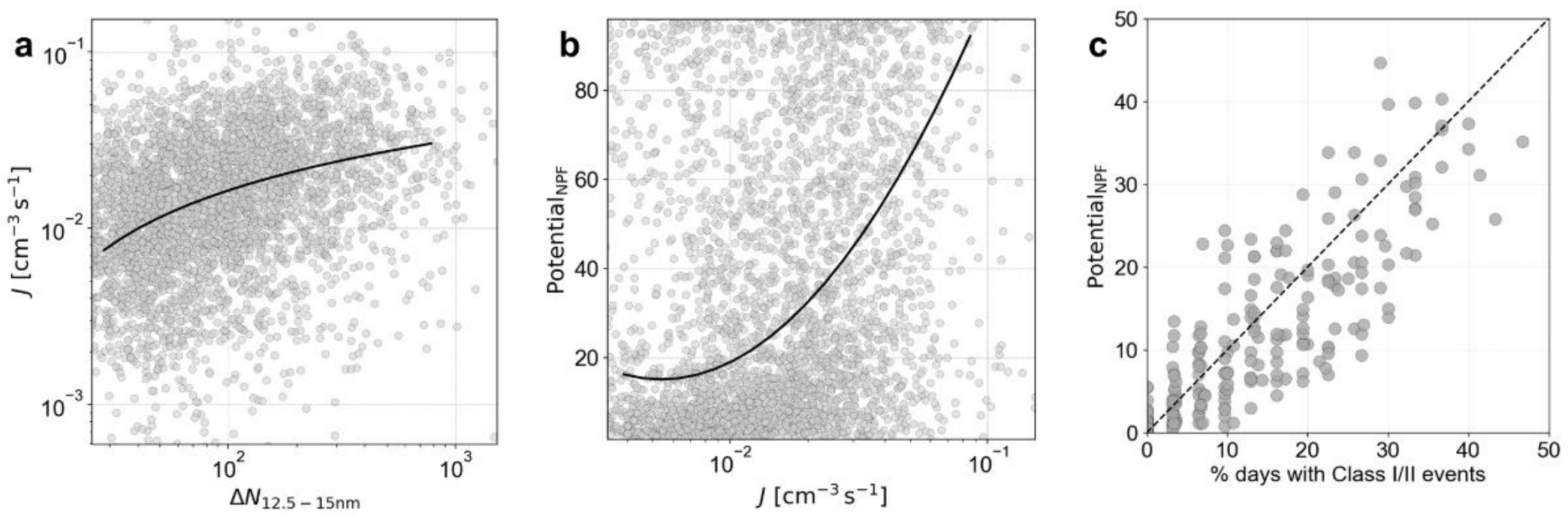

Figure S7. (a) Formation rates of 15 nm particles (J_{15}), against $\Delta N_{12.5-15}$ values, and (b) $Potential_{NPF}$ against formation rates of 15 nm particles (J_{15}) across all stations. The black lines indicate the median while the grey scatter indicates raw datapoints. (c) Monthly values of $Potential_{NPF}$ at Värriö against the corresponding monthly value of the percentage of days with Class I/II events (according to Dal Maso et al. (2005)) observed. Dashed line is the 1:1 ratio.

Fig. 3 and Line 289 ff: I understand that you use the station data to compare them to the satellite derived Potential_NPF. However, in the Maritime Arctic there is no station. Do you use campaign data for this, e.g. MOSAiC? If so (and also in general for the used ship-borne data), please add a comment about the different temporal and spatial coverages compared to the stations and how this biases the results.

In this revised $\mathrm{Potential_{NPF}}$ estimation with XGBoost, we do use the cruise datasets as input training data. Indeed, the dearth of ocean-measurements make constraining our understanding of NPF-CCN interactions very difficult. The combined cruise-based observations (Arctic Ocean 2018, MOSAiC, ARTofMELT), despite the extensive efforts required to collect the observational datasets, are still insufficient for establishing climatological baseline comparable to that from land-based sites. We have added in statements to express this limitation and the need for greater observational focus on open-water regions of the Arctic.

In the "NPF potential and CCN under a warming Arctic" section in the main manuscript

> *"Unlike the other regimes, the Maritime Arctic lacks permanent monitoring stations; thus, observational constraints here derive primarily from short-duration research cruises with limited temporal and spatial coverage, challenging our ability to accurately constraint $Potential_{NPF}$ or N_{CCN} estimates."*

In SI Text S2:

> *"However, a limitation of the machine learning model is that the cruise-based observations (Arctic Ocean 2018, MOSAiC, ARTofMELT) are much shorter in duration than the long-term terrestrial stations so the estimated $Potential_{NPF}$ values may not be representative of Maritime Arctic conditions year-round. In particular, the MOSAiC expedition occurred during an extreme positive Arctic Oscillation year (2019–2020), which coincided with anomalously high wintertime accumulation-mode concentrations (Boyer et al., 2023). Therefore the $Potential_{NPF}$ values over open ocean would benefit from dedicated long-term measurements to better constrain them."*

Line 185 ff: Relate this statement more concretely to the figure.

This sentence, as part of the section on Continental Eurasia aerosols, have now been removed following the revised classification

Fig. 4: I like the figure. What do the grey circles stand for? Add an explanation to the caption.

Figure 4 has been revised following the updated datasets. The legend of the grey circles are labelled within the figure.

Figure 4 now looks:

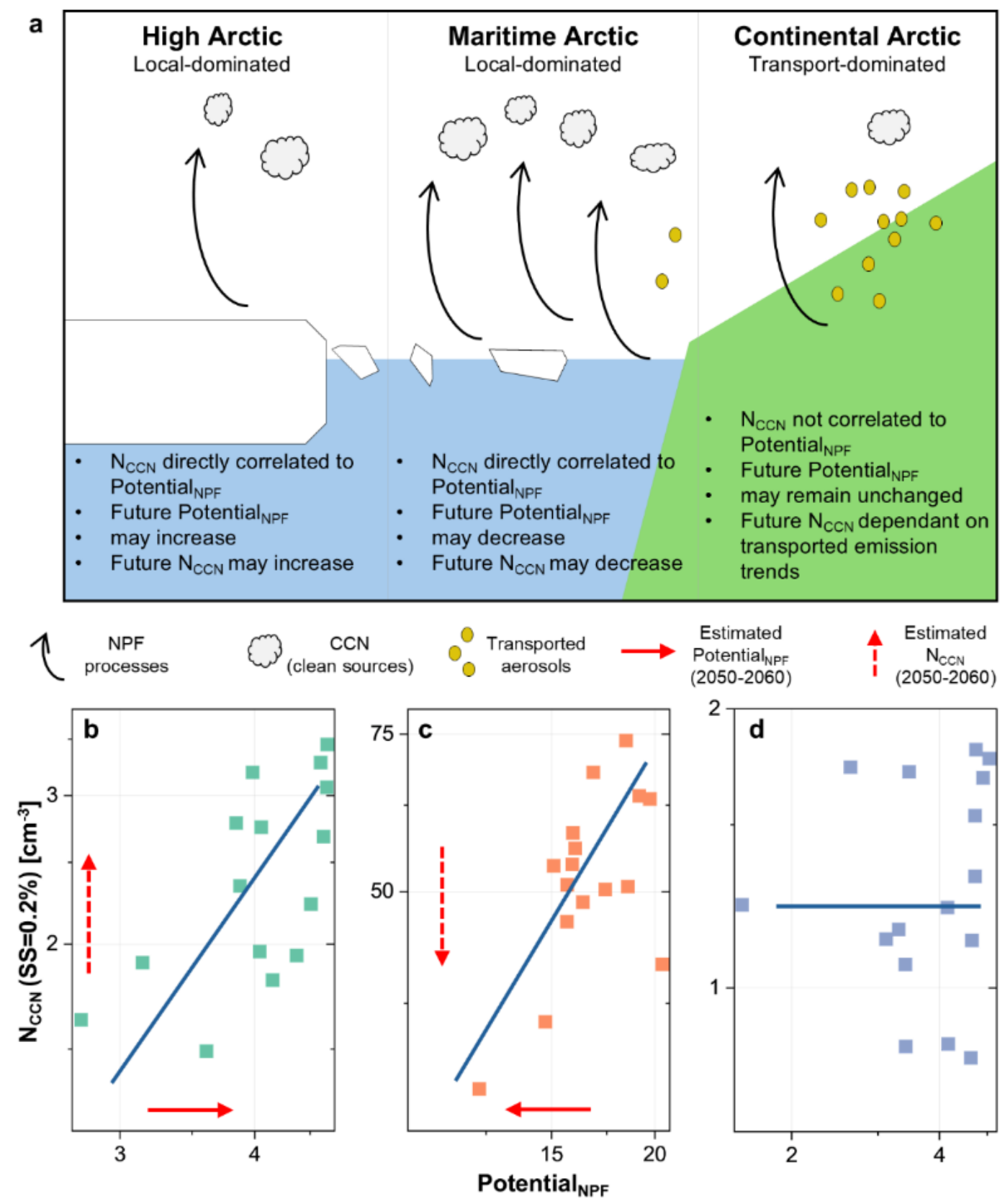

Figure 4. (a) Conceptual figure of the NPF-CCN relationships in the three Arctic regimes: High Arctic, Maritime Arctic, and Continental Arctic. Scatterplots of N_{CCN} against $Potential_{NPF}$ in the b) High Arctic, c) Maritime Arctic, d) Continental Arctic. Each point represents a yearly area-weighted median of March-August N_{CCN} and $Potential_{NPF}$ values. To guide the eye towards the trend of the weighted scatters, blue lines were drawn in (b), (c), and (d). Red solid arrows indicate the estimated temperature-sensitivity trend of $Potential_{NPF}$ by 2050-2060 following the temperature projections under the SSP5-8.5 scenario. Red dashed arrows indicate the speculated temperature-sensitivity trend of N_{CCN} by 2050-2060 under the SSP5-8.5 scenario.

Line 207 ff: How good are the models at all in simulating NPF and CCNs, especially in polar regions? There should be a comment about the expected ability of the models to capture the studied processes, since I think, it can be questioned that they do a very good job here. How do the models compare to

observations looking at simulations without extra warming? Also, the models do not capture Arctic amplification correctly. So how can they be trusted here?

Using the earth system models to directly simulate NPF and CCNs are still works in progress, with parameterisation of aerosols and atmospheric chemistry still poorly represented. One of the future objectives of this study is to use satellite-based approach as one of the means to empirically constrain the parameterisation in physics-based models, which we are in the process of validating.

We agree with the reviewer here and in this revised manuscript, we have reworded the claims of "projection" to "temperature-sensitivity estimates" instead, because without knowledge of other processes such as future anthropogenic aerosol trends, an accurate projection is not possible. We also made a conscious decision to only use temperature as the sole parameterising variable as its uncertainty in the CMIP6 models are better constrained, as compared to other variables such as future VOC concentrations.

Historical CMIP6 temperature records deviated more strongly from observational values than the commonly used ERA5 reanalysis dataset, with a cold bias (Cai et al., 2021). Since our $Potential_{NPF}$-T parameterisation was done with the ERA5 dataset, we assume that the equations would be more accurate. However, because the temperature-sensitivity analysis into 2050-2060 were performed with the CMIP6 dataset, it is likely that the projections would have a cold bias too. Correspondingly, the temperature-sensitivity analyses in this manuscript are likely lower estimates.

Regarding the inaccuracy of arctic amplification that were also mentioned in the recent IPCC report, the lower-than-expected values were caused by overestimated global trends, rather than underestimated Arctic trends (Chylek et al., 2023). Nonetheless, considering the underestimates in Arctic air temperature, the estimated $Potential_{NPF}$ and N_{CCN} would be underestimates too. We have added in comment on the projected temperature uncertainty in SI Text S4:

SI Text S4:

> *"Given the known cold biases in historical Arctic temperatures simulated by many CMIP6 models* (Cai et al., 2021), *we would expect a corresponding cold bias in the projected temperatures used in our analysis. Consequently, the projected warming by 2050–2060 may itself be underestimated. As our $Potential_{NPF}$ and N_{CCN} projections are based on empirically derived temperature sensitivities, any underestimation in projected warming would likely translate into lower-magnitude projected changes in both $Potential_{NPF}$ and N_{CCN}. In this sense, our temperature-sensitivity estimates should be interpreted as conservative estimates rather than upper-bound projections (SI Table S3) …*
>
> *… The first set of "variability" range represented the upper and lower quantiles of possible outcomes, taking into account the model ensemble variability of Arctic warming estimates, $Potential_{NPF}$ and the fitted temperature-$Potential_{NPF}$ parameterizations (SI Figure S10)."*

Line 390: Can you relate this to the main text? Where do you use the temperatures explicitly? If you use it for the computation/comparison of the Potential_NPF, please comment above in the main text.

In the updated methodology, ERA5 temperature was used directly to compute $Potential_{NPF}$, which we have now stated explicitly.

In "Satellite-derived $Potential_{NPF}$ maps" section under Methods

> *"…Air temperature data were obtained from monthly ERA5 records (Copernicus Climate Change Service, 2019)…"*

Can you include a plot of the temporal evolution of the Potential_NPF and NPF-CCN relationship during the measurement period of the stations? I think, it would be a good addition to the future model prediction. Can you already recognise the trend shown in the models? Also, how does the trend in the satellite data look like, e.g. relating to Fig. S1?

We have included Figure S1 which shows the NPF relevant parameters during the 2010-2019 common window. The timeseries would help to show the trends labelled in Table S2.

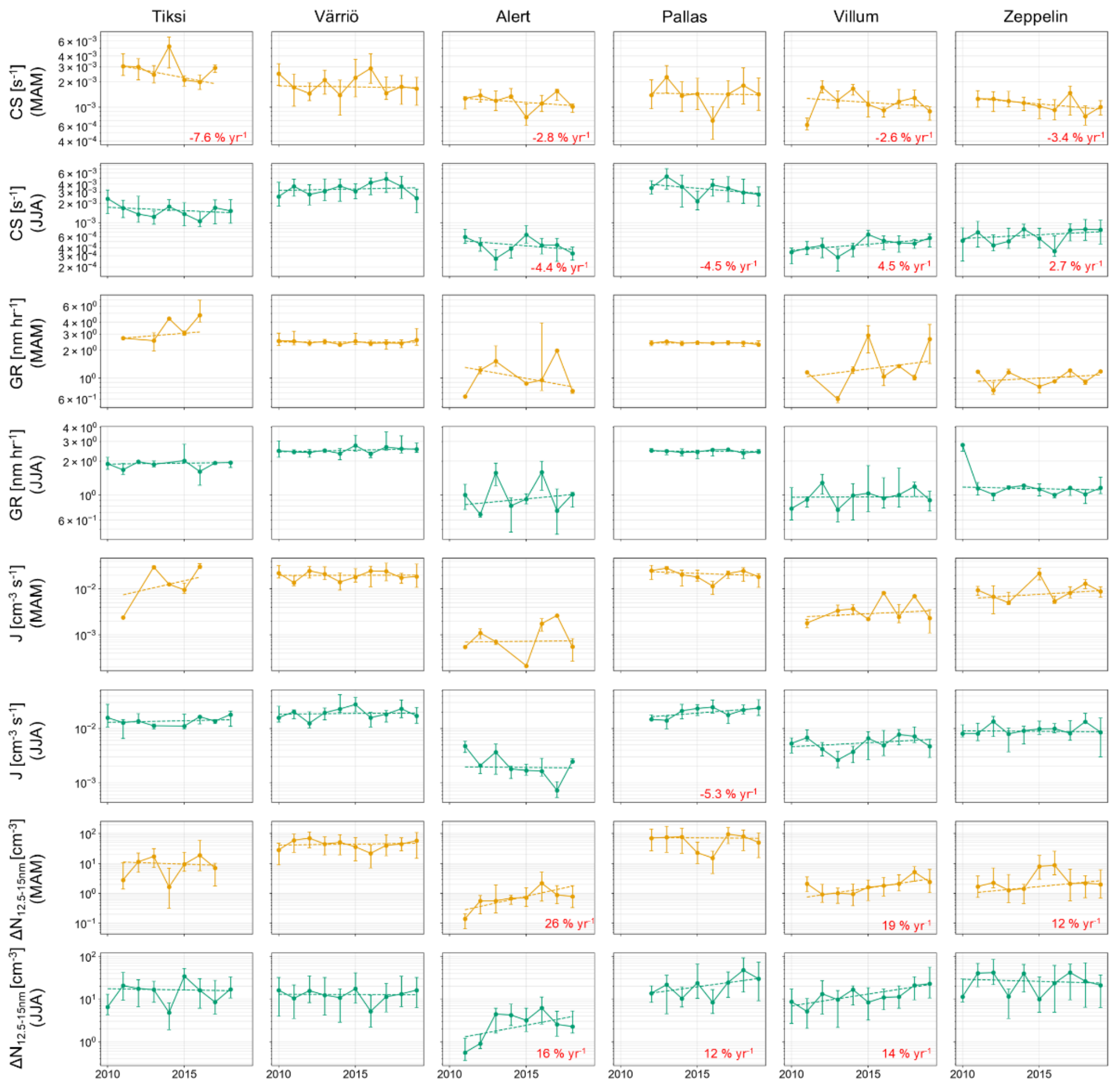

Figure S1. Timeseries of selected aerosol parameters from the common window of 2010-2019 separated by seasons (MAM: March- May, JJA: June-August) with the interquartile range at each yearly value shown. Trends were calculated from fitted Theil-Sen trend lines. Slopes that were statistically significant (Mann-Kendall test, $p<0.1$) were expressed as percentage change/year according to Eq. 6 and printed in red in the subplots. Parameters are CS: condensation sink; GR: growth rate at 15-20 nm; J: formation rate at 15 nm; and $\Delta N_{12.5\text{-}15\ nm}$: diurnal variation in 12.5-15 nm particle concentration.

In Figure R4 below, we plotted $Potential_{NPF}$ at each station and performed trend analysis there. The trends that we find here mirrors the trends of $\Delta N_{12.5\text{-}15\ nm}$: diurnal variation in 12.5-15 nm particle concentration as reported in SI Table S2. The similar result is expected as $Potential_{NPF}$ is derived mathematically from $\Delta N_{12.5\text{-}15\ nm}$ (Equation 1). For reference, only March-May $Potential_{NPF}$ at Villum were significantly increasing ($p<0.05$). However, we could not directly compare the stations values to the gridded satellite-derived $Potential_{NPF}$ because the revised $Potential_{NPF}$ estimation model uses the observational data as a training dataset, implying that it will likely replicate the observational values.

Thank you for the suggestion for plotting the satellite trends. Trends in the satellite-derived variables were computed for March–May and June–August over the 2006–2021 period. From Figure R5, we cannot identify a single satellite-derived variable that solely explains the observed trends in $Potential_{NPF}$ shown in Figure 2b,e, indicating that the trend in $Potential_{NPF}$ were indeed caused by multiple factors. Nevertheless, we do note several interesting trends that could be subject of future studies. We observed that σ_{ext} decreased during March–May, supporting our interpretation of a declining condensation sink during the Arctic Haze season (Table S2). In addition, sea-ice concentration decreased while NDVI increased during both March–May and June–August. However, the direct attributions of these individual changes to the observed $Potential_{NPF}$ trends would require an explicit physics-based model which is out of the scope of our current approach.

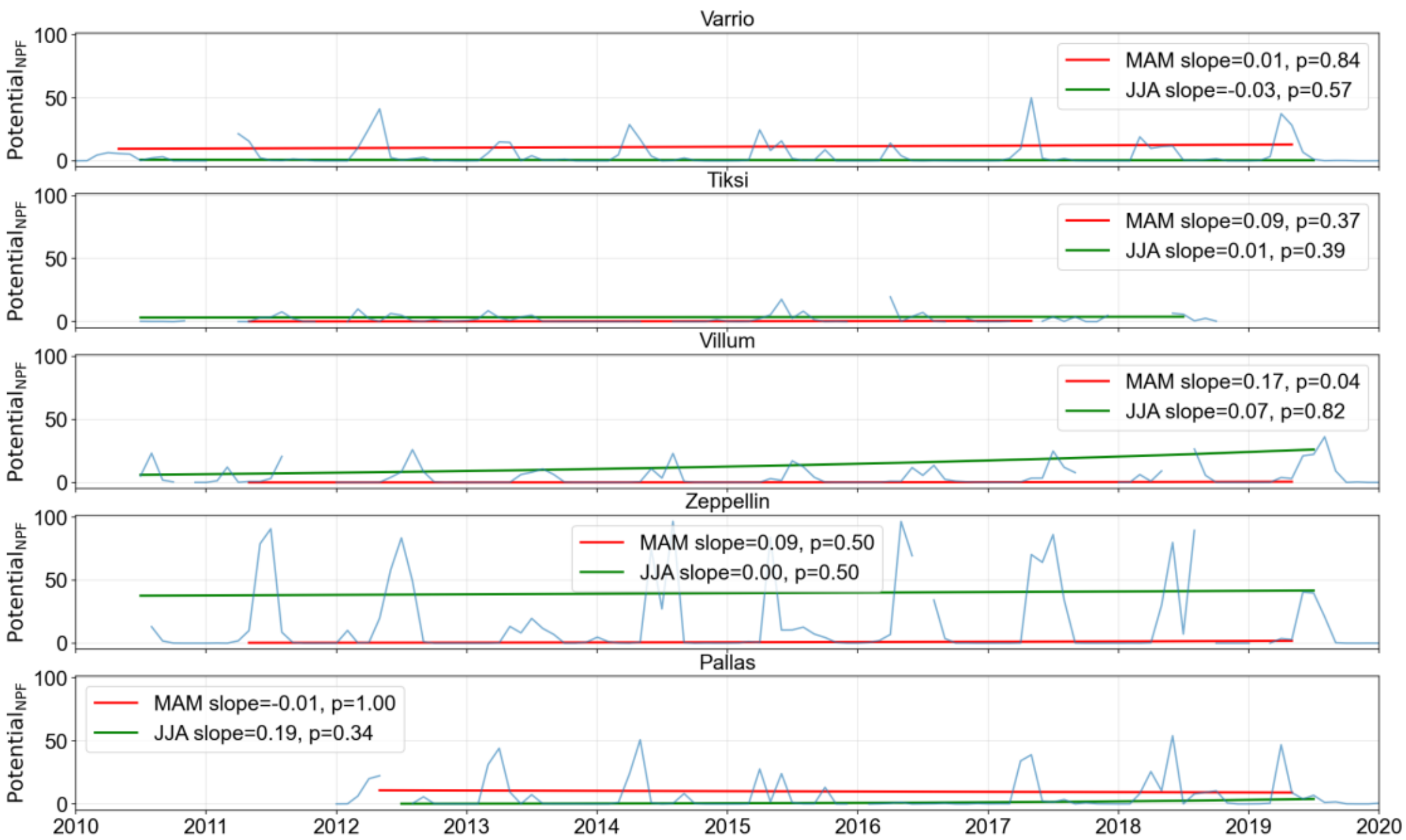

Figure R4: Timeseries plots of $Potential_{NPF}$ computed from observations at the respective stations. The red and green linear lines indicate the trend lines determined from Thiel-Sen trend analyses of the respective seasons: March-May and June-August. P-values were obtained from the corresponding Mann-Kendall seasonal test.

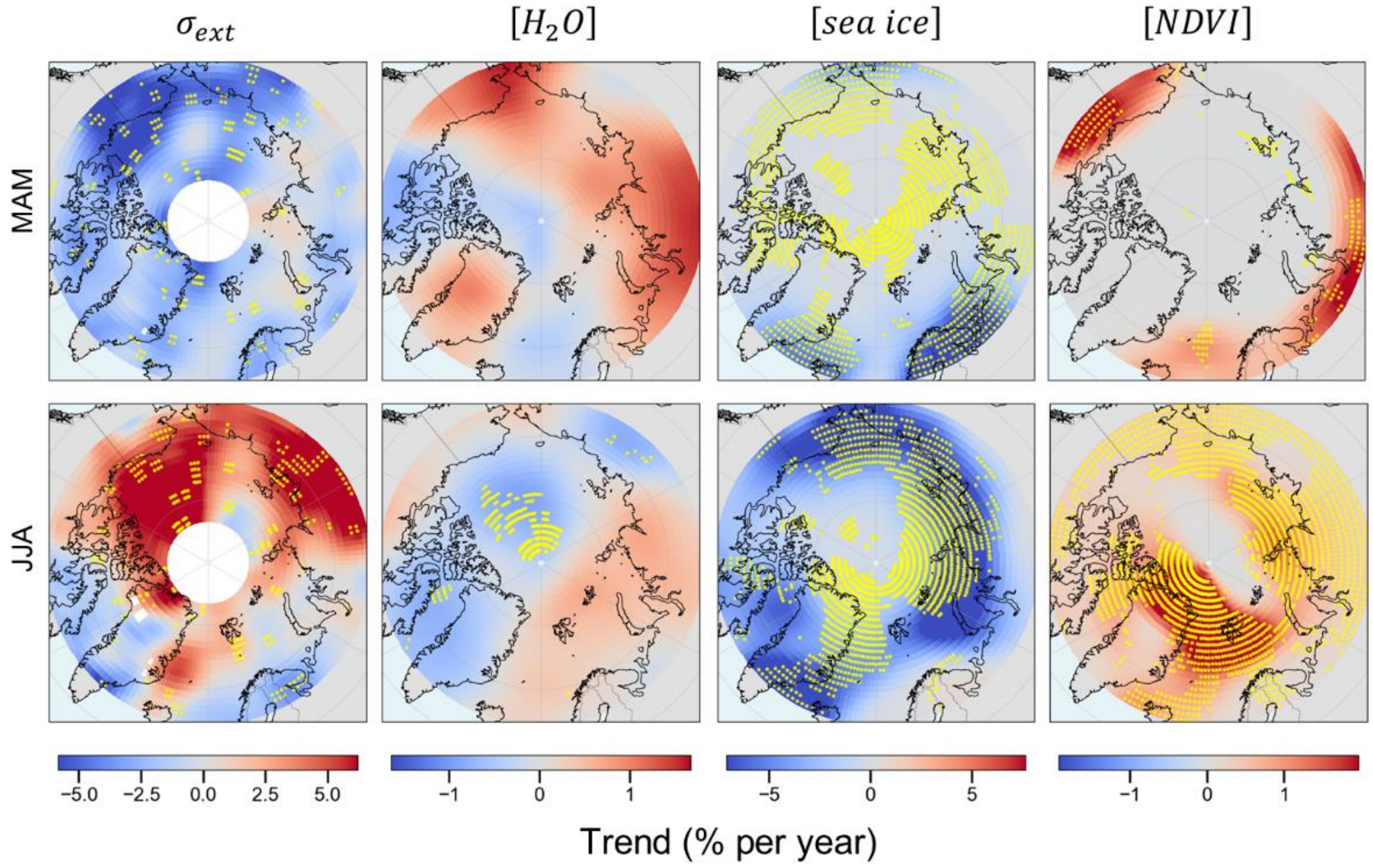

Figure R5: Yearly change in various satellite variables (atmospheric aerosol loading (σ_{ext}), water vapour content ($[H_2O]$), vegetation indices ($[NDVI]$), and sea-ice concentration ($[sea\ ice]$),) over 2006-2021 during months of March-May (top row) and June-August (bottom row) expressed as percentage change per year. Pixels where trend is statistically significant (Mann-Kendall seasonal test, $p < 0.1$) are marked by yellow dots.

SI Line 42 and Fig. S7: Is Z_score the same as n_z? Can you explain a bit more?

Yes, they are the same. The mentions of Z_{score} are now corrected to n_z for consistency throughout, also in Figure S6 and S8.

SI Line 42 is now:

> *"To enable inter-site comparison, Z-normalization was applied to $log\ \Delta N_{12.5-15}$ values following Eq. 1a, with the resulting Z_{score} denoted as n_z."*

SI Line 52: I am a bit confused about mu and sigma you use, also in the main text. Are you using the same mean and variance for all stations or is this the result of a fit? Did you calculate the numbers or did you get them from somewhere?

The $\mu = 1.5$ and $\sigma = 0.56$ values were mathematically derived from fitting five KDE curves to the general normal distribution of n_z aka the 'unseen station' case (see SI Figure S8). For the mean and variance of the respective stations as in the inset Table in Figure S8b, the mean and variance were fitted for each station to derive the normalized Mode 5 'banana plots' in Figure S6. The mean and variance from the observations ($\mu \approx 1.2–1.6$, $\sigma \approx 0.37–0.50$) show that our empirical estimate of the 'unseen case' is comparable.

Line 52: Typo. I guess you mean „at times"

Corrected.

Line 247: Check sentence. Do you mean „than local NPF potential“?

Sentence edited to "…likely depend more on anthropogenic emissions than on local NPF potentials."

Line 339/344: Introduce the abbreviations UV and [H20] when they are used for the first time in line 339.

Abbreviations were introduced, "… six proxy predictors were included, namely UV radiation (UV), air temperature (T), water vapour content ($[H_2O]$), …"

SI Line 108/109: Typo. Either "with increasing…“ or "as Potential increases“.

Line edited to "… showed increasing evolution of the characteristic NPF banana shape as $Potential_{NPF}$ increases…"

Line 208: I would suggest to add „The" before „Table" in the caption of S8.

As S8 has been reworked, the caption has changed to fit the new plot.

References (including those quoted from manuscript)

Aliaga, D., Tuovinen, S., Zhang, T., Lampilahti, J., Li, X., Ahonen, L., Kokkonen, T., Nieminen, T., Hakala, S., Paasonen, P., Bianchi, F., Worsnop, D., Kerminen, V.-M., & Kulmala, M. (2023). Nanoparticle ranking analysis: Determining new particle formation (NPF) event occurrence and intensity based on the concentration spectrum of formed (sub-5 nm) particles. Aerosol Research, 1(1), 81–92. https://doi.org/10.5194/ar-1-81-2023

Asmi, E., Kivekäs, N., Kerminen, V.-M., Komppula, M., Hyvärinen, A.-P., Hatakka, J., Viisanen, Y., & Lihavainen, H. (2011). Secondary new particle formation in Northern Finland Pallas site between the years 2000 and 2010. Atmospheric Chemistry and Physics, 11(24), 12959–12972. https://doi.org/10.5194/acp-11-12959-2011

Asmi, E., Kondratyev, V., Brus, D., Laurila, T., Lihavainen, H., Backman, J., Vakkari, V., Aurela, M., Hatakka, J., Viisanen, Y., Uttal, T., Ivakhov, V., & Makshtas, A. (2016). Aerosol size distribution seasonal characteristics measured in Tiksi, Russian Arctic. Atmospheric Chemistry and Physics, 16(3), 1271–1287. https://doi.org/10.5194/acp-16-1271-2016

Beck, I., Angot, H., Baccarini, A., Dada, L., Quéléver, L., Jokinen, T., Laurila, T., Lampimäki, M., Bukowiecki, N., Boyer, M., Gong, X., Gysel-Beer, M., Petäjä, T., Wang, J., & Schmale, J. (2022). Automated identification of local contamination in remote atmospheric composition time series. Atmospheric Measurement Techniques, 15(14), 4195–4224. https://doi.org/10.5194/amt-15-4195-2022

Beck, L. J., Sarnela, N., Junninen, H., Hoppe, C. J. M., Garmash, O., Bianchi, F., Riva, M., Rose, C., Peräkylä, O., Wimmer, D., Kausiala, O., Jokinen, T., Ahonen, L., Mikkilä, J., Hakala, J., He, X., Kontkanen, J., Wolf, K. K. E., Cappelletti, D., … Sipilä, M. (2021). Differing Mechanisms of New Particle Formation at Two Arctic Sites. Geophysical Research Letters, 48(4), e2020GL091334. https://doi.org/10.1029/2020GL091334

Bhandari, R., Sarangi, C., Sebastian, M., Hooda, R. K., Hyvärinen, A.-P., Asmi, E., Vakkari, V., Pandithurai, G., Singh, S., Soni, V. K., Nieminen, T., Van Zyl, P. G., Jaars, K., Laakso, L. K., Beddows, D. C. S., Harrison, R. M., Beukes, J. P., Kalivitis, N., Mihalopoulos, N., … Kanawade, V. P. (2025). Identification of New Particle Formation Events Using a You Only Look Once (YOLO) Deep Learning Algorithm. ACS ES&T Air, 2(7), 1202–1213. https://doi.org/10.1021/acsestair.5c00021

Bourgeois, Q., & Bey, I. (2011). Pollution transport efficiency toward the Arctic: Sensitivity to aerosol scavenging and source regions. Journal of Geophysical Research, 116(D8). https://doi.org/10.1029/2010jd015096

Box, J. E., Colgan, W. T., Christensen, T. R., Schmidt, N. M., Lund, M., Parmentier, F.-J. W., Brown, R., Bhatt, U. S., Euskirchen, E. S., Romanovsky, V. E., Walsh, J. E., Overland, J. E., Wang, M., Corell, R. W., Meier, W. N., Wouters, B., Mernild, S., Mård, J., Pawlak, J., & Olsen, M. S. (2019). Key indicators of Arctic climate change: 1971–2017. Environmental Research Letters, 14(4), 045010. https://doi.org/10.1088/1748-9326/aafc1b

Boyer, M., Aliaga, D., Pernov, J. B., Angot, H., Quéléver, L. L. J., Dada, L., Heutte, B., Dall'Osto, M., Beddows, D. C. S., Brasseur, Z., Beck, I., Bucci, S., Duetsch, M., Stohl, A., Laurila, T., Asmi, E., Massling, A., Thomas, D. C., Nøjgaard, J. K., … Jokinen, T. (2023). A full year of aerosol size distribution data from the central Arctic under an extreme positive Arctic Oscillation: Insights from the Multidisciplinary drifting Observatory for the Study of Arctic Climate (MOSAiC) expedition. Atmospheric Chemistry and Physics, 23(1), 389–415. https://doi.org/10.5194/acp-23-389-2023

Boyer, M., Aliaga, D., Quéléver, L. L. J., Bucci, S., Angot, H., Dada, L., Heutte, B., Beck, L., Duetsch, M., Stohl, A., Beck, I., Laurila, T., Sarnela, N., Thakur, R. C., Miljevic, B.,

Kulmala, M., Petäjä, T., Sipilä, M., Schmale, J., & Jokinen, T. (2024). The annual cycle and sources of relevant aerosol precursor vapors in the central Arctic during the MOSAiC expedition. Atmospheric Chemistry and Physics, 24(22), 12595–12621. https://doi.org/10.5194/acp-24-12595-2024

Breider, T. J., Chipperfield, M. P., Richards, N. A. D., Carslaw, K. S., Mann, G. W., & Spracklen, D. V. (2010). Impact of BrO on dimethylsulfide in the remote marine boundary layer. Geophysical Research Letters, 37(2), 2009GL040868. https://doi.org/10.1029/2009GL040868

Breider, T. J., Mickley, L. J., Jacob, D. J., Ge, C., Wang, J., Payer Sulprizio, M., Croft, B., Ridley, D. A., McConnell, J. R., Sharma, S., Husain, L., Dutkiewicz, V. A., Eleftheriadis, K., Skov, H., & Hopke, P. K. (2017). Multidecadal trends in aerosol radiative forcing over the Arctic: Contribution of changes in anthropogenic aerosol to Arctic warming since 1980. Journal of Geophysical Research: Atmospheres, 122(6), 3573–3594. https://doi.org/10.1002/2016jd025321

Browse, J., Carslaw, K. S., Mann, G. W., Birch, C. E., Arnold, S. R., & Leck, C. (2014). The complex response of Arctic aerosol to sea-ice retreat. Atmospheric Chemistry and Physics, 14(14), 7543–7557. https://doi.org/10.5194/acp-14-7543-2014

Cai, Z., You, Q., Wu, F., Chen, H. W., Chen, D., & Cohen, J. (2021). Arctic Warming Revealed by Multiple CMIP6 Models: Evaluation of Historical Simulations and Quantification of Future Projection Uncertainties. Journal of Climate, 34(12), 4871–4892. https://doi.org/10.1175/JCLI-D-20-0791.1

Chang, R. Y.-W., Sjostedt, S. J., Pierce, J. R., Papakyriakou, T. N., Scarratt, M. G., Michaud, S., Levasseur, M., Leaitch, W. R., & Abbatt, J. P. D. (2011). Relating atmospheric and oceanic DMS levels to particle nucleation events in the Canadian Arctic. Journal of Geophysical Research, 116, D00S03. https://doi.org/10.1029/2011JD015926

Choudhury, G., & Tesche, M. (2023). A first global height-resolved cloud condensation nuclei data set derived from spaceborne lidar measurements. Earth System Science Data, 15(8), 3747–3760. https://doi.org/10.5194/essd-15-3747-2023

Chylek, P., Folland, C. K., Klett, J. D., Wang, M., Lesins, G., & Dubey, M. K. (2023). High Values of the Arctic Amplification in the Early Decades of the 21st Century: Causes of Discrepancy by CMIP6 Models Between Observation and Simulation. Journal of Geophysical Research: Atmospheres, 128(23), e2023JD039269. https://doi.org/10.1029/2023JD039269

Copernicus Climate Change Service. (2019). ERA5 monthly averaged data on single levels from 1940 to present [Dataset]. Copernicus Climate Change Service (C3S) Climate Data Store (CDS). https://doi.org/10.24381/CDS.F17050D7

Croft, B., Martin, R. V., Leaitch, W. R., Tunved, P., Breider, T. J., D'Andrea, S. D., & Pierce, J. R. (2016). Processes controlling the annual cycle of Arctic aerosol number and size distributions. Atmospheric Chemistry and Physics, 16(6), 3665–3682. https://doi.org/10.5194/acp-16-3665-2016

Dada, L., Ylivinkka, I., Baalbaki, R., Li, C., Guo, Y., Yan, C., Yao, L., Sarnela, N., Jokinen, T., Daellenbach, K. R., Yin, R., Deng, C., Chu, B., Nieminen, T., Wang, Y., Lin, Z., Thakur, R. C., Kontkanen, J., Stolzenburg, D., … Kulmala, M. (2020). Sources and sinks driving sulfuric acid concentrations in contrasting environments: Implications on proxy calculations. Atmospheric Chemistry and Physics, 20(20), 11747–11766. https://doi.org/10.5194/acp-20-11747-2020

Dal Maso, M., Kulmala, M., Riipinen, I., Wagner, R., Hussein, T., Aalto, P. P., & Lehtinen, K. E. (2005). Formation and growth of fresh atmospheric aerosols: Eight years of aerosol size

distribution data from SMEAR II, Hyytiälä, Finland. Boreal Environment Research, 10(5), 323.

Dall´Osto, M., Beddows, D. C. S., Tunved, P., Krejci, R., Ström, J., Hansson, H.-C., Yoon, Y. J., Park, K.-T., Becagli, S., Udisti, R., Onasch, T., O´Dowd, C. D., Simó, R., & Harrison, R. M. (2017). Arctic sea ice melt leads to atmospheric new particle formation. Scientific Reports, 7(1), 3318. https://doi.org/10.1038/s41598-017-03328-1

Dall´Osto, M., Geels, C., Beddows, D. C. S., Boertmann, D., Lange, R., Nøjgaard, J. K., Harrison, Roy. M., Simo, R., Skov, H., & Massling, A. (2018). Regions of open water and melting sea ice drive new particle formation in North East Greenland. Scientific Reports, 8(1). https://doi.org/10.1038/s41598-018-24426-8

Dasarathy, S., Kar, J., Tackett, J., Rodier, S. D., Lu, X., Vaughan, M., Toth, T. D., Trepte, C., & Bowman, J. S. (2021). Multi-Year Seasonal Trends in Sea Ice, Chlorophyll Concentration, and Marine Aerosol Optical Depth in the Bellingshausen Sea. Journal of Geophysical Research: Atmospheres, 126(21), e2021JD034737. https://doi.org/10.1029/2021JD034737

Dasarathy, S., Russell, L. M., Rodier, S. D., & Bowman, J. S. (2023). Wind-Driven and Seasonal Effects on Marine Aerosol Production in the Bellingshausen Sea, Antarctica. Geophysical Research Letters, 50(3), e2022GL099723. https://doi.org/10.1029/2022GL099723

Freud, E., Krejci, R., Tunved, P., Leaitch, R., Nguyen, Q. T., Massling, A., Skov, H., & Barrie, L. (2017). Pan-Arctic aerosol number size distributions: Seasonality and transport patterns. Atmospheric Chemistry and Physics, 17(13), 8101–8128. https://doi.org/10.5194/acp-17-8101-2017

He, X.-C., Tham, Y. J., Dada, L., Wang, M., Finkenzeller, H., Stolzenburg, D., Iyer, S., Simon, M., Kürten, A., Shen, J., Rörup, B., Rissanen, M., Schobesberger, S., Baalbaki, R., Wang, D. S., Koenig, T. K., Jokinen, T., Sarnela, N., Beck, L. J., … Sipilä, M. (2021). Role of iodine oxoacids in atmospheric aerosol nucleation. Science, 371(6529), 589–595. https://doi.org/10.1126/science.abe0298

Heintzenberg, J., Tunved, P., Galí, M., & Leck, C. (2017). New particle formation in the Svalbard region 2006–2015. Atmospheric Chemistry and Physics, 17(10), 6153–6175. https://doi.org/10.5194/acp-17-6153-2017

Heslin-Rees, D., Tunved, P., Ström, J., Cremer, R., Zieger, P., Riipinen, I., Ekman, A. M. L., Eleftheriadis, K., & Krejci, R. (2024). Increase in precipitation scavenging contributes to long-term reductions of light-absorbing aerosol in the Arctic. Atmospheric Chemistry and Physics, 24(4), 2059–2075. https://doi.org/10.5194/acp-24-2059-2024

Jokinen, T., Lehtipalo, K., Thakur, R. C., Ylivinkka, I., Neitola, K., Sarnela, N., Laitinen, T., Kulmala, M., Petäjä, T., & Sipilä, M. (2022). Measurement report: Long-term measurements of aerosol precursor concentrations in the Finnish subarctic boreal forest. Atmospheric Chemistry and Physics, 22(4), 2237–2254. https://doi.org/10.5194/acp-22-2237-2022

Karlsson, L., Baccarini, A., Duplessis, P., Baumgardner, D., Brooks, I. M., Chang, R. Y. -W., Dada, L., Dällenbach, K. R., Heikkinen, L., Krejci, R., Leaitch, W. R., Leck, C., Partridge, D. G., Salter, M. E., Wernli, H., Wheeler, M. J., Schmale, J., & Zieger, P. (2022). Physical and Chemical Properties of Cloud Droplet Residuals and Aerosol Particles During the Arctic Ocean 2018 Expedition. Journal of Geophysical Research: Atmospheres, 127(11), e2021JD036383. https://doi.org/10.1029/2021JD036383

Kecorius, S., Vogl, T., Paasonen, P., Lampilahti, J., Rothenberg, D., Wex, H., Zeppenfeld, S., Van Pinxteren, M., Hartmann, M., Henning, S., Gong, X., Welti, A., Kulmala, M., Stratmann, F., Herrmann, H., & Wiedensohler, A. (2019). New particle formation and its effect on cloud condensation nuclei abundance in the summer Arctic: A case study in the Fram

Strait and Barents Sea. Atmospheric Chemistry and Physics, 19(22), 14339–14364. https://doi.org/10.5194/acp-19-14339-2019

Kerminen, V.-M., Chen, X., Vakkari, V., Petäjä, T., Kulmala, M., & Bianchi, F. (2018). Atmospheric new particle formation and growth: Review of field observations. Environmental Research Letters, 13(10), 103003. https://doi.org/10.1088/1748-9326/aadf3c

Kulmala, M., Arola, A., Nieminen, T., Riuttanen, L., Sogacheva, L., De Leeuw, G., Kerminen, V.-M., & Lehtinen, K. E. J. (2011). The first estimates of global nucleation mode aerosol concentrations based on satellite measurements. Atmospheric Chemistry and Physics, 11(21), 10791–10801. https://doi.org/10.5194/acp-11-10791-2011

Kulmala, M., Junninen, H., Dada, L., Salma, I., Weidinger, T., Thén, W., Vörösmarty, M., Komsaare, K., Stolzenburg, D., Cai, R., Yan, C., Li, X., Deng, C., Jiang, J., Petäjä, T., Nieminen, T., & Kerminen, V.-M. (2022). Quiet New Particle Formation in the Atmosphere. Frontiers in Environmental Science, 10, 912385. https://doi.org/10.3389/fenvs.2022.912385

Kyrö, E.-M., Väänänen, R., Kerminen, V.-M., Virkkula, A., Petäjä, T., Asmi, A., Dal Maso, M., Nieminen, T., Juhola, S., Shcherbinin, A., Riipinen, I., Lehtipalo, K., Keronen, P., Aalto, P. P., Hari, P., & Kulmala, M. (2014). Trends in new particle formation in eastern Lapland, Finland: Effect of decreasing sulfur emissions from Kola Peninsula. Atmospheric Chemistry and Physics, 14(9), 4383–4396. https://doi.org/10.5194/acp-14-4383-2014

Lampilahti, J., Paasonen, P., Tuovinen, S., Lehtipalo, K., Kerminen, V.-M., & Kulmala, M. (2025). A cross-correlation-based method for determining size-resolved particle growth rates. Aerosol Research, 3(2), 637–647. https://doi.org/10.5194/ar-3-637-2025

Li, X., Nieminen, T., Baalbaki, R., Zhou, P., Paasonen, P., Makkonen, R., Zaidan, M. A., Sarnela, N., Yan, C., Jokinen, T., Salma, I., Vörösmarty, M., Petäjä, T., Kerminen, V.-M., Kulmala, M., & Dada, L. (2025). Parameterization of particle formation rates in distinct atmospheric environments. Aerosol Research, 3(1), 271–291. https://doi.org/10.5194/ar-3-271-2025

Mackie, A. R., Palmer, P. I., Barlow, J. M., Finch, D. P., Novelli, P., & Jaeglé, L. (2016). Reduced Arctic air pollution due to decreasing European and North American emissions. Journal of Geophysical Research: Atmospheres, 121(14), 8692–8700. https://doi.org/10.1002/2016jd024923

Meier, W., Fetterer, F., Windnagel, A., Stewart, J. S., & Stafford, T. (2024). NOAA/NSIDC Climate Data Record of Passive Microwave Sea Ice Concentration, Version 5 [Dataset]. National Snow and Ice Data Center. https://doi.org/10.7265/RJZB-PF78

Nguyen, Q. T., Glasius, M., Sørensen, L. L., Jensen, B., Skov, H., Birmili, W., Wiedensohler, A., Kristensson, A., Nøjgaard, J. K., & Massling, A. (2016). Seasonal variation of atmospheric particle number concentrations, newparticle formation and atmospheric oxidation capacity at the high Arcticsite Villum Research Station, Station Nord. Atmospheric Chemistry and Physics, 16(17), 11319–11336. https://doi.org/10.5194/acp-16-11319-2016

Pernov, J. B., Beddows, D., Thomas, D. C., Dall´Osto, M., Harrison, R. M., Schmale, J., Skov, H., & Massling, A. (2022). Increased aerosol concentrations in the High Arctic attributable to changing atmospheric transport patterns. Npj Climate and Atmospheric Science, 5(1), 62. https://doi.org/10.1038/s41612-022-00286-y

Pernov, J. B., Harris, E., Volpi, M., Baumgartner, T., Hohermuth, B., Henne, S., Aeberhard, W. H., Becagli, S., Quinn, P. K., Traversi, R., Upchurch, L. M., & Schmale, J. (2024). Pan-Arctic methanesulfonic acid aerosol: Source regions, atmospheric drivers, and future projections.

Npj Climate and Atmospheric Science, 7(1), 166. https://doi.org/10.1038/s41612-024-00712-3

Petäjä, T., Mauldin, Iii, R. L., Kosciuch, E., McGrath, J., Nieminen, T., Paasonen, P., Boy, M., Adamov, A., Kotiaho, T., & Kulmala, M. (2009). Sulfuric acid and OH concentrations in a boreal forest site. Atmospheric Chemistry and Physics, 9(19), 7435–7448. https://doi.org/10.5194/acp-9-7435-2009

Prenni, A. J., Harrington, J. Y., Tjernström, M., DeMott, P. J., Avramov, A., Long, C. N., Kreidenweis, S. M., Olsson, P. Q., & Verlinde, J. (2007). Can Ice-Nucleating Aerosols Affect Arctic Seasonal Climate? Bulletin of the American Meteorological Society, 88(4), 541–550. https://doi.org/10.1175/BAMS-88-4-541

Schmale, J., Sharma, S., Decesari, S., Pernov, J., Massling, A., Hansson, H.-C., Von Salzen, K., Skov, H., Andrews, E., Quinn, P. K., Upchurch, L. M., Eleftheriadis, K., Traversi, R., Gilardoni, S., Mazzola, M., Laing, J., & Hopke, P. (2022). Pan-Arctic seasonal cycles and long-term trends of aerosol properties from 10 observatories. Atmospheric Chemistry and Physics, 22(5), 3067–3096. https://doi.org/10.5194/acp-22-3067-2022

Schmale, J., Zieger, P., & Ekman, A. M. L. (2021). Aerosols in current and future Arctic climate. Nature Climate Change, 11(2), 95–105. https://doi.org/10.1038/s41558-020-00969-5

Stevens, R. G., Loewe, K., Dearden, C., Dimitrelos, A., Possner, A., Eirund, G. K., Raatikainen, T., Hill, A. A., Shipway, B. J., Wilkinson, J., Romakkaniemi, S., Tonttila, J., Laaksonen, A., Korhonen, H., Connolly, P., Lohmann, U., Hoose, C., Ekman, A. M. L., Carslaw, K. S., & Field, P. R. (2018). A model intercomparison of CCN-limited tenuous clouds in the high Arctic. Atmospheric Chemistry and Physics, 18(15), 11041–11071. https://doi.org/10.5194/acp-18-11041-2018

Sun, D., Ji, C., Sun, W., Yang, Y., & Wang, H. (2018). Accuracy assessment of three remote sensing shortwave radiation products in the Arctic. Atmospheric Research, 212, 296–308. https://doi.org/10.1016/j.atmosres.2018.01.003

Sundström, A.-M., Nikandrova, A., Atlaskina, K., Nieminen, T., Vakkari, V., Laakso, L., Beukes, J. P., Arola, A., Van Zyl, P. G., Josipovic, M., Venter, A. D., Jaars, K., Pienaar, J. J., Piketh, S., Wiedensohler, A., Chiloane, E. K., De Leeuw, G., & Kulmala, M. (2015). Characterization of satellite-based proxies for estimating nucleation mode particles over South Africa. Atmospheric Chemistry and Physics, 15(9), 4983–4996. https://doi.org/10.5194/acp-15-4983-2015

Tesche, M., Zieger, P., Rastak, N., Charlson, R. J., Glantz, P., Tunved, P., & Hansson, H.-C. (2014). Reconciling aerosol light extinction measurements from spaceborne lidar observations and in situ measurements in the Arctic. Atmospheric Chemistry and Physics, 14(15), 7869–7882. https://doi.org/10.5194/acp-14-7869-2014

Tham, Y. J., Sarnela, N., Iyer, S., Li, Q., Angot, H., Quéléver, L. L. J., Beck, I., Laurila, T., Beck, L. J., Boyer, M., Carmona-García, J., Borrego-Sánchez, A., Roca-Sanjuán, D., Peräkylä, O., Thakur, R. C., He, X.-C., Zha, Q., Howard, D., Blomquist, B., … Saiz-Lopez, A. (2023). Widespread detection of chlorine oxyacids in the Arctic atmosphere. Nature Communications, 14(1), 1769. https://doi.org/10.1038/s41467-023-37387-y

Tunved, P., Ström, J., & Krejci, R. (2013). Arctic aerosol life cycle: Linking aerosol size distributions observed between 2000 and 2010 with air mass transport and precipitation at Zeppelin station, Ny-Ålesund, Svalbard. Atmospheric Chemistry and Physics, 13(7), 3643–3660. https://doi.org/10.5194/acp-13-3643-2013

Väänänen, R., Kyrö, E.-M., Nieminen, T., Kivekäs, N., Junninen, H., Virkkula, A., Dal Maso, M., Lihavainen, H., Viisanen, Y., Svenningsson, B., Holst, T., Arneth, A., Aalto, P. P., Kulmala, M., & Kerminen, V.-M. (2013). Analysis of particle size distribution changes between three

measurement sites in northern Scandinavia. Atmospheric Chemistry and Physics, 13(23), 11887–11903. https://doi.org/10.5194/acp-13-11887-2013

Wang, G., Wang, T., & Xue, H. (2021). Validation and comparison of surface shortwave and longwave radiation products over the three poles. International Journal of Applied Earth Observation and Geoinformation, 104, 102538. https://doi.org/10.1016/j.jag.2021.102538

Xi, X., Yang, Q., Liu, C., Shupe, M. D., Han, B., Peng, S., Zhou, S., & Chen, D. (2024). Evaluation of the Planetary Boundary Layer Height From ERA5 Reanalysis With MOSAiC Observations Over the Arctic Ocean. Journal of Geophysical Research: Atmospheres, 129(12), e2024JD040779. https://doi.org/10.1029/2024JD040779

Yao, W., Gui, K., Zheng, Y., Li, L., Wang, Y., Che, H., & Zhang, X. (2023). Seasonal cycles and long-term trends of arctic tropospheric aerosols based on CALIPSO lidar observations. Environmental Research, 216, 114613. https://doi.org/10.1016/j.envres.2022.114613

Zhang, K., Zhao, L., Tang, W., Yang, K., & Wang, J. (2022). Global and Regional Evaluation of the CERES Edition-4A Surface Solar Radiation and Its Uncertainty Quantification. IEEE Journal of Selected Topics in Applied Earth Observations and Remote Sensing, 15, 2971–2985. https://doi.org/10.1109/JSTARS.2022.3164471

We thank the reviewers for the comments and have made the edits accordingly. The original comments are in blue, and text from the manuscript are in quotes.

Reviewer #1:

Thank you for considering my comments and for revising the manuscript accordingly. The work is much improved thanks to the added details and revised descriptions.
There is still one major issue, though, which is about the use of the high emission scenario SSP5-8.5 in the presentation of the findings an their discussion. As this scenario was recently dismissed by the scientific community as unrealistic, I suggest that any results related to it are omitted from the paper and that the work should focus on findings related to SSP2-4.5 instead.

Thank you for the feedback. We acknowledge the issue with the SSP5-8.5 scenario and have removed this scenario in our analysis. The changes to the text are in below:

In abstract:

> "Temperature-sensitivity estimates based on the intermediate future warming scenario (SSP2-4.5) suggest that by 2090-2100, cloud condensation nuclei concentrations associated with new particle formation activity could increase by 3-16% in the High Arctic but decline by 15-31% in the Maritime Arctic."

Figure 2 now looks:

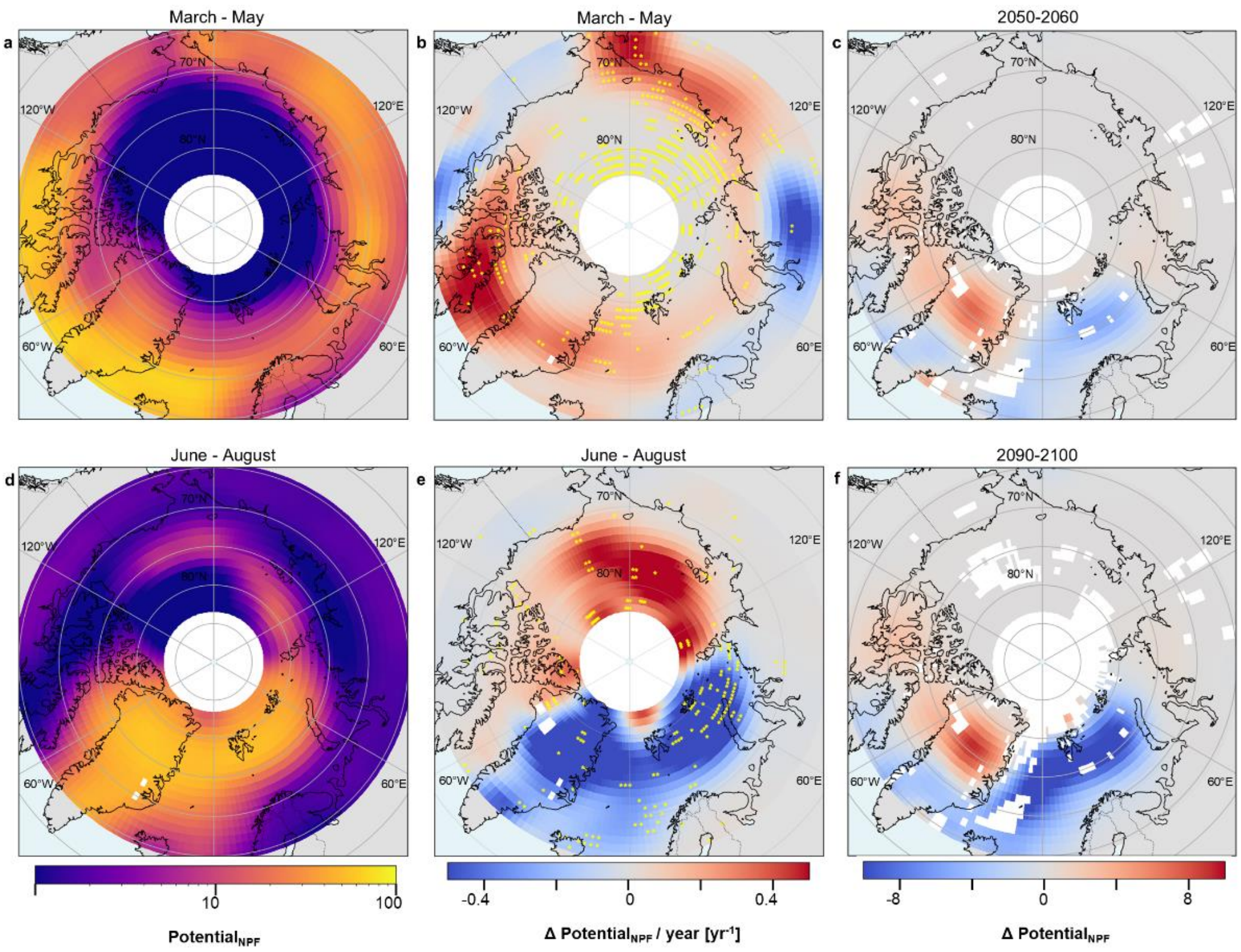

Figure 2. NPF potential ($Potential_{NPF}$), historical trends, and future projections across the Arctic. Medians of $Potential_{NPF}$ averaged over 2006-2021 as estimated from satellite-based proxies during (a) March-May and (d) June-August. $Potential_{NPF}$ ranged (0,100) with 0 and 100 representing infinitely low and high potential of NPF events

occurring respectively. Yearly change in $Potential_{NPF}$ over 2006-2021 during months of (b) March-May and (e) June-August expressed as percentage change per year. Pixels where trend is statistically significant (Mann-Kendall seasonal test, $p < 0.1$) are marked by yellow dots. Temperature-sensitivity estimate of change in $Potential_{NPF}$ by (c) 2050-2060 and (f) 2090-2100 relative to the 2006–2021 baseline period, considering only the projected temperature change under the SSP2-4.5 scenario. White pixels indicate areas where data was unavailable.

In the discussion:

"… change in $Potential_{NPF}$ was computed using the ensemble projected temperatures from the SSP 2-4.5 intermediate emissions scenario. The changes in $Potential_{NPF}$, following temperature changes by mid-century (2050-2060; Figure 2c) or end-of-century (2090-2100; Figure 2f), showed spatial variation relative to the 2006–2021 baseline period."

"…N_{CCN} could increase by up to 11% (95% CI: 3– 16%) by 2090-2100 with the expected temperature change under the SSP 2-4.5 scenario (Figure 4b). … the estimated change in N_{CCN} in the Maritime Arctic, using a similar extrapolation of current N_{CCN}-$Potential_{NPF}$ relationship, showed as much as a 30% (95% CI: 15– 31%) decline by 2090-2100 under SSP 2-4.5…"

Tables S3 in Supporting Information:

Table S3: Area-weighted median values of estimated change in $Potential_{NPF}$ in 2050-2060 and 2090-2100 under the SSP2-4.5 climate scenario compared to its baseline level (2006-2021). The variability range indicate the interquartile range of possible outcomes while the confidence range indicate 95% confidence interval around the median value. Refer to Text S4 for more details on the uncertainty ranges.

	2050-2060			2090-2100		
		Variability range	Confidence range		Variability range	Confidence range
High Arctic	0.6	-3.3 – 9.5	0.1 – 1.3	0.9	-2.6 – 10.8	0.7 – 1.9
Maritime Arctic	-3.4	-11.8 – 8.7	-5.9 – -2.9	-6.6	-13.0 – 0.2	-8.3 – -6.5
Continental Arctic	0.0	-0.5 – 1.4	-0.1 – 0.1	0.0	-0.6 – 1.4	-0.1 – 0.1

Reviewer #2:

Thank you to the authors for addressing my comments. They took my concerns seriously and made substantial improvements to the manuscript. In particular, they evaluated the PotentialNPF metric against observations and significantly improved its estimation using a machine learning approach with additional predictors, including temperature.

The results and conclusions are now much more robust, and provide valuable insights into future CCN changes associated with NPF. I am very happy to give the manuscript a green light for publication.

Thank you for the positive response and the detailed comments that certainly helped to improve the manuscript!

Reviewer #3:

The manuscript has been revised according to the suggestions of all three reviewers. I think the changes improve the argumentation structure and comprehensibility and reduce the number of unclear formulations.
Regarding statistics, box plots present the distributions, and error bars are included where appropriate. Uncertainties are also mentioned in the text.
I consider the manuscript suitable for publication in Nature Geoscience and do not need to see a further revision.

Thank you for your time in the re-reading. We have made the changes to the text as follows:

I have the following minor points and typos to correct:

L. 32: I believe you mean the Maritime Arctic, i.e., use "the" instead of "and."

As the abstract has been rewritten following the editorial guidelines, the correction in the line above is no longer relevant.

L. 104: Remove the colon.

Sentence now reads

“During June-August, and to a smaller extent, March-May, local processes…”

L. 122: "at" appears twice in "where observed at only at a few sites"; please remove one.

Sentence reads:

“…positive trends (p<0.1) in $\Delta N_{12.5-15}$ were observed only at a few sites…”

L. 123 ff: Please explain the meaning of the brackets.

The range of values refer to IQR. The brackets and labels have been changed to:

“…Alert (26 [IQR: 20 – 32] % yr-1), Villum (19 [IQR: 16 – 22] % yr-1) and Zeppelin (12 [IQR: 7.5 – 17] % yr-1)…”

L. 164: Insert a comma before "too."

We removed the “too” as it sounds ungrammatical in this context.

L. 169: How do you know that the air is "clean"? Please explain.

Perhaps “clean” is not an appropriate word choice here as what we meant was that the air was not carrying mainly transported aerosols from other sources, such as dust or soot from lower latitudes. There is a longer elaboration in SI Text S3 which we now point the reader to. Sentence now reads:

“…N_{CCN} values presented here could be considered representative of that from natural background conditions (more information of CCN retrievals in SI Text S3).”

Fig. 3: Please write out the abbreviation "SS" — I assume it stands for supersaturation, but it was not introduced earlier.

Corrected. Caption now reads:

“(c) CCN number concentration at supersaturation (SS) = 0.2% (N_{CCN}) from clean continental and marine aerosols…”

Fig. 4: In the fourth line before "may increase" and "may decrease," please remove the dots for High Arctic and Maritime Arctic, as they appear to belong with the preceding line.

Thank you for pointing this out! Corrected.

L. 403: Please italicize "PotentialNPF" and format "NPF" as a subscript/index.

Corrected.

“The derived values were then transformed into $Potential_{NPF}$ maps using Eq. 1b.”
